# Liquid Crystal Monomers Released from LCD Displays Accumulate in Endangered Marine Cetaceans Triggering Health Concerns

**DOI:** 10.1021/acs.est.5c17767

**Published:** 2026-02-25

**Authors:** Danyang Tao, Chengzhang Li, Yajing Sun, Yuefei Ruan, Qianqian Jin, Jiaji Sun, Yichun Lu, Brian C. W. Kot, Paul K. S. Lam, Fengchang Wu, Jia He, John P. Giesy, Kurunthachalam Kannan, Bo Liang, Wenhua Liu, Lin Zhang, Yunsong Mu, Kenneth M. Y. Leung, Yuhe He

**Affiliations:** † State Key Laboratory of Marine Environmental Health and Department of Chemistry, 53025 City University of Hong Kong, Kowloon, Hong Kong SAR, China; ‡ Guangdong Provincial Key Laboratory of Marine Disaster Prediction and Prevention and Guangdong Provincial Key Laboratory of Marine Biotechnology, Institute of Marine Sciences, 12386 Shantou University, Shantou 515063, China; § School of Energy and Environment and State Key Laboratory of Marine Environmental Health, City University of Hong Kong, Kowloon, Hong Kong SAR, China; ∥ Research Centre for the Oceans and Human Health, City University of Hong Kong Shenzhen Research Institute, Shenzhen 518057, China; ⊥ Department of Science, School of Science and Technology, 597314 Hong Kong Metropolitan University, Kowloon, Hong Kong SAR, China; # State Key Laboratory of Environmental Criteria and Risk Assessment, Chinese Research Academy of Environmental Sciences, Beijing 100012, China; ∇ Beijing Key Laboratory of Urban Hydrological Cycle and Sponge City Technology, College of Water Sciences, 47836 Beijing Normal University, Beijing 100875, China; ○ Department of Veterinary Biomedical Sciences and Toxicology Centre, University of Saskatchewan, Saskatoon, SK S7N 5B3, Canada; ◆ Department of Environmental Sciences, Baylor University, Waco, Texas 76798-7266, United States; ¶ Department of Integrative Biology, Michigan State University, East Lansing Michigan 48895, United States; ■ Wadsworth Center, New York State Department of Health, Albany, New York 12237, USA and Department of Environmental Health Sciences, State University of New York at Albany, Albany, New York 12237, United States; ⬢ School of Chemistry and Life Resources, 12471 Renmin University of China, Beijing 100872, China

**Keywords:** Liquid crystal monomers (LCMs), Tissue enrichment, Indo-Pacific cetaceans, DNA damage, E-waste

## Abstract

Liquid crystal monomers (LCMs), critical substances of liquid crystal displays in consumer electronics, are persistent pollutants, posing potential threats to marine ecosystems. Despite their bioaccumulative potential, their occurrence and possible biological impacts on marine megafauna remain understudied. We investigated LCM occurrence in Indo-Pacific humpback dolphins (*Sousa chinensis*) and finless porpoises (*Neophocaena phocaenoides*) collected from the South China Sea (2007–2021) and assessed their toxicity through *in vitro* assays using established dolphin cell lines. By employing robust source-tracing methodologies, we provide the first evidence that LCMs from household electronics and coastal e-waste accumulate in cetacean tissues, including blubber, muscle, and, critically, brain tissues, demonstrating blood–brain barrier penetration, a previously undocumented phenomenon of LCMs in mammalian wildlife. The temporal trend of LCM burden in porpoise blubber is correlated with shifts in global liquid crystal display production. Transcriptomic profiling revealed LCM-induced DNA damage, cell cycle arrest, and impaired cell division in cetacean cells. These findings suggest that LCMs may pose potential risks to the nervous system and other organs of marine mammals, warranting further investigation into their toxicological effects and possible implications for human health. By bridging critical gaps among everyday electronics, LCM contamination, and marine conservation, this study highlights the need for urgent regulatory actions and improved e-waste governance to mitigate ecological and public health risks.

## Graphical abstract

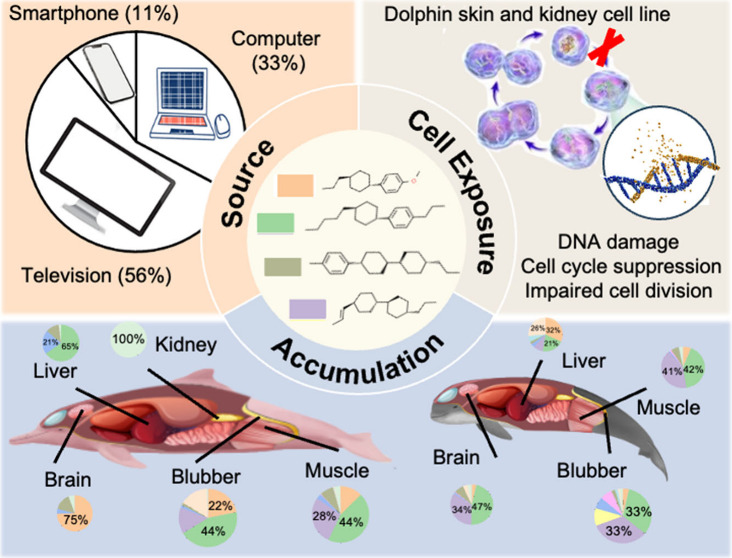

## Introduction

Liquid crystal monomers (LCMs) are used in the production of liquid crystal display (LCD) panels in electronic devices (e-devices).^1^ It is estimated that global LCD panel production will reach 238 million m^2^ by 2025,^2^ and approximately 74 million tons of LCD devices will be discarded annually as electronic waste (e-waste) by 2030.^3^ The widespread use and improper disposal of these devices can lead to the release of LCMs into the environment. The distinctive chemical structure of LCMs imparts persistence, bioaccumulation, and toxicity (PBT) properties,^4,5^ allowing them to potentially accumulate in the environment and pose risks to the health or fitness of animals and humans.

Since 2019, studies have documented the occurrence of LCMs in air, dust, wastewater, and sediment from countries such as China, Sweden, and the United States.^6–11^ Despite this, information about concentrations in various matrices including wildlife and humans is scarce. A total of 29 organic light-emitting materials were detected in aquatic organisms collected from coastal areas of China, including 16 LCMs in both invertebrates and fishes, with individual concentrations at the ng/g wet weight (ww) level.^12^ Another study from South China reported Σ_10_LCMs and Σ_29_LCMs concentrations in serum from urban residents and e-waste dismantling workers, at ranges of 3.16–28.5 (median: 9.59) ng/mL and 7.78–276 (median: 35.2) ng/mL, respectively.^13^ Thirty fluorinated LCMs were found in the breast milk of Beijing residents, with Σ_30_LCMs concentrations ranging from 12.0 to 28,200 (median: 133) ng/g lipid mass (lm).^14^ However, most existing studies focus on environmental matrices and lower-trophic organisms (invertebrates/fishes) or human biomonitoring, and evidence of top predators is still lacking. The occurrence of LCMs in wildlife, especially marine mammals, is not currently known. In particular, tissue-resolved data in wild cetaceans and mechanistic toxicity information based on cetacean-relevant cells remain largely absent.

The Pear River Estuary (PRE) of the northern South China Sea (SCS) is among the most urbanized and industrialized areas globally and is a habitat for protected rare marine cetaceans, such as Indo-Pacific humpback dolphin (*Sousa chinensis*, *SC*) and the Indo-Pacific finless porpoise (*Neophocaena phocaenoides*, *NP*).^15^ This area is heavily impacted by urban runoff, which carries significant quantities of legacy and emerging anthropogenic organic contaminants, such as polychlorinated biphenyls (PCBs), polybrominated diphenyl ethers (PBDEs), and per- and polyfluoroalkyl substances (PFASs), among others.^16–19^ Our previous research has demonstrated that municipal wastewater effluent is a significant source of LCMs, contributing to their occurrence in sediments from the South China Sea region.^7,20^ Studies indicated that dolphins and porpoises in the SCS are exposed to elevated levels of PFASs and PBDEs.^21,22^ However, significant knowledge gaps exist regarding the tissue accumulation and distribution of LCMs in marine mammals, particularly in cetaceans.

Recent research revealed the potential that LCMs are capable of accumulating in experimental animal tissues, including adipose tissue, muscle, and, importantly, the brain,^23,24^ exhibiting various toxic effects. For example, zebrafish larvae exposed to LCMs showed disruption of photoreceptor patterning, with potential involvement of thyroid hormone signaling,^25^ and in *Daphnia magna*, LCMs have been identified as novel endocrine-disrupting chemicals.^26^ However, while recent studies in rodent models have revealed testicular, renal, and hepatic toxicities of certain LCMs through diverse molecular pathways,^27–29^ significant knowledge gaps remain regarding their accumulation and toxicological effects in mammalian megafauna. These deficiencies can compromise the assessment and management of ecological risks to vulnerable marine cetaceans living in sensitive areas, such as dolphins in the northern SCS.

This study aimed to assess exposure and risks associated with LCMs in marine mammals by (1) investigating the occurrence of emerging LCMs in tissues of two marine cetaceans, *SC* and *NP*, collected in the northern SCS; (2) analyzing temporal trends (2007–2021) and determinants of LCM accumulation in *NP*; and (3) conducting a toxicity assessment of select LCMs using established cetacean cell lines. The results provide evidence-based insights into the extent of LCM contamination in the northern SCS and shed new light on the comprehensive assessment of the ecological and health impacts associated with these emerging contaminants related to e-devices and e-waste.

## Materials and Methods

### Chemicals

A total of 62 target LCMs were procured from Tokyo Chemical Industry Co., Ltd. (Hong Kong, China), J&K Chemical Ltd. (Shanghai, China), and LCM manufacturers (Table S1). Since authentic standards containing stable isotopes of LCMs were not commercially available, isotope-labeled PCB-118 (^13^C_12_-2,3′,4,4′,5-penta­chloro­biphenyl) and BDE-77 (^13^C_12_-3,3′,4,4′-tetra­bromo­diphenyl ether) were used as surrogate standards for LCMs.^4,30^ These compounds were selected because their chemical structures and physicochemical properties, including aromatic backbones and halogenation patterns, are broadly similar to those of LCMs, allowing them to reasonably mimic the LCM behavior during extraction, cleanup, and instrumental analysis. High-performance liquid chromatography (HPLC)-grade dichloromethane (DCM) was purchased from Merck KGaA (Darmstadt, Germany).

### Sample Collection

This study used tissue samples from stranded corpses of *SC* and *NP* collected by the Agriculture, Fisheries, and Conservation Department (AFCD) of the Government of Hong Kong Special Administrative Region, China; not all tissues could be obtained from every individual because carcasses were often partially decomposed upon discovery (Table S2). Sample collection was conducted under the permission of AFCD (Ref No. AF MCW CON 01/4 PT.4). No live animals were harmed for sample collection. Descriptions of two marine cetaceans are provided in Figure S1 and Text S1. The samples comprised 16 individuals of *SC* and 26 individuals of *NP*, including various tissues such as kidney, melon, liver, brain, blubber, and muscle tissues, for subsequent analyses (Table S2). For the blubber samples used in the temporal trend analysis, 48 *NP* individuals were collected (Table S3). Upon arrival at the laboratory, all *SC* and *NP* samples were freeze-dried and stored at −20 °C until further processing. For establishment of cetacean cell lines, skin and kidney tissues were obtained from a stranded melon-headed whale (*Peponocephala electra*). The study protocols were reviewed and approved by the Animal Ethics Committee of Shantou University Medical College, granting the special exempt “Possible causes of death of marine mammals, from 2022.01-2027.12)”. All participants gave their informed consent.

### LCM Analysis in Marine Cetacean Tissue

All collection and weighing tools were rinsed thoroughly with DCM and methanol prior to use to minimize any potential carryover or cross-contamination. During each batch of sample analysis, procedural blanks, field blanks, matrix blanks, and independent calibration checks were processed in parallel to monitor contamination and instrument drift. All sample extracts were injected within 48 h after extraction. Extraction of LCMs was accomplished by use of previously published methods.^30^ Briefly, approximately 0.5 g of tissue sample, measured in dry weight (dm), was spiked with ^13^C_12_-PCB-118 and ^13^C_12_-BDE-77 at a level of 10 ng/g and then transferred to a 15 mL polypropylene tube for equilibration (30 min). Next, 5 mL of dichloromethane (DCM) was added to each sample, followed by ultrasonic extraction for 30 min (480 W, at room temperature). The sample was then centrifuged at approximately 15,000*g* for 10 min to collect the supernatant. This procedure was repeated three consecutive times, each with 5 mL of DCM and ultrasonic treatment. The resulting extracts were combined, and the supernatants (approximately 10 mL) were concentrated by a gentle stream of high-purity nitrogen to near dryness, followed by solvent exchange to 1 mL of DCM. For extract purification, a self-packed column was assembled with 3 g of Florisil (deactivated with 5% water), 1 g of alumina, copper powder (for liver and brain samples), and anhydrous sodium sulfate from bottom to top. The column was preconditioned with 10 mL of DCM, and the target LCMs were eluted with 20 mL of DCM. The eluate was concentrated to near dryness and reconstituted with 200 μL of DCM for instrumental analysis. LCM analysis in cell culture is presented in Text S2.

Target LCMs were quantified using a Thermo Fisher Scientific 220 Trace 1300 gas chromatograph (GC) coupled with a Q Exactive Orbitrap hybrid quadrupole mass spectrometer (MS/MS) (Thermo Fisher Scientific). The MS was operated in electron impact (EI) mode with a full-mass scan. Separation was performed using a DB-5HT column (30 m × 0.25 mm × 0.1 μm; Agilent). The injection was performed in splitless mode at an injector temperature of 285 °C, with a carrier gas flow rate of 1.2 mL/min. The temperature program started at 40 °C for 1 min, increased to 180 °C at 40 °C/min, and then further increased to 250 °C at 30 °C/min, held for 2 min, ramped to 300 °C at 10 °C/min, and held for 5 min. The injection volume was 1 μL. The ion source temperature and transfer line temperature were 290 and 260 °C, respectively, with an ion source filament voltage of 70 eV. Quantification and confirmation ions and retention times of LCM analytes are listed in Table S4. Key QA/QC measures included procedural blanks, matrix-spiked recoveries, and instrument performance checks, and no target analytes were detected in blanks above the reporting thresholds. Details of validation parameters and spike recoveries are provided in Figure S2 and Tables S5–S7. Suspect screening methods are shown in Text S3.

### Predictive Toxicity Assessment Using OECD QSAR Toolbox

The eight priority LCMs MOPrCHB, PeCHPrB, EDFPPB, EBMB, MPCB, PPB, MPhBB, and PCTB (Table S8) were predicted using OECD QSAR Toolbox v4.4.1. Chemical structures were analyzed with the rat liver S9 metabolic simulator, and end points including acute toxicity, DNA/protein binding, mutagenicity, and carcinogenicity were predicted. Compounds were also classified by PBT and Cramer criteria.

### Transcriptomic Analysis and qPCR Validation

The Melon-Head Skin Fibroblast (MHSF) and Melon-Head Kidney Fibroblast (MHKF) cells were primary (nonimmortalized) fibroblast-enriched cultures derived from skin and kidney tissues of a melon-headed whale (*Peponocephala electra*); isolation and culture procedures are described in Text S4.^31^ Transcriptomic analyses were conducted to evaluate the potential health risks associated with exposure to individual LCMs by using MHSF and MHKF primary cell cultures. Eight priority LCMs (Table S9), which exhibited the highest detection frequencies in dolphin and porpoise samples, were selected. The MHSF and MHKF cells were treated with LCMs in triplicate when the cell confluence reached 70%. For each priority LCM, two exposure concentrations (i.e., low and high) were selected based on the determined concentrations in cetacean samples (Table S9). Vehicle controls containing the same final solvent concentration as the corresponding treatments were included to account for solvent effects (final 0.2% v/v ethanol or 0.2% v/v DMSO, depending on the compound). Both ethanol and DMSO were used as vehicles and were independently evaluated using matched vehicle controls with the solvent fraction kept constant across the full concentration series. For the cell viability assay, cells were seeded and tested in 96-well plates (Text S5). After a 24 h treatment, cells were harvested, and RNA was isolated using Trizol following the manufacturer’s instructions. For each treatment condition, 3–4 wells were included as technical replicates to ensure consistency of the assay. All RNA samples were derived from this single biological source (skin and kidney cells from one melon-headed whale), and RNA sequencing was performed on these technical replicates using an Illumina Hiseq6000 platform. We note that no biological replicates were included due to the limited availability of cetacean primary cells, and therefore, the results reflect the response of this particular biological sample. Lesser-quality reads and adapters were removed using trim-galore.^32^ Clean reads were aligned to the genome the chromosome-level genome assembly of *Peponocephala electra* (melon-headed whale) using STAR software,^33,34^ and transcripts were quantified with R-package RSEM (Robust Structural Equation Modeling).^35^ Gene expression levels were estimated using raw count data (not normalized values, such as FPKM). Raw counts were used as the inputs for DESeq2. Differential expression analysis was conducted using DESeq2,^36^ considering only genes with |fold change| ≥ 2 and false discovery rate (FDR) ≤ 0.05 as differential expression genes (DEGs). Subsequently, functional clustering analysis was performed with Metascape, and a volcano plot was illustrated using scRNAtoolVis.^37,38^ Principal component analysis (PCA) confirmed that intergroup variability (LCM treated vs vehicle control) was greater than intragroup variability (Figure S3). To quantify the relative expression level of 10 DEGs related to cell cycle regulation, qPCR analysis was performed in triplicate with MHSF or MHKF cells after intervention with different LCMs (Text S6).

### Data Treatment and Statistical Analyses

Prior to additional statistical analysis, the Shapiro–Wilk test was performed to determine whether the data met the assumption of parametric tests of being normally distributed. The assumption of homogeneity of variance was tested by Levene’s test. Student’s *t*-test and Pearson correlation analysis were used to evaluate the differences in LCM concentrations between groups, provided that the data met the assumptions. If the data were not normally distributed, the Mann–Whitney U test was performed to evaluate differences between groups, and Spearman rank correlation was utilized to investigate potential significant relationships. For comparisons among more than two groups (e.g., tissue differences within a species), we used Kruskal–Wallis tests (given small sample sizes and non-normality concerns). Statistical analyses were carried out using IBM SPSS 19.0 (USA) 19.0, R studio 4.4.1 (USA), and Origin Pro 2021 (USA). Positive matrix factorization–multiple linear regression (PMF-MLR) analysis is shown in Text S7.

## Results and Discussion

### LCM Accumulation in Marine Cetacean Tissues

The SCS serves as a biodiversity hotspot and a crucial habitat for several marine megafauna species, including the Indo-Pacific humpback dolphins and finless porpoises. As apex predators, these cetaceans are particularly vulnerable to anthropogenic pollutants, which can bioaccumulate within their bodies, affecting their health and survival.^39^ Among the 62 LCMs targeted for analysis, 38 were found in at least one of the 63 samples of tissues analyzed (Figure S4). Concentrations and relative contributions of liquid crystal monomers (LCMs) in different tissues of two kinds of marine mammals are shown in Tables S10 and S11. Additionally, high-resolution mass spectrometry analysis using a database comprising 1173 LCMs derived from patents and published articles revealed the existence of 27 possible molecular structures of suspected LCMs in the cetacean tissues (Figure S5 and Table S12). Among these 27 suspected LCMs, DFEBB and TFPrBB were confirmed. Importantly, these 27 suspected compounds are distinct from the 38 LCMs detected and quantified via targeted analysis; the remaining 25 compounds could only be tentatively assigned based on their possible molecular formulas.

In this study, we provide the first evidence of the accumulation of LCMs, a class of emerging pollutants derived from household electronics and electronic waste, in wild cetaceans. LCMs were found in various tissues of two marine cetaceans, including muscle, brain, blubber, liver, and kidney tissues. The concentrations of ΣLCMs for all tissues ranged from less than the limit of detection (i.e., ND) to 203 ng/g, with mean (±SD) concentrations of 53.6 ± 65.3 ng/g dm (dry mass) in dolphins and 32.7 ± 41.8 ng/g dm in porpoises. The distribution of ΣLCMs across the tissues varied significantly. In dolphins, the highest ΣLCMs concentration was found in blubber (57.9 ± 70.8 ng/g dm) and muscle (66.6 ± 68.5 ng/g dm), followed by the brain (7.75 ± 11.0 ng/g dm), whereas in porpoise samples, the highest ΣLCMs concentration was found in blubber (45.7 ± 53.5 ng/g dm), followed by the brain (38.7 ± 36.8 ng/g dm) and muscle (27.2 ± 31.0 ng/g dm) (Kruskal–Wallis test across tissues within each species; only tissues with *n* ≥ 2 were included in statistical testing; *p* < 0.01) (Figure 1a). Blubber, with its high lipid content, served as the primary reservoir for LCMs, in line with previous observations that adipose tissue (fat) is a major sink for lipophilic contaminants.^23,40,41^ Muscle and brain tissues also contain notable levels of lipids,^42^ explaining the accumulation of LCMs in these organs.^23^ Notably, the detection of LCMs in brain tissue is particularly concerning, as only a limited number of xenobiotics, including persistent organic pollutants (POPs) like PCBs, PBDEs, and PFASs, have been shown to penetrate the blood–brain barrier in marine mammals.^43–45^ This raises potential concerns regarding neurotoxicity, although direct evidence of LCMs in neural tissues is currently lacking.

**Figure 1:**
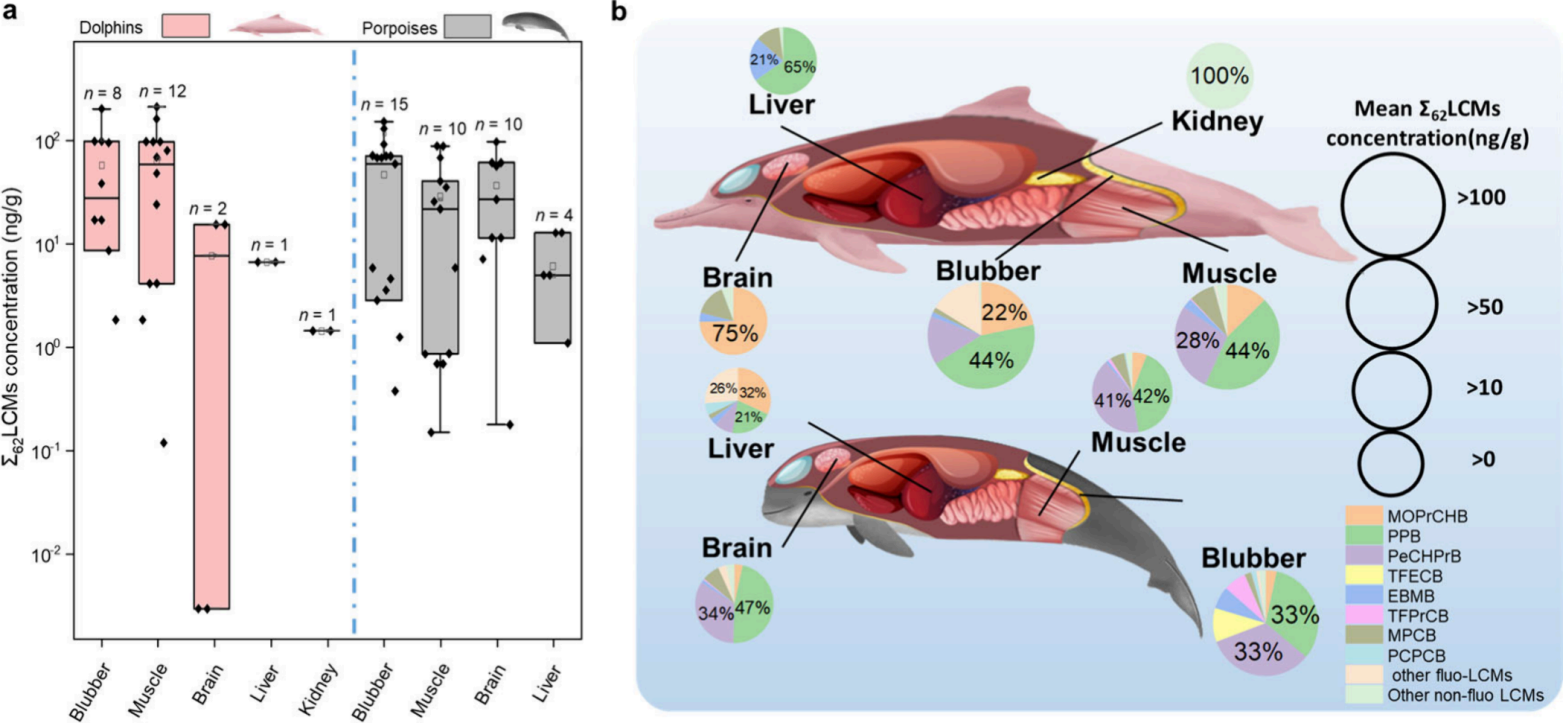
Distribution of liquid crystal monomers (LCMs) in tissues of marine cetaceans. (a) The composition profile of LCMs in tissues. (Summary statistics are provided in Tables S10 and S11. Raw data are provided in Table S15, and a visualization of the raw data set is shown in Figure S4.) Box plots show the median (center line) and the 25th and 75th percentiles (box limits). (b) Concentration of ΣLCMs in tissues. The samples included blubber, brain, muscle, liver, and kidney of the Indo-Pacific humpback dolphins (*SC*) and finless porpoises (*NP*) collected from 2018 to 2021 in the South China Sea (SCS) region.

The composition of LCMs in cetacean tissues exhibited variations (Figure 1b). Four nonhalo LCMs including 1-methyl-4-(4-(4-propyl­cyclo­hexyl)­cyclo­hexyl)­benzene (MPCB), 1-(prop-1-enyl)-4-(4-propyl­cyclo­hexyl)­cyclo­hexane (PPB), 1-(*trans*-4-pentyl­cyclo­hexyl)-4-propyl­benzene (PeCHPrB), and 1-methoxy-4-(*trans*-4-propyl­cyclo­hexyl)­benzene (MOPrCHB) accounted for more than 80% of the ΣLCMs in all tissues. These compounds were also commonly detected in displays on televisions, computers, and smartphones.^46,47^ The predominant LCM found in dolphin blubber was PPB (45%), whereas that in the brain and muscle was MOPrCHB (75%) and PPB (44%), respectively. In porpoises, the predominant LCMs in blubber were PeCHPrB (30%) and PPB (30%), whereas those in the brain were PPB (47%) and PeCHPrB (34%); those in muscle were PPB (42%) and PeCHPrB (41%). A significantly positive correlation was observed between concentrations of LCM in blubber and muscle of both dolphins and porpoises (Pearson *r* = 0.77, *p* < 0.01 for blubber samples; Pearson *r* = 0.96, *p* < 0.01 for muscle samples). Similar diet and metabolism of dolphins and porpoises likely contributed to the similarities in exposure patterns.^48,49^ However, such a pattern was not observed in brain tissues, where MOPrCHB and PPB dominated in the brains of dolphins and porpoises, respectively. Elucidating the underlying mechanism for this disparity is challenging due to the complexity of mass transfer of LCMs into the central nervous system. Nevertheless, several nonmutually exclusive factors may contribute, including (i) species-specific toxicokinetics (e.g., hepatic metabolism/biotransformation rates and elimination), (ii) differences in blood–brain barrier transport/partitioning driven by compound physicochemical properties, and (iii) dietary differences that alter the relative exposure to individual LCMs (and their metabolites) in prey. Given the limited brain sample size in this study, these hypotheses warrant targeted validation in future work using additional individuals and paired prey/biota measurements.^44,49,50^

The differences in the physiochemical and toxicokinetic properties of nonhalo LCMs and fluorinated/brominated LCMs could be important contributing factors.^4^ In this study, fluorinated LCMs accounted for 16% and 26% of the total LCMs detected in blubber and liver (Figure 1b), respectively, indicating pronounced enrichment in lipid-rich tissues. This pattern is consistent with the high lipophilicity of fluorinated LCMs. For example, EDFPPB contains fluorine atoms substituted on SP2C–H (connected to an aromatic ring), which can substantially increase hydrophobicity^51^ and therefore promote its accumulation in lipid-rich matrices. Previous animal studies have reported similar trends, supporting that the position of fluorine substitution is a key determinant of tissue partitioning and accumulation of LCMs in lipid-rich tissues.^23^ In contrast, fluorinated LCMs were rarely detected in other tissues with lower lipid content, further reinforcing that the lipid is an important sink for these compounds. These results collectively suggest that tissue enrichment of LCMs is not solely driven by their overall hydrophobicity; for fluorinated LCMs in particular, the position of fluorine substitution represents an additional important factor shaping their lipid partitioning.

### Comparison of LCM Concentrations with Other Matrices and Pollutants

Previous studies conducted by our group have reported LCMs in various matrices in the PRE and SCS regions since 2021 (Figure 2a). LCMs were widely detected in ambient air in Hong Kong with a maximum value of 13,500 pg/m^3^.^52^ LCM concentrations of up to 448 ppb were found in indoor dust from Hong Kong.^53^ Studies have reported that waste LCD panels can leach significant amounts of LCMs into the environment.^30^ LCMs were found in marine sediment and organisms in the SCS area.^7,54^ The LCMs exhibit model-predicted half-lives in water (ranging from 15.0 to 180 days) and model-predicted high *n*-octanol–water partition coefficient (log *K*_ow_) values (ranging from 4.3 to 13.2), indicating their persistence and potential for bioaccumulation in aquatic ecosystems (Table S1). Although some LCMs show relatively short aqueous half-lives, this does not necessarily reflect their fate in the whole ecosystem. A plausible explanation is that hydrophobic organics can rapidly partition from water into biota, particles, or organic matter phases where degradation can be slower; however, the quantitative contribution of these processes remains uncertain and requires further investigation.

**Figure 2:**
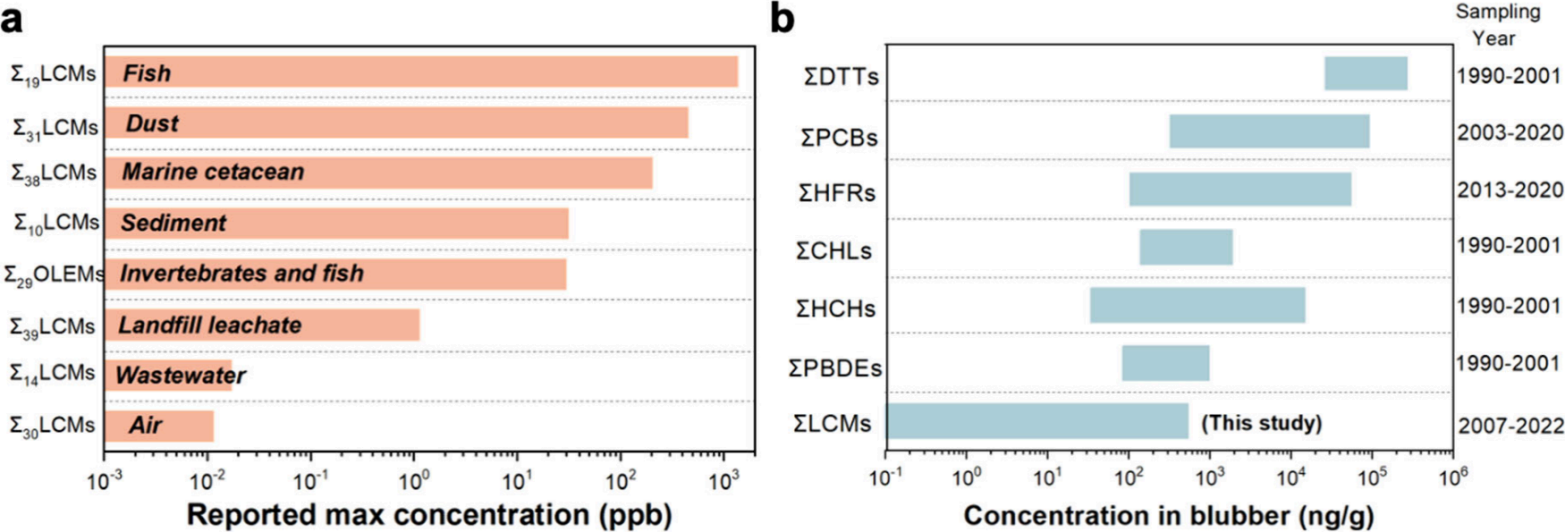
Comparisons of LCM concentrations with other matrices and pollutants. (a) Comparison of ΣLCMs concentrations in different matrices; all values represent reported maximum ΣLCMs levels (converted to ppb for cross-matrix comparison in Text S8.^7,12,20,30,52–54^ (b) Comparison of ΣLCMs concentrations against other groups of organic contaminants in marine cetaceans in blubber collected from the South China Sea (SCS), including DDTs (dichloro­diphenyl­tri­chloro­ethanes), PCBs (polychlorinated biphenyls), HFRs (halogenated flame retardants), CHLs (chlorinated hydrocarbons), HCHs (hexa­chloro­cyclo­hexanes), and PBDEs (polybrominated diphenyl ethers).^56–59^

Among our priority LCMs, 1-[(*trans*,*trans*)-4′-ethenyl­[1,1′-bicyclo­hexyl]-4-yl]-4-methyl­benzene (EBMB) and MPCB were also detected in surface sediments from the northern SCS, noting that the sediment study targeted/detected a limited set of 10 LCMs.^7^ LCMs have also been detected in scallops, whelks, shrimps, crabs, and fishes from the SCS region.^12^ These prey species are known to be part of the natural diet of dolphins and porpoises.^55^ For example, in 13 fish species collected across the PRE, muscle tissue burdens spanned 2.93–606 ng/g dw (median: 37.4 ng/g dw), and detection frequencies for most analytes exceeded 50%, indicating that exposure levels in lower-trophic organisms can reach environmentally and toxicologically relevant concentrations.^54^ Together, these findings indicate that LCMs occur in potential prey organisms in the region, supporting dietary exposure as a plausible route for the top predators. Hence, the exposure doses used in our cell experiments (Table S9) are within the same order of magnitude as LCM burdens reported in prey organisms,^54^ supporting environmental relevance. Given that coastal residents consume seafood that overlaps with the diet of dolphins and porpoises, it is reasonable to suspect that humans may also be exposed to LCMs through seafood consumption; however, human internal exposure and accumulation remain to be investigated. Therefore, further investigations are warranted to elucidate the processes of the uptake, accumulation, and metabolism of these emerging contaminants from dietary sources.

ΣLCMs concentrations measured in this study were compared with the previously reported blubber concentration of legacy and emerging organic contaminants in marine cetaceans from the SCS (Figure 2b). The concentrations of ΣLCMs were substantially lower than those of legacy pollutants such as dichloro­diphenyl­tri­chloro­ethanes (DDTs), polychlorinated biphenyls (PCBs), halogenated flame retardants (HFRs), chlorinated hydrocarbons (CHLs), and hexa­chloro­cyclo­hexanes (HCHs) (*p* < 0.01). This difference likely reflects a combination of factors: LCMs are emerging contaminants with lower historical production and environmental release than legacy pollutants such as DDTs and PCBs. Additionally, the localized release of LCMs from e-waste recycling and discarded electronic devices may lead to elevated exposure in marine organisms. Considering the increasing production, usage, and obsolescence of LCD devices, as well as the growing load of e-waste, LCMs emission and their levels in various environmental matrices are expected to continuously rise.^2,3^

Moreover, the environmental threat posed by LCMs is likely compounded by their coexposure to other pollutants, both legacy and emerging, in real-world ecosystems. LCMs are often detected alongside PFASs, heavy metals, and microplastics, which raises the possibility of additive or synergistic toxic effects.^31,21^ In cetaceans, such coexposures could potentially influence immune function, reproduction, or neurodevelopment, although direct evidence for these combined effects is currently lacking. This underscores the need for exposome-level studies that evaluate cumulative toxicity and the long-term impacts of such pollutants on wildlife health and human populations. The potential for LCMs to interact with other contaminants may amplify their neurotoxic and endocrine-disrupting effects, with serious implications for marine mammal populations already under pressure from climate change, habitat loss, and overfishing.

### Temporal Trends and Determinants of LCM Concentrations in Porpoise Blubber

Concentrations of PCPCB increased over time, whereas those of 1-ethoxy-4-(*trans*-4-propyl­cyclo­hexyl)­benzene (EOPrCHB) and 1-ethyl-4-(4-(4-propyl­cyclo­hexyl)­phenyl)­benzene (EPB) decreased significantly (*p* < 0.05). For other individual LCMs or ΣLCMs, no significant temporal trend was found for their concentrations of adult porpoise blubber collected from 2007 to 2021 (Figure 3a, *p* > 0.05). Temporal patterns of LCMs in porpoise blubber samples also align with global LCD production trends, paralleling trajectories observed for other pollutants such as PBDEs and PFASs.^40,41^ The mean ΣLCMs concentration was significantly greater in 2007–2017 than in 2018–2021 (Figure 3b, *p* < 0.01), showing an increase from 2007 to 2015, a steady phase in 2015–2017, and a decline from 2017 to 2021 (Figure 3c, *p* < 0.05). The observed temporal trend of ΣLCMs concentration likely reflects the evolution changes in display technology over time, particularly the gradual replacement of LCDs by light-emitting diode (LED) displays in the Asia-Pacific region since 2015.^50,60,61^ Despite this shift, the widespread use and disposal of existing LCD devices continue to release LCMs into the environment, posing ongoing ecological and health risks.

**Figure 3:**
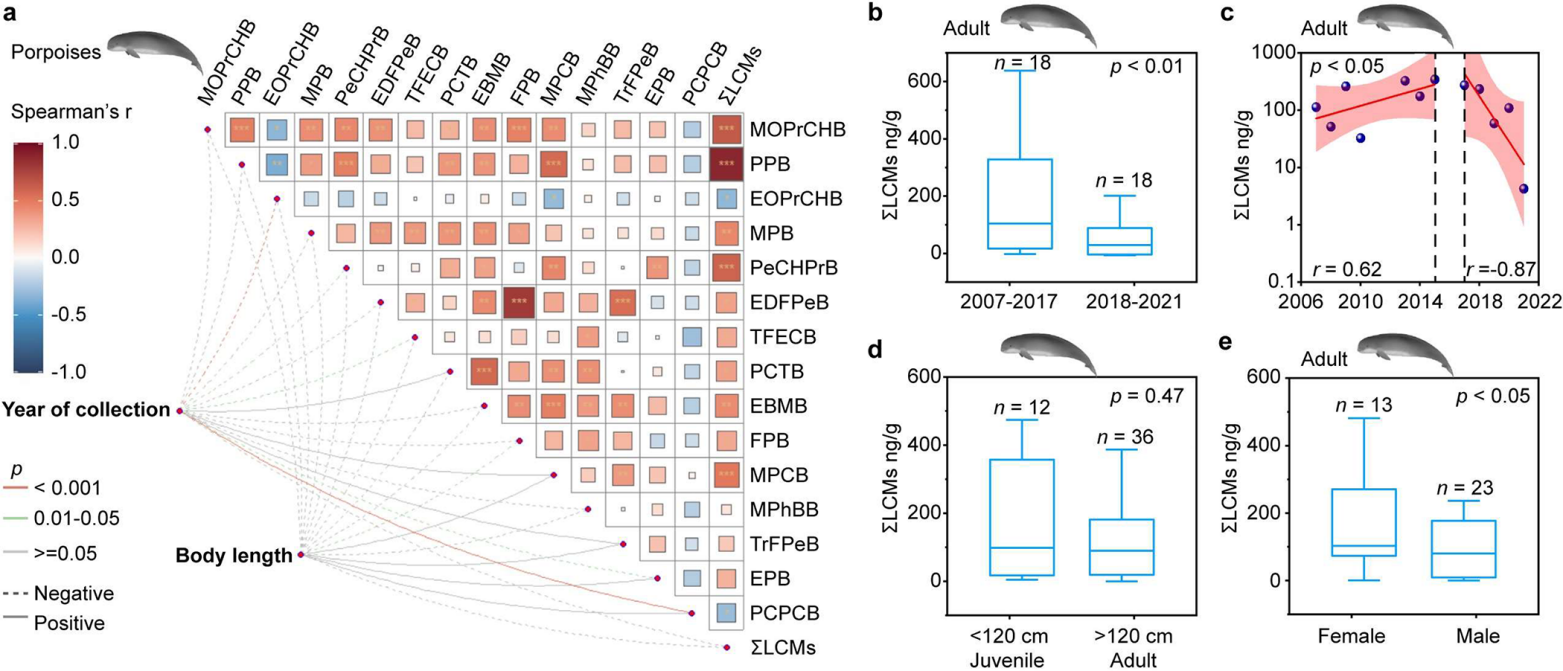
Temporal trends and determinants of LCM concentrations in cetaceans (unit: ng/g lipid weight). (a) Heat map showing the Spearman correlation coefficients between each LCM and ΣLCMs (*p < 0.05; **p < 0.01; ***p < 0.001). (b) The comparison of ΣLCMs concentrations between two periods (2007–2017 and 2018–2021); each year included in the comparison contains ≥3 individual samples. (c) Scatter plot showing the correlation between sampling year and ΣLCMs concentration, where the red line is the linear fitting and the pink area represents 95% confidence intervals. The dots represent the yearly median concentrations of ΣLCMs, with each dot calculated from at least three individual samples. (d) Comparison of ΣLCMs concentrations in adults (body length > 120 cm) and calves (body length < 120 cm). (e) Comparison of ΣLCMs concentrations in female and males; the samples with body length > 120 cm were used to minimize the possible age-related differences. Box plots show the median (center line) and the 25th and 75th percentiles (box limits). LCM concentrations in blubber are expressed on a lipid weight in this section to minimize variability caused by differences in lipid content across samples. Details of these blubber samples of LCM concentration (*n* = 48), sampling year, and biological factors from 2007 to 2021 are provided in Tables S3 and S16.

Previous studies have shown that sex and age can influence contaminant burdens in marine mammals.^50,61^ For marine cetaceans, body length is used as a proxy for age, with older individuals generally exhibiting higher accumulation. However, the concentrations of ΣLCMs were not affected by body length for either sex (Figure 3a,d; *p* ≫ 0.05). For marine mammals, adult females often exhibit lower concentrations of contaminants than males, which has been attributed to both lactational transfer to offspring and, in some cases, placental transfer.^62^ In contrast, our findings showed that female porpoises had significantly higher concentrations of ΣLCMs in blubber compared to males (Figure 3e; *p* < 0.05). These discrepancies in ΣLCMs burden between sexes suggest complex toxicokinetics of LCMs, warranting further investigation.

The rapid advancement of display technology has led to the swift obsolescence of outdated LCM substances and the adoption of new LCMs in LCD manufacturing.^63^ In this study, authentic standards were obtained for 62 LCMs as target analytes based on information from open research articles and manufactory documentations. However, the list might not be comprehensive, as some new LCMs currently in use are not disclosed due to commercial confidentiality. It is also possible that some of the 62 target LCMs were used in specific models of LCD devices that may have been discontinued. These factors likely complicate the trend analysis of ΣLCMs levels in marine cetaceans.

Additionally, different LCM compounds may exhibit differential toxicokinetic processes (i.e., absorption, distribution, metabolism, and excretion) in exposed animals depending on their physicochemical properties.^23,24^ LCMs with high hydrophobicity and persistency are generally considered to be more bioaccumulative. However, the main LCMs detected in porpoise blubber in this study (i.e., EBMB, PeCHPrB, and MPCB) had relatively shorter modeled half-lives in water (37.5 days) and relatively lower log *K*_ow_ (6.26–8.72) compared with other LCMs, some of which have predicted log *K*_ow_ values up to 13.17 (Table S1). Therefore, within the broader LCM chemical space, these compounds may be more readily metabolized and excreted, which could contribute to a less pronounced long-term accumulation.

### Source Apportionment of LCMs in Marine Cetaceans

The sources of LCMs in cetacean tissues were examined by use of a positive matrix factorization–multiple linear regression (PMF-MLR) model, with the results demonstrating a strong correlation between the determined profiles in cetaceans and the documented profiles in electronic devices (*r* = 0.96, Pearson *p* < 0.05, Figure S6). The four-factor solution was determined as the best fit under the control of three error estimation methods (Table S13). The Factor 1 profile was related to computer LCD screens of Brand A from a Taiwanese manufacturer, characterized by an approximately 1:1:1 ratio of PPB, PeCHPrB, and MPCB.^47^ Factor 2 exhibited significant loading of EBMB (60%) and PPB (40%), a fingerprint of the computer LCD screen of Brand P.^64^ The Factor 3 profile was associated with television LCD screens, as indicated by the presence of PPB and PeCHPrB.^47^ The Factor 4 profile was linked to smartphone screens, as evidenced by the dominance of PPB followed by EDFPPB (Figure 4a).^46^ The representative brands in the PMF analysis were selected because their LCM profiles had been well characterized in previous studies,^46,47^ providing reliable source fingerprints for comparison. Although these brands cover most representative products on the market, they cannot account for all possible electronic devices.

**Figure 4:**
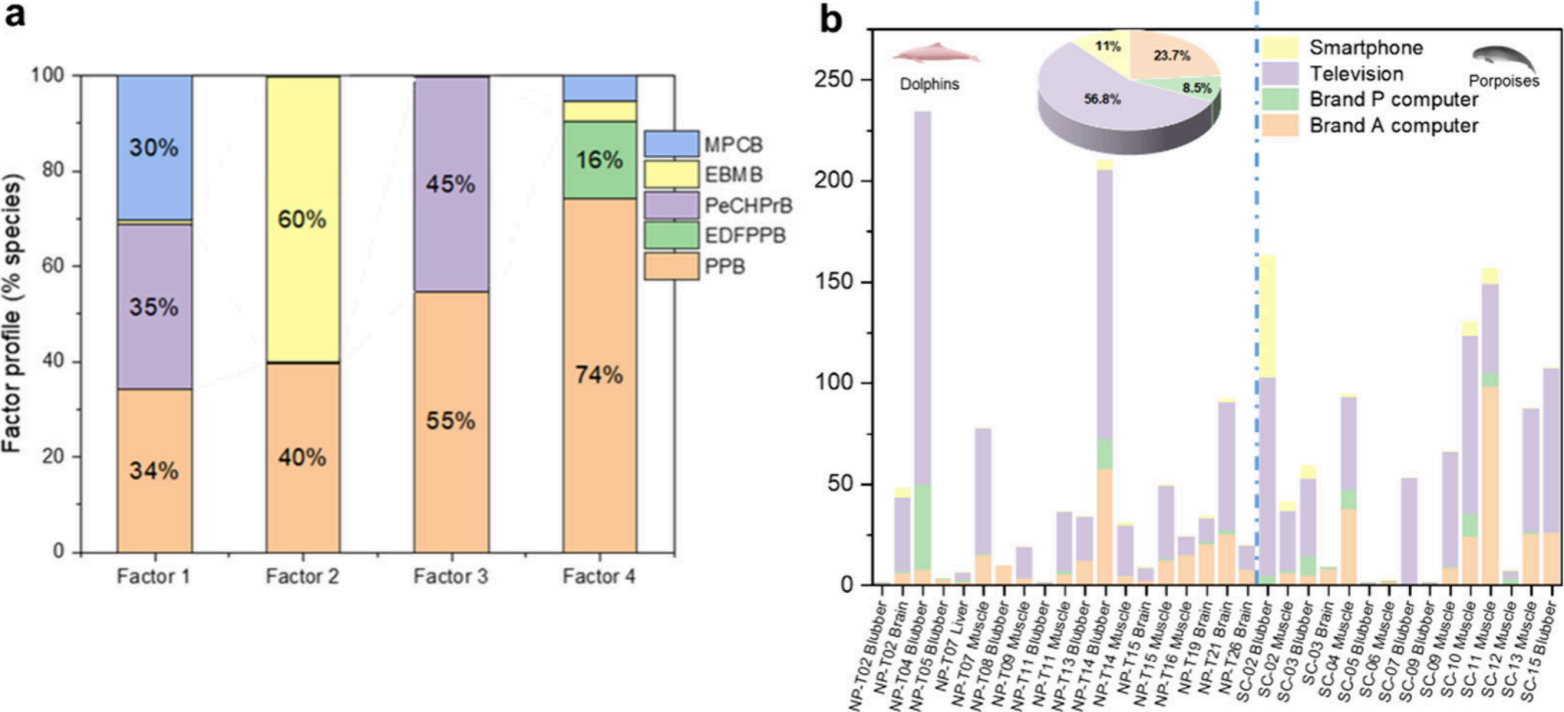
Source apportionment of LCMs in cetaceans. (a) Profiles for the four factors that were obtained by the positive matrix factorization–multiple linear regression (PMF-MLR) model. Only strong species are shown (EPA PMF S/N-based categorization); weak species were included with reduced weight. (b) Contributions of the four PMF factors to the ΣLCMs measured in each sample tissue.

The contributions of the four PMF factors to the ΣLCMs concentration measured in each sample are shown in Figure 4b. Although the source contributions to ΣLCMs in each sample varied widely depending on tissues and cetacean species (Figure 4b), the overall pattern suggested that the presence of LCMs in dolphins is primarily attributed to television and computer displays. The mean percentage contributions for the four factors were 56.8% from televisions, 23.7% from Brand A computer LCD panels, 11.0% from smartphones, and 8.5% from Brand P computer LCD panels (Figure 4b), which are consistent with LCM composition/source profiles reported for LCD device screen materials (e.g., waste smartphone screens and components).^46^ However, it is important to note that these results reflect only the sources analyzed in this study and might not account for all potential sources of LCMs. These results suggest that marine mammals mainly receive LCMs from large screen televisions, but emissions from computers and smartphones (either as e-waste or during normal use) cannot be ignored.

### Transcriptomics of Cetacean Cells Following LCM Exposure

Eight LCMs with notable concentrations in dolphins and porpoises were selected for toxicity assessment using Melon-Head Skin Fibroblast (MHSF) and Melon-Head Kidney Fibroblast (MHKF) primary cell cultures. Cell viability results showed that PeCHPrB, EBMB, 4-(4-methyl­phenyl)-4′-(3-butenyl)­bicyclo­hexyl (MPhBB), 4-propyl-4′-[4-(tri­fluoro­methoxy)­phenyl]-1,1′-bicyclo­hexyl (PCTB), MPCB, and PPB had no significant effect, whereas MOPrCHB and EDFPPB reduced cell viability at greater concentrations (258 ng/mL for MOPrCHB and 51 ng/mL for EDFPPB) (Tables S8, S9, and Figure S7).^65,66^

All eight LCMs induced changes in gene expression in MHKF cells (Figure 5a). In contrast, among MHSF cells, exposure to MOPrCHB led to a significantly higher number of differentially expressed genes (DEGs) compared to the other LCMs (*p* < 0.05, |FC| ≥ 2) (Figure 5b). In MHKF cells, PeCHPrB upregulated genes related to circulatory and signaling processes (Figure 5c) but downregulated genes involved in tissue and organ development (Figure 5d). This is consistent with a recent mechanistic toxicology study showing that selected LCMs significantly antagonized PPARγ in reporter-gene assays (supported by *in silico* docking/virtual screening) and induced transcriptomic and metabolomic dysregulation in human kidney (HK2) cells,^65^ as well as preferentially distributed to lipophilic tissues (e.g., adipose and liver) in mice, suggesting their potential for *in vivo* tissue retention/accumulation and long-term persistence.^23^ MOPrCHB and EDFPPB suppressed genes linked to cell proliferation, vasculature, and the extracellular matrix. PPB and EBMB also affected vasculature-related genes. In MHSF cells, MOPrCHB upregulated genes linked to inflammation and morphogenesis (Figure 5e) but downregulated those related to DNA replication and cell cycle, suppressing proliferation (Figure 5f). This transcriptomic suppression of DNA replication and cell cycle functions is consistent with the reduction in cell viability observed for MOPrCHB at Concentration 2 (Table S9). EDFPPB affected sensory organ development, while EBMB and PPB reduced skeletal system gene expression. Inhibition of cell proliferation or induction of apoptosis is a common cellular response to stress.^67^ Notably, LCMs with higher predicted reactivity or PB classification (Table S8), such as MOPrCHB and EDFPPB, corresponded to more pronounced transcriptional changes in DEGs related to oxidative stress, DNA damage response, and cell cycle regulation in both MHSF and MHKF cells. A key limitation of the transcriptomic analysis is that no biological replicates (i.e., cells derived from multiple individuals) were available due to the limited availability of cetacean primary cells. Therefore, the RNA-seq results should be interpreted as responses from this particular individual/cell preparation and may not fully represent population-level variability. Future work will address this by establishing cryopreserved primary cell banks from multiple stranded individuals and including biological replicates to improve the generalizability of the transcriptomic responses. To substantiate the observed transcriptomic alterations and potential health risks, follow-up toxicological investigations using standard vertebrate models, such as fish or rodents, are recommended. The details of transcriptomic analysis in exposed MHSF and MHKF cells are shown in Figures S8 and S9.

**Figure 5:**
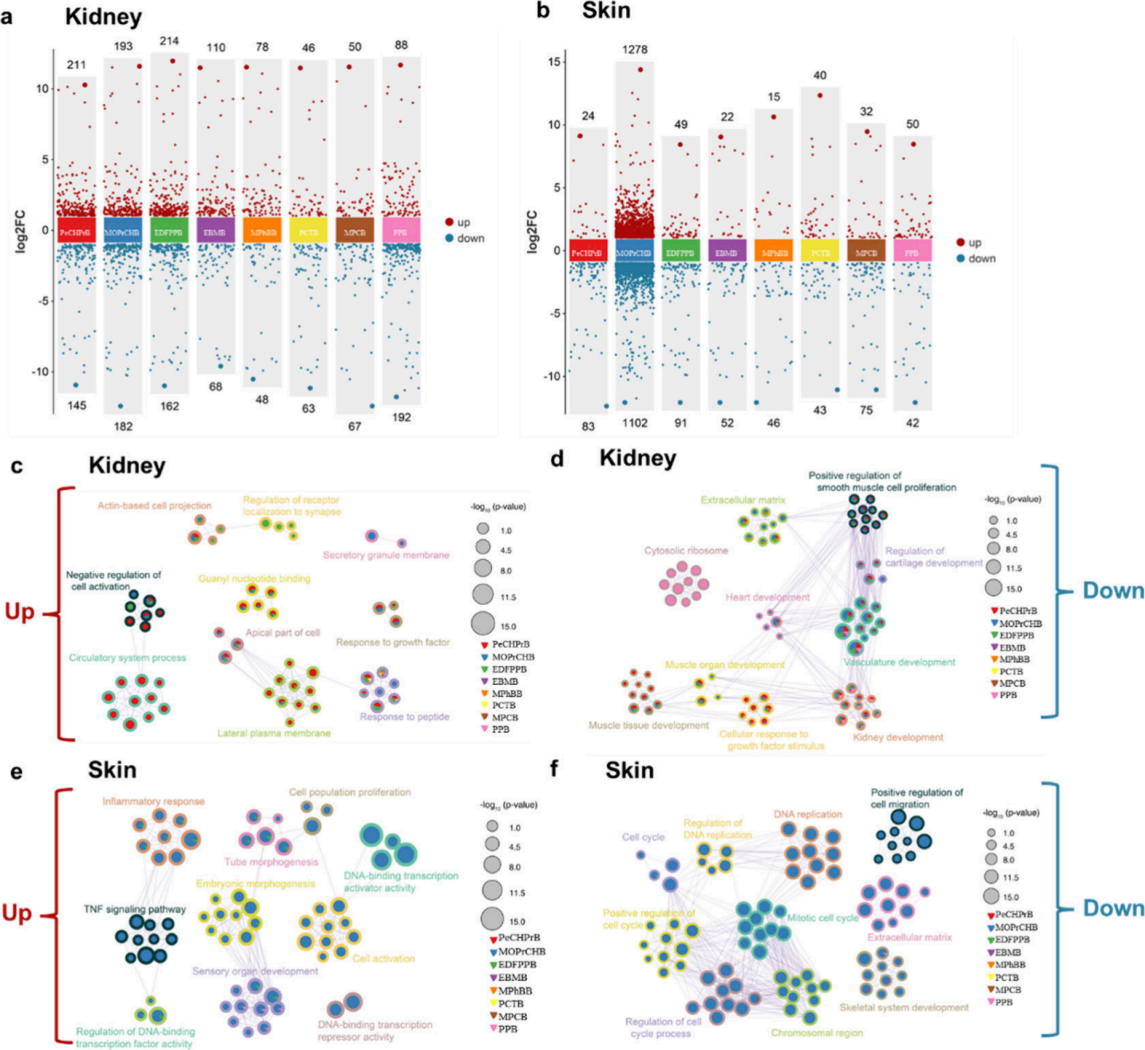
Transcriptomic analysis using cetacean cells exposed to LCMs (Concentration 2 in Table S9). (a, b) Volcano plots of gene expression of (a) MHKF and (b) MHSF cells exposed to LCMs. (c–f) Representative significantly enriched functional terms (subset shown for clarity) derived from DEG sets (Metascape) for MHKF cells ((c) upregulated terms and (d) downregulated terms) and MHSF cells ((e) upregulated terms and (f) downregulated terms).

### Quantitative PCR Analysis

Quantitative PCR (qPCR) was performed to confirm gene expressions, using primers designed from the RNA-seq transcript sequences (Table S14). The qPCR validation was conducted using RNA extracted from the higher LCM exposure concentration (i.e., Concentration 2 in Table S9), which was selected because transcriptomic responses were more pronounced at this dose. Similar patterns of expression were observed among both RNA sequencing and qPCR analyses (Figures S10 and S11). For instance, exposure to MOPrCHB significantly decreased the relative mRNA abundances of *CDK1*, *CDK2*, *CDK6*, *CCND1*, *CCNA2*, *MCM3*, *MCM4*, and *PCNA* in MHSF cells. These results are consistent with the changes in gene expression estimated from Fragments Per Kilobase of transcript per Million mapped reads (FPKM). Further comparisons between RNA sequencing and qPCR results are detailed in Text S9 and Figures S10 and S11.

### Environmental Implications

Our findings highlight the urgent need to recognize LCMs as emerging contaminants of concern in marine ecosystems. LCMs are capable of bioaccumulating in multiple tissues, including brain tissue, and may exert neurotoxic effects through mechanisms analogous to known contaminants such as PCBs, PBDEs, and PFASs, including disruption of calcium signaling, oxidative stress, interference with thyroid hormone homeostasis, and altered neuronal differentiation.^68–70^ Although the published *in vivo* evidence comes from a different FLCM (not among the compounds detected or tested in this study), that work indicates that LCM-type chemicals may have the potential to perturb lipid metabolic regulation such as PPARα-mediated fatty acid oxidation.^28^ These observations indicate that LCMs may have the potential to affect multiple organ systems, and similar transcriptional alterations could possibly occur in brain tissue upon penetration of the blood–brain barrier. The ability of LCMs to cross this barrier may be related to their physicochemical properties, such as moderate to high hydrophobicity and relatively low molecular weights,^24^ which facilitate passive diffusion. Additionally, their aromatic and halogenated structures might enhance membrane permeability, similar to other lipophilic contaminants known to accumulate in neural tissues.^68–70^ To substantiate this possibility, follow-up toxicological investigations using standard vertebrate models such as mice and zebrafish are urgently needed.

From a policy and management perspective, LCMs should be recognized as emerging contaminants of concern, and regulatory frameworks should prioritize the identification and restriction of the most bioaccumulative and toxic compounds while encouraging the development and evaluation of safer-by-design alternatives. Although MPCB was among the most abundant LCMs detected in our samples, it showed comparatively weaker cytotoxic and transcriptional responses than some other priority LCMs (e.g., MOPrCHB and PeCHPrB) under our tested conditions. However, because the tested concentrations were not identical across all LCMs and prior studies have also reported biological effects of MPCB,^7^ we do not propose MPCB as a “safe” replacement based on the present data set alone. Instead, our results highlight the need for standardized head-to-head comparisons (same dose range) and broader hazard and fate assessments when evaluating candidate alternatives for display technologies. The Pearl River Estuary, a major hub for LCD production and e-waste recycling, presents a unique opportunity to implement circular economy strategies that minimize the release of harmful LCMs. Achieving these goals will require multistakeholder collaboration, informed policymaking, and investment in greener chemical alternatives, ultimately protecting both ecological and human health while promoting environmental sustainability. This integrated perspective links the molecular-level toxicological potential of LCMs with their ecosystem-level and societal implications, highlighting the urgent need for monitoring, regulation, and proactive mitigation strategies to safeguard marine biodiversity and human populations dependent on marine resources.

## Supplementary Material





## Data Availability

Data on detailed GC-MS/MS analysis of LCMs, RNA sequencing, quantitative PCR, and toxic potency evaluation that support the findings of this study are available from the corresponding authors upon reasonable request. All other data supporting the findings of this study are available within the paper and its Supporting Information.

## Notes

Descriptions of methods including the marine cetacean species involved in this study, suspect screening, LCM detection, positive matrix factorization–multiple linear regression, alteration of gene expression related to cell cycle, and establishment of immortal cetacean fibroblast cells; details of the sampling, mass spectrum, and toxicological analysis methods, as well as the concentration of LCMs in different samples; results and quality assurance analytical measurements of LCMs in tissues of dolphins and porpoises; and supporting data of cytotoxicity and qPCR on LCM toxicity derived from cetacean cell exposure (PDF)
Tables S15 and S16: the concentration of individual LCMs in marine cetacean tissue samples collected from the studied area and the concentration of individual LCMs in blubber tissue samples for temporal trend analysis (XLSX)

## References

[1] Schadt M. (1997). Liquid crystal
materials and liquid crystal displays. Annu.
Rev. Mater. Sci..

[2] Jones J. C. (2018). The fiftieth
anniversary of the liquid crystal display. Liquid
Crystals Today.

[3] Forti, V. ; Balde, C. P. ; Kuehr, R. ; Bel, G. The Global E-waste Monitor 2020: Quantities, flows and the circular economy potential; United Nations University/United Nations Institute for Training and Research, 2020.

[4] Su H., Shi S., Zhu M., Crump D., Letcher R. J., Giesy J. P., Su G. (2019). Persistent, bioaccumulative, and toxic properties of liquid crystal
monomers and their detection in indoor residential dust. Proc. Natl. Acad. Sci. U.S.A..

[5] Wang Y., Jin Q., Lin H., Xu X., Leung K. M., Kannan K., He Y. (2024). A review of liquid
crystal monomers (LCMs) as emerging contaminants:
Environmental occurrences, emissions, exposure routes and toxicity. Journal of Hazardous Materials.

[6] Su H., Shi S., Zhu M., Li J., Su G. (2021). Liquid crystal
monomers
(LCMs) in sediments: method validation and detection in sediment samples
from three typical areas. Environ. Sci. Technol..

[7] Tao D., Jin Q., Ruan Y., Zhang K., Jin L., Zhan Y., Su G., Wu J., Leung K. M., Lam P. K. (2022). Widespread
occurrence of emerging E-waste contaminants-Liquid crystal monomers
in sediments of the Pearl River Estuary, China. Journal of Hazardous Materials.

[8] Ge J., Du B., Shen M., Feng Z., Zeng L. (2023). A review of liquid
crystal monomers: Environmental occurrence, degradation, toxicity,
and human exposure of an emerging class of E-waste pollutants. Environ. Pollut..

[9] Dubocq F., Kärrman A., Gustavsson J., Wang T. (2021). Comprehensive chemical
characterization of indoor dust by target, suspect screening and nontarget
analysis using LC-HRMS and GC-HRMS. Environ.
Pollut..

[10] Liu Q., Abbatt J. P. (2021). Liquid crystal display
screens as a source for indoor
volatile organic compounds. Proc. Natl. Acad.
Sci. U.S.A..

[11] Miramontes
Gonzalez P., Li L. (2024). Evaluating the Environmental Persistence
of Liquid Crystal Monomers Indoors and Outdoors. Environmental Science & Technology Letters.

[12] Wang J., Nan J., Li M., Yuan G., Zhao Y., Dai J., Zhang K. (2022). First evidence
of contamination in aquatic organisms with organic
light-emitting materials. Environmental Science
& Technology Letters.

[13] Li Y., Zhang T., Cheng Z., Zhang Q., Yang M., Zhao L., Zhang S., Lu Y., Sun H., Wang L. (2022). Direct evidence on occurrence of
emerging liquid crystal monomers
in human serum from E-waste dismantling workers: Implication for intake
assessment. Environ. Int..

[14] Yang R., Wang X., Niu Y., Chen X., Shao B. (2023). Fluorinated
liquid-crystal monomers in paired breast milk and indoor dust: A pilot
prospective study. Environ. Int..

[15] Zhu W., Shi H., Huang B., Zhong K., Huang Y. (2021). Geology and geochemistry
of large gas fields in the deepwater areas, continental margin basins
of northern South China Sea. Marine and Petroleum
Geology.

[16] Zou M.-Y., Ran Y., Gong J., Mai B.-X., Zeng E. y. (2007). Polybrominated diphenyl
ethers in watershed soils of the Pearl River Delta, China: occurrence,
inventory, and fate. Environ. Sci. Technol..

[17] Zhang B.-Z., Guan Y.-F., Li S.-M., Zeng E. Y. (2009). Occurrence of polybrominated
diphenyl ethers in air and precipitation of the Pearl River Delta,
South China: annual washout ratios and depositional rates. Environ. Sci. Technol..

[18] Ruan Y., Zhang K., Wu C., Wu R., Lam P. K. (2019). A preliminary
screening of HBCD enantiomers transported by microplastics in wastewater
treatment plants. Sci. Total Environ..

[19] Wang, W.-X. ; Rainbow, P. S. Environmental Pollution of the Pearl River Estuary, China; Springer, 2020.

[20] Zhan Y., Jin Q., Lin H., Tao D., Law L. Y., Sun J., He Y. (2023). Occurrence, behavior
and fate of liquid crystal monomers in municipal
wastewater. Water Res..

[21] Wang Q., Ruan Y., Jin L., Zhang X., Li J., He Y., Wei S., Lam J. C., Lam P. K. (2021). Target,
nontarget,
and suspect screening and temporal trends of per-and polyfluoroalkyl
substances in marine mammals from the South China Sea. Environ. Sci. Technol..

[22] Zhu B., Lai N. L., Wai T.-C., Chan L. L., Lam J. C., Lam P. K. (2014). Changes of accumulation
profiles from PBDEs to brominated
and chlorinated alternatives in marine mammals from the South China
Sea. Environ. Int..

[23] Kong Y., Wen Y., Su G., Peng Y., Cui X. (2023). Tissue-specific uptake
and distribution of liquid crystal monomers (LCMs) in mice. Environ. Int..

[24] Zhu M., Su H., Bao Y., Li J., Su G. (2022). Experimental determination
of octanol-water partition coefficient (KOW) of 39 liquid crystal
monomers (LCMs) by use of the shake-flask method. Chemosphere.

[25] He S., He J., Ma S., Wei K., Wu F., Xu J., Jin X., Zhao Y., Martyniuk C. J. (2024). Liquid crystal monomers disrupt photoreceptor
patterning of zebrafish larvae via thyroid hormone signaling. Environ. Int..

[26] He S., He J., Wu F., Zhao Y., Jin X., Martyniuk C. J. (2024). In vivo
and in silico toxicity assessment of four common liquid crystal monomers
to Daphnia magna: Novel endocrine disrupting chemicals in crustaceans?. Science of The Total Environment.

[27] Wu J., Lv D., Lin W., Mao Y., Xia Y., Feng L., Zhao T., Mao X., Shu F., Guo H. (2025). Chronic exposure
to liquid crystal monomer EBCN at environmentally relevant concentrations
induces testicular dysfunction via the gut-testis axis. Journal of Hazardous Materials.

[28] Peng L., Qi Z., Xiang L., Wang W., Cao G., Ru Y., Wang X., Lin S., Yang Z., Yan H. (2025). Fluorinated liquid crystal monomer (FLCM) induces kidney
dysfunction
by disrupting PPARα-mediated fatty acid oxidation: In vivo,
in vitro, and in silico assays. Environmental
Chemistry and Ecotoxicology.

[29] Zhang Z., Yuan S., Yang Z., Liu Y., Liu S., Chen L., Wu B. (2025). Hepatotoxicity of Three Common Liquid
Crystal Monomers in Mus musculus: Differentiation of Actions Across
Different Receptors and Pathways. Environ. Sci.
Technol..

[30] Jin Q., Tao D., Lu Y., Sun J., Lam C. H., Su G., He Y. (2022). New insight on occurrence of liquid crystal monomers: A class of
emerging e-waste pollutants in municipal landfill leachate. Journal of Hazardous Materials.

[31] Sun Y., Zeng Y., Rajput I. R., Sanganyado E., Zheng R., Xie H., Li C., Tian Z., Huang Y., Yang L. (2022). Interspecies differences
in mammalian susceptibility to legacy POPs and trace metals using
skin fibroblast cells. Environ. Pollut..

[32] Krueger, F. ; James, F. ; Ewels, P. ; Afyounian, E. ; Weinstein, M. ; Schuster-Boeckler, B. ; Hulselmans, G. Trim Galore! (Version 0.6.10). Zenodo, 2023. https://zenodo.org/records/7598955.

[33] Dobin A., Davis C. A., Schlesinger F., Drenkow J., Zaleski C., Jha S., Batut P., Chaisson M., Gingeras T. R. (2013). STAR: ultrafast
universal RNA-seq aligner. Bioinformatics.

[34] Yu Z.-P., Liu X., Zhang B., Shan L., Seim I., Yang G., Xu S.-X. (2022). High-quality chromosome-level genome assembly of the melon-headed
whale (Peponocephala electra). Zoological Research.

[35] Li B., Dewey C. N. (2011). RSEM: accurate transcript quantification from RNA-Seq
data with or without a reference genome. BMC
Bioinformatics.

[36] Love M. I., Huber W., Anders S. (2014). Moderated
estimation of fold change
and dispersion for RNA-seq data with DESeq2. Genome Biology.

[37] Zhang, J. scRNAtoolVis: Useful Functions to Make Your scRNA-seq Plot More Cool. GitHub, 2022. https://github.com/junjunlab/scRNAtoolVis.

[38] Zhou Y., Zhou B., Pache L., Chang M., Khodabakhshi A. H., Tanaseichuk O., Benner C., Chanda S. K. (2019). Metascape provides
a biologist-oriented resource for the analysis of systems-level datasets. Nat. Commun..

[39] Ruan Y., Lam J. C., Zhang X., Lam P. K. (2018). Temporal
changes
and stereoisomeric compositions of 1, 2, 5, 6, 9, 10-hexabromocyclododecane
and 1, 2-dibromo-4-(1, 2-dibromoethyl) cyclohexane in marine mammals
from the South China Sea. Environ. Sci. Technol..

[40] Rotander A., Kärrman A., Bavel B. v., Polder A., Riget F., Auđunsson G. A., Vikingsson G., Gabrielsen G. W., Bloch D., Dam M. (2012). Increasing levels of long-chain perfluorocarboxylic
acids (PFCAs) in Arctic and North Atlantic marine mammals, 1984–2009. Chemosphere.

[41] García-Alvarez N., Martín V., Fernández A., Almunia J., Xuriach A., Arbelo M., Tejedor M., Boada L. D., Zumbado M., Luzardo O. P. (2014). Levels
and profiles of POPs (organochlorine pesticides,
PCBs, and PAHs) in free-ranging common bottlenose dolphins of the
Canary Islands, Spain. Sci. Total Environ..

[42] Andvik C., Haug T., Lyche J. L., Borgå K. (2023). Emerging and
legacy contaminants in common minke whale from the Barents sea. Environ. Pollut..

[43] Myhre O., Zimmer K. E., Hudecova A. M., Hansen K. E., Khezri A., Berntsen H. F., Berg V., Lyche J. L., Mandal S., Duale N. (2021). Maternal exposure to a human based mixture of persistent
organic pollutants (POPs) affect gene expression related to brain
function in mice offspring hippocampus. Chemosphere.

[44] Zhao Y., Li Y., Qin X., Lou Q., Qin Z. (2016). Accumulation of polybrominated
diphenyl ethers in the brain compared with the levels in other tissues
among different vertebrates from an e-waste recycling site. Environ. Pollut..

[45] Andersen H. R., Nielsen J. B., Grandjean P. (2000). Toxicologic
evidence of developmental
neurotoxicity of environmental chemicals. Toxicology.

[46] Jin Q., Yu J., Fan Y., Zhan Y., Tao D., Tang J., He Y. (2023). Release Behavior of Liquid Crystal
Monomers from Waste Smartphone
Screens: Occurrence, Distribution, and Mechanistic Modeling. Environ. Sci. Technol..

[47] Liang X., Xie R., Zhu C., Chen H., Shen M., Li Q., Du B., Luo D., Zeng L. (2021). Comprehensive identification of liquid
crystal monomers—biphenyls, cyanobiphenyls, fluorinated biphenyls,
and their analogues—in waste LCD panels and the first estimate
of their global release into the environment. Environ. Sci. Technol..

[48] Carwardine, M. Handbook of whales, dolphins and porpoises; Bloomsbury Publishing, 2019.

[49] Spitz J., Trites A. W., Becquet V., Brind’Amour A., Cherel Y., Galois R., Ridoux V. (2012). Cost of living dictates
what whales, dolphins and porpoises eat: the importance of prey quality
on predator foraging strategies. PloS One.

[50] Weijs L., Dirtu A. C., Das K., Gheorghe A., Reijnders P. J., Neels H., Blust R., Covaci A. (2009). Inter-species differences
for polychlorinated biphenyls and polybrominated diphenyl ethers in
marine top predators from the Southern North Sea: Part 1. Accumulation
patterns in harbour seals and harbour porpoises. Environ. Pollut..

[51] Begue J., Bonnet-Delpon D. (2008). Effects of fluorine substitution
on biological properties. Bioorganic and Medicinal
Chemistry of Fluorine.

[52] Lin H., Li X., Qin X., Cao Y., Ruan Y., Leung M. K., Leung K. M., Lam P. K., He Y. (2024). Particle size-dependent
and route-specific exposure to liquid crystal monomers in indoor air:
Implications for human health risk estimations. Sci. Total Environ..

[53] Jin Q., Fan Y., Lu Y., Zhan Y., Sun J., Tao D., He Y. (2023). Liquid crystal
monomers in ventilation and air conditioning dust:
Indoor characteristics, sources analysis and toxicity assessment. Environ. Int..

[54] Jiang Y.-Y., Zeng Y., Long L., Guo J., Lu R.-F., Chen P.-P., Pan Z.-J., Zhang Y.-T., Luo X.-J., Mai B.-X. (2024). First Report on the Trophic Transfer and Priority List
of Liquid Crystal Monomers in the Pearl River Estuary. Environ. Sci. Technol..

[55] Pauly D., Trites A. W., Capuli E., Christensen V. (1998). Diet composition
and trophic levels of marine mammals. ICES Journal
of Marine Science.

[56] Ramu K., Kajiwara N., Lam P. K., Jefferson T. A., Zhou K., Tanabe S. (2006). Temporal variation and biomagnification
of organohalogen compounds in finless porpoises (Neophocaena phocaenoides)
from the South China Sea. Environ. Pollut..

[57] Xie Z., Zhang X., Liu F., Xie Y., Sun B., Wu J., Wu Y. (2024). First determination
of elevated levels of plastic additives
in finless porpoises from the South China Sea. Journal of Hazardous Materials..

[58] Xie Q., Yu R.-Q., Yu R., Wang Z., Zhang X., Wu Y. (2021). Historic changes of polychlorinated biphenyls (PCBs) in juvenile
and adult cetaceans from the Pearl River estuary from 2003 to 2020. Sci. Total Environ..

[59] Wang Q., Ruan Y., Jin L., Kot B. C., Leung K. M. Y., Lam P. K. (2023). Temporal Trends and Suspect Screening of Halogenated
Flame Retardants and Their Metabolites in Blubbers of Cetaceans Stranded
in Hong Kong Waters during 2013–2020. Environ. Sci. Technol..

[60] Zissis, G. ; Bertoldi, P. ; Ribeiro Serrenho, T. Update on the Status of LED-Lighting world market since 2018; Publications Office of the European Union, Luxembourg, 2021.

[61] Metcalfe C., Koenig B., Metcalfe T., Paterson G., Sears R. (2004). Intra-and
inter-species differences in persistent organic contaminants in the
blubber of blue whales and humpback whales from the Gulf of St. Lawrence,
Canada. Marine Environmental Research.

[62] Wu Y., Shi J., Zheng G. J., Li P., Liang B., Chen T., Wu Y., Liu W. (2013). Evaluation of organochlorine contamination in Indo-Pacific
humpback dolphins (Sousa chinensis) from the Pearl River Estuary,
China. Sci. Total Environ..

[63] Zhao G., Wang G., Guan Y., Li H. (2011). Research and application
of a new rapid heat cycle molding with electric heating and coolant
cooling to improve the surface quality of large LCD TV panels. Polym. Adv. Technol..

[64] Moon H.-B., Kannan K., Choi M., Yu J., Choi H.-G., An Y.-R., Choi S.-G., Park J.-Y., Kim Z.-G. (2010). Chlorinated
and brominated contaminants including PCBs and PBDEs in minke whales
and common dolphins from Korean coastal waters. Journal of Hazardous Materials.

[65] Zhao H., Li C., Naik M. Y., Wu J., Cardilla A., Liu M., Zhao F., Snyder S. A., Xia Y., Su G. (2023). Liquid crystal monomer: a potential PPARγ
antagonist. Environ. Sci. Technol..

[66] Xie W., Ouyang R., Wang H., Li N., Zhou C. (2020). Synthesis
and cytotoxicity of novel elastomers based on cholesteric liquid crystals. Liq. Cryst..

[67] Huang W., Hickson L. J., Eirin A., Kirkland J. L., Lerman L. O. (2022). Cellular
senescence: the good, the bad and the unknown. Nature Reviews Nephrology.

[68] Wayman G. A., Yang D., Bose D. D., Lesiak A., Ledoux V., Bruun D., Pessah I. N., Lein P. J. (2012). PCB-95 promotes
dendritic growth via ryanodine receptor-dependent mechanisms. Environ. Health Perspect..

[69] Johansson N., Eriksson P., Viberg H. (2009). Neonatal exposure
to PFOS and PFOA
in mice results in changes in proteins which are important for neuronal
growth and synaptogenesis in the developing brain. Toxicol. Sci..

[70] Cao Y., Ng C. (2021). Absorption, distribution,
and toxicity of per-and polyfluoroalkyl
substances (PFAS) in the brain: a review. Environmental
Science: Processes & Impacts.

