# Supplementary material for: Liquid Crystal Monomers Released from LCD Displays Accumulate in Endangered Marine Cetaceans Triggering Health Concerns
Source: Environ Sci Technol. 2026 Feb 25;60(9):7437–48. doi: 10.1021/acs.est.5c17767 (PMC12980831; doi:10.1021/acs.est.5c17767)
Supplement: Supplementary file 2 [file es5c17767_si_002.pdf]

Supporting Information for

Liquid crystal monomers released from LCD displays accumulate in endangered marine cetaceans triggering health concerns

Danyang Tao [a], Chengzhang Li [b], Yajing Sun [b], Yuefei Ruan [a], Qianqian Jin [c], Jiaji Sun [c], Yichun Lu [c], Brian C.W. Kot [a], Paul K.S. Lam [a,d,e], Fengchang Wu [f], Jia He [g], John P. Giesy [h,i,j], Kurunthachalam Kannan [k], Bo Liang [b*], Wenhua Liu [b], Lin Zhang [c], Yunsong Mu[l], Kenneth M.Y. Leung [a,d], Yuhe He [c,d*]

[a] State Key Laboratory of Marine Environmental Health and Department of Chemistry, City University of Hong Kong, Kowloon, Hong Kong SAR, China

[b] Guangdong Provincial Key Laboratory of Marine Disaster Prediction and Prevention and Guangdong Provincial Key Laboratory of Marine Biotechnology, Institute of Marine Sciences, Shantou University, Shantou 515063, China

[c] School of Energy and Environment and State Key Laboratory of Marine Environmental Health, City University of Hong Kong, Kowloon, Hong Kong SAR, China

[d] Research Centre for the Oceans and Human Health, City University of Hong Kong Shenzhen Research Institute, Shenzhen 518057, China

[e] Department of Science, School of Science and Technology, Hong Kong Metropolitan University, Kowloon, Hong Kong SAR, China

[f] State Key Laboratory of Environmental Criteria and Risk Assessment, Chinese Research Academy of Environmental Sciences, Beijing 100012, China

[g] Beijing Key Laboratory of Urban Hydrological Cycle and Sponge City Technology, College of Water Sciences, Beijing Normal University, Beijing 100875, China

[h] Department of Veterinary Biomedical Sciences and Toxicology Centre, University of Saskatchewan, Saskatoon, SK S7N 5B3, Canada

[i] Department of Environmental Sciences, Baylor University, Waco, TX 76798-7266, USA

[j] Department of Integrative Biology, Michigan State University, East Lansing MI 48895, USA

[k] Wadsworth Center, New York State Department of Health, Albany, NY 12237, USA and Department of Environmental Health Sciences, State University of New York at Albany, Albany, NY 12237, USA

[l] School of Chemistry and Life Resources, Renmin University of China, Beijing 100872, China

*Corresponding Author
Bo Liang
Email address: liangbo@stu.edu.cn
Tel: +86 754-86503274
Fax: +86 754-82902767
Address: 243 Daxue Road, Shantou, Guangdong, China 515063

Yuhe He
Email address: henry.he@cityu.edu.hk
Tel: +852 3442-4370

Fax: +852 3442-0688
Address: G5703, Yeung Kin Man Academic Building, City University of Hong Kong, Tat Chee Ave, Kowloon, Hong Kong SAR, China

Contents

Text S1. Descriptions of marine cetaceans 6
Text S2. LCMs analysis in cell culture 7
Text S3. Suspect screening 8
Text S4. Establishment of primary cetacean skin and kidney fibroblast cultures 9
Text S5. Cell viability assay 11
Text S6. Method of quantitative PCR validation 12
Text S7. Positive matrix factorization-multiple linear regression (PMF-MLR) 13
Text S8. Unit harmonization for cross-matrix comparison (Figure 2a) 14
Text S9. Alteration of gene expression related to cell cycle 15
Table S1. Detailed information of LCM standards. 16
Table S2. Sample information for porpoises (*NP*) and dolphins (*SC*). 22
Table S3. Blubber samples used for temporal trend analysis. 24
Table S4. Retention times, quantification ions, specific fragment ions, and standard working curves for the LCMs. Calibration curves were constructed using 6–9 concentration points selected from 0.01, 0.05, 0.1, 0.5, 1, 5, 10, 50, and 100 μg/L. 26
Table S5. Spike recoveries of 62 LCMs assessed in blubber matrix (selected as the highest-lipid tissue to represent worst-case extraction efficiency). Low concentration spike = 10 μg/L; high concentration spike = 50 μg/L. 28
Table S6. Spike recoveries of 62 LCMs assessed in liver, muscle, kindey, brain matrix (spiked concentration = 10 μg/L) 30
Table S7. Spike recoveries of 62 LCMs assessed in cell culture matrix (spiked concentration = 10 μg/L) ... 32
Table S8. The predictive toxicity of eight priority LCMs using OECD QSAR Toolbox v4.4.1. 33
Table S9. The eight priority LCMs applied in the toxicity test. 35
Table S10. Concentrations and relative contributions of liquid crystal monomers (LCMs) in different tissues of *NP*. 36
Table S11. Concentrations and relative contributions of liquid crystal monomers (LCMs) in different tissues of *SC*. 40
Table S12. Formula and the retention time of the suspect liquid crystal monomers (LCMs) in marine cetacean samples. 43
Table S13. Error estimates with four-factor and five-factor solutions. 44
Table S14. The primers for the targeted genes were designed by using Primer3 with transcript data extracted from RNA sequencing results. 45
Fig. S1. The picture of two marine cetaceans: (a) Indo-Pacific humpback dolphins (*SC*) and (b) finless porpoises (*NP*) 46
Fig. S2. Column plot of 62 target analytes and 2 surrogate recoveries at the medium spiking level (10 ng/g) LCMs in samples. 47

Fig. S3. Principal component analysis (PCA) of transcriptomic profiles of Melon-Head Skin Fibroblast (MHSF) and Melon-Head Kidney Fibroblast (MHKF) cells exposed to LCMs. (a) MHSF cells exposed to low concentration of LCMs. MOPrCHB was excluded because exposure caused excessive cytotoxicity and RNA integrity failed QC for RNA-seq; (b) MHSF cells exposed to high concentration of LCMs; (c) MHKF cells exposed to low concentration of LCMs; (d) MHKF cells exposed to high concentration of LCM. 48

Fig. S4. Heatmap of concentrations for individual LCMs (ng/g dry weight for all tissues) in marine cetacean tissue samples collected from the studied area. 49

Fig. S5. The procedure of identification of new LCMs via suspect screening. 50

Fig. S6. Correlation of PMF modeled results with original measurements for LCMs detected from each sample. 51

Fig. S7. The potential impact of LCMs on cell viability of Melon-Head Fibroblast (MHSF) and Melon-Head Kidney Fibroblast (MHKF) cells. Cell Counting Kit-8 (CCK-8) assay was performed with series dilution of LCMs. Cell Counting Kit-8 (CCK-8) allows sensitive colorimetric assays for the determination of cell viability in cell proliferation and cytotoxicity assays. 52

Fig. S8. Transcriptomic profiles of Melon-Head Skin Fibroblast (MHSF) cells following exposure to the eight priority LCMs. "-low" corresponds to exposure concentration 1 in Table S9, and "-high" corresponds to exposure concentration 2 in Table S9. Heatmap colors represent gene-wise z-scores across samples (red = relatively higher expression; blue = relatively lower expression). Pathway annotations summarize representative enriched functions for each gene module/cluster. 53

Fig. S9. Transcriptomic profiles of Melon-Head Kidney Fibroblast (MHKF) cells following exposure to the eight priority LCMs. "-low" corresponds to exposure concentration 1 in Table S9, and "-high" corresponds to exposure concentration 2 in Table S9. Heatmap colors represent gene-wise z-scores across samples (red = relatively higher expression; blue = relatively lower expression). Pathway annotations summarize representative enriched functions for each gene module/cluster. 54

Fig. S10. Expression pattern of cell cycle related genes of Melon-Head Skin Fibroblast (MHSF) cells exposed to LCMs. CDK1: cyclin dependent kinase 1; CDK2: cyclin dependent kinase 2; CDK4: cyclin dependent kinase 4; CDK6: cyclin dependent kinase 6; CCND1: cyclin D1; CCNA2: cyclin A2; MCM3: Mini chromosome maintenance complex component 3; MCM4: Mini chromosome maintenance complex component 4; PCNA: Proliferating cell nuclear antigen; TGFB1: Transforming growth factor beta 1; Orange line and dot: relative repression level quantified by qPCR; Blue line and dot: Fragments Per Kilobase of transcript per Million mapped reads (FPKM of related genes; #: p<0.05, ##: p<0.01 when compared to solvent control group (Ethanol) by t-test with data from qPCR; *: p<0.05, **: p<0.01, ***: p<0.001, and ****: p<0.0001 when compared to solvent control group (DMSO) by one-way ANOVA followed by Dunnett multiple comparison with data from qPCR. 55

Fig. S11. Expression pattern of cell cycle related genes of Melon-Head Kidney Fibroblast (MHKF) cells exposed to LCMs. CDK1: cyclin dependent kinase 1; CDK2: cyclin dependent kinase 2; CDK4: cyclin dependent kinase 4; CDK6: cyclin dependent kinase 6; CCND1: cyclin D1; CCNA2: cyclin A2; MCM3: Mini chromosome maintenance complex component 3; MCM4: Mini chromosome maintenance complex component 4; PCNA: Proliferating cell nuclear antigen; TGFB1: Transforming growth factor beta 1; Orange line and dot: relative repression level quantified by qPCR; Blue line and dot: FPKM of related genes; #: p<0.05, ##: p<0.01 when compared to solvent control group (Ethanol) by t test with data from qPCR; *: p<0.05, **: p<0.01, ***: p<0.001, and ****: p<0.0001 when compared to solvent control group (DMSO) by one-way ANOVA followed by Dunnett multiple comparison with data from qPCR. 56

References 57

Text S1. Descriptions of marine cetaceans

Coastal development has emerged as a critical issue in Hong Kong, where the growing demand for space has driven extensive land reclamation. This has resulted in the expansion of artificial coastlines and destruction of natural habitats essential for marine megafauna. Consequently, the ecological characteristics and attributes vital for the survival of local cetaceans and their prey have been significantly altered. Over the past two decades, large sections of western Hong Kong waters have been reclaimed, intensifying the competition for space between humans and local cetaceans.

The wester waters of Hong Kong serve a critical habitat for two resident cetacean species: the Indo-Pacific humpback dolphins (*Sousa chinensis*, *SC*) and Indo-Pacific finless porpoises (*Neophocaena phocaenoides*, *NP*) (Fig. S1). These species have attracted increasing attention due to the cumulative impacts of natural and anthropogenic stressors. In addition to habitat loss and degradation, these cetaceans face threats from fishing activities, vessel interactions, noise pollution, climate change, prey depletion, and environmental contamination. The cumulative effects of these stressors have imposed significant pressure on both the animals and their habitats, leading to illness, injury, and even mortality. These impacts have been particularly pronounced in recent years.

A substantial decline in the populations of both species is anticipated. Annual abundance estimates of *SC* in HK waters, derived from line-transect surveys, consistently indicate fewer than 100 individuals.[1] One report predicts a population decline of 74% of the population within three generations,[1] making the most severe reduction in the generational history of these cetaceans. For *NP*, abundance estimates remain uncertain; however, previous line-transect surveys recorded a peak of 152 individuals in spring, dropping to just 55 in late autumn. Without immediate and effective conservation measures, the long-term survival of these two populations is highly unlikely.[2]

Text S2. LCMs analysis in cell culture

For liquid culture media samples, the extraction and cleanup procedures were modified based on the method for aqueous environmental samples [3]. 2 mL sample was spiked with $^{13}C_{12}$-PCB-118 and $^{13}C_{12}$-BDE-77 at 10 ng/g as internal standards. After the addition of 5 mL of dichloromethane (DCM), the mixture was vortexed for 1 min and subjected to ultrasonic-assisted extraction for 30 min (480 W, room temperature). The organic layer was then collected. This extraction step was repeated three times, and the combined extracts were dried over anhydrous Na_2SO_4 and concentrated to 1 mL. Cleanup was performed using a packed florisil column (2 g florisil topped with 1 g anhydrous Na_2SO_4). The column was pre-rinsed with 10 mL DCM, and after sample loading, LCMs were eluted with an additional 10 mL DCM. The eluate was concentrated to near dryness and reconstituted in 200 μL of solvent prior to instrumental analysis. The spiked recoveries are provided in Table S7.

Text S3. Suspect screening

Suspect screening of LCMs analogues was performed following a previous study with proper modifications (Fig. S5). A database comprising 1,173 LCM chemicals sourced from patents and published articles was established using Trace Finder 4.0.[4] The suspect screening strategy is shown in Fig. S5. Considering potential isomerization, 871 m/z were analyzed. Except for detecting 38 LCMs in the dolphin samples, only 27 formulas were screened in the tissue samples (Table S12). Confirmation of the suspected LCMs was conducted via GC-Orbitrap. The accurate mass, RT, isotope, and fragments of the suspect chemicals were compared with those of the reference standards. The fragments were interpreted based on a possible fragmentation pattern of the suspect chemicals under an EI source. Apart from the compounds that were detected, two other compounds were structurally determined using the LCMs standards. These two compounds could not be quantified because they were below the detection limit.

As a result, two additional compounds, DFEBB and TFPrBB, were initially identified in the dolphin and porpoise tissue samples. However, both LCMs were below MDL. DFEBB was discovered in sediments from the Yangtze River Delta, with a mean concentration of 2.93 ng/g dw.[5] Classified as Fluorinated Biphenyls and Analogues (FBAs). TFPrBB was initially detected in dust samples obtained from an e-waste dismantling site, exhibiting a high mean concentration of 123 ng/g dw.[6] Importantly, FBAs were predicted to be more toxic than their non-fluorinated LCM counterparts.[6] These two compounds, featuring benzene or cyclohexane rings, displayed experimental Log K_{ow} values that increased to three times or more than predicted values.[7] This observation implies their potential for enhanced retention in sediments and dust.

Text S4. Establishment of primary cetacean skin and kidney fibroblast cultures

Skin and kidney tissues were isolated from a melon-headed whale (*Peponocephala electra*) which stranded in Shantou, Guangdong, China on January 3rd, 2023. The melon-headed whale, also known as the electra dolphin, is a toothed oceanic dolphin belonging to the same family as *SC* (Delphinidae). *SC* and *NP* mainly inhabit coastal waters, and the melon-headed whale is primarily found in deep water world widely. All three species are social marine cetaceans that forage in groups. Importantly, the use of melon-headed whale primary cells was driven by logistical and ethical constraints: primary fibroblasts can only be derived from freshly deceased individuals, *Peponocephala electra* was the only Delphinidae species for which viable primary cells were obtainable. Therefore, this choice ensured a taxonomically relevant cetacean cellular model while adhering to ethical requirements. In addition, the core stress-response endpoints evaluated here (e.g., oxidative stress and cell-cycle regulation) are expected to be broadly conserved across odontocete cetaceans, supporting the use of these primary cetacean cells as a practical surrogate model when SC/NP primary cells are not available.[8] Moreover, skin and kidney fibroblasts were selected as the primary cell model because they are abundant, robust, and technically feasible to derive as viable primary cells from post-mortem tissues, while tissues such as brain, liver, and adipose (where LCMs tend to accumulate) do not reliably yield viable primary cells under field conditions.

The specimens were washed three times in ice-cold PBS and finely chopped into approximately 1 mm^3 pieces. The chopped skin tissues were then plated onto a collagen-coated cell culture plate with a medium containing a 1:1 ratio of high glucose DMEM and DMEM/F12, 20% FBS, 0.1 mM non-essential amino acids, 2 mM L-glutamine, 100 U/mL penicillin, and 100 μg/mL streptomycin. RPMI-1640 was chosen for kidney cells after testing DMEM and DMEM/F-12, as it supported superior proliferation and long-term maintenance. For the kidney tissues, the complete medium contained 90% RMPI-1640 medium, 10% FBS supplied with 2 mM L-glutamine, 100 U/mL penicillin, and 100 μg/mL streptomycin. Once a cell monolayer was formed, the tissue fragments were removed from the plates, and all the cells were collected by Trypsinization. Suspended cells were washed with PBS once and collected by centrifugation. Subsequently, the cell pellet was resuspended and subcultured at a 1:3 ratio. Cells were maintained in a humidified incubator at 37 °C with 5% CO_2 and 95% humidity.

Fibroblasts from the melon-headed whale skin and kidney were established using an explant/outgrowth approach adapted and maintained as primary (non-immortalized) fibroblast-enriched cultures, which were named Melon-Head Skin Fibroblast (MHSF) cels and Melon-Head Kidney Fibroblast (MHKF) cells, respectively.[9] In brief, tissue pieces were plated to allow fibroblast outgrowth, and adherent spindle-shaped cells were expanded by routine trypsin passaging (1:3). Kidney explants yielded predominantly adherent, spindle-shaped cells; no marker-based cell sorting/enrichment (e.g., FACS or immunomagnetic selection) was applied, and thus the culture is conservatively described as a primary kidney-derived fibroblast-like (fibroblast-enriched) adherent cell culture.

Quality control/assurance steps were implemented as follows: (i) all chemical exposure experiments were performed using passages 4–7; (ii) cultures were routinely inspected for stable growth and fibroblast-like morphology prior to exposure; (iii) mycoplasma testing was performed and was negative (Beyotime, C0303S); (iv) penicillin–streptomycin-amphotericin B, supplementation was used during routine culture (Absin, abs9246), and no bacterial contamination was observed; and (v) all experiments were conducted using the same cell isolate (single biological source) to minimize inter-isolate variability.

Although primary cells from brain, liver, and adipose tissues would have been ideal targets given their higher measured LCM burdens, such cell types were not viable from this stranded specimen. Therefore, skin (environmental interface) and kidney (detoxification-associated) fibroblasts were utilized as physiologically relevant primary cell models; future studies will expand to additional cell types and incorporate marker-based phenotypic validation when additional fresh tissues/cells become available.

Text S5. Cell viability assay

To evaluate the cytotoxicity of individual LCMs, cell viability was quantified using the TransDetect Cell Counting Kit (CCK-8) following the manufacturer's protocol. PeCHPrB was dissolved in ethanol to prepare the stock solution, whereas the remaining LCMs were dissolved in DMSO, as specified in Table S9 (Concentration 2). Each compound was further serially diluted (5-fold), and the final solvent concentration in the culture medium was fixed at 0.2% (v/v) for both ethanol and DMSO across all exposure concentrations. Fibroblasts (6,000 cells per well) were seeded into 96-well plates and allowed to adhere for 12 h prior to exposure. Cells were then treated with LCM solutions for 24 h. Matched vehicle controls (0.2% ethanol or 0.2% DMSO, without LCM) were included and used as the baseline for normalization (set to 100%). After exposure, CCK-8 reagent was added and cells were incubated for 2 h at 37°C, 5% CO_2, and 95% humidity. Absorbance at 450 nm was recorded using a microplate reader.

Text S6. Method of quantitative PCR validation

Quantitative PCR analysis was conducted in triplicate to quantify the relative expression levels of DEGs related to the cell cycle in MHSF and MHKF cells following LCM interventions. The same batch of harvested cells was used for qPCR. Total RNA was extracted using TRIzol reagents (Cat: #15596018CN, Thermo Fisher Scientific) according to the manufacturer's instructions. RNA concentration and purity were assessed using a NanoDrop spectrophotometer (Thermo Fisher Scientific, Waltham, MA, USA), with only samples meeting the A260/A230 and A260/A280 ratios between 1.8-2.0 being processed further. RNA integrity was verified via agarose gel electrophoresis. Only QC-qualified RNA was used for subsequent reverse transcription (qPCR) and RNA-seq library preparation. The extracted RNA was reverse-transcribed into cDNA using a PrimeScript™ RT reagent Kit with gDNA Eraser (Cat: # RR047Q, Takara, Dalian, China). Genomic DNA was removed using the gDNA Eraser step according to the manufacturer's protocol. Primers for target genes were designed using Primer3 based on transcript data from RNA sequencing (Table S14). Quantitative PCR was performed using the TB Green Premix Ex Taq II kit (Cat: # RR820Q, Takara, Dalian, China) on a Light Cycler 480 (Roche, Seattle, WA, USA) following the manufacturer's instructions. qPCR measurements were performed as technical triplicates from the same biological preparation. GAPDH was used as the housekeeping gene, and data analysis was conducted using $2^{-\triangle\triangle Ct}$ method (Pfaffl 2001).

Text S7. Positive matrix factorization-multiple linear regression (PMF-MLR)

PMF was conducted using EPA PMF 5.0 software developed by the U.S. Environmental Protection Agency (www.epa.gov) following standard procedures (https://www.epa.gov/air-research/positive-matrix-factorization-model-environmental-data-analyses).[10] [11]The PCA analysis was done using IBM SPSS Statistics® version 19.0 before PMF analysis. The original concentrations for individual LCMs are extracted from Table S15. Only compounds with DF > 20% within the cetacean tissue dataset (all tissue samples listed in Table S2) and with sample-level ΣLCMs (total LCMs in that sample) > 10 ng/g were included in the PCA screening and subsequent PMF analysis. The method limits of quantification (MLOQ) was defined as an instrumental signal-to-noise ratio > 10. For analytes that were not detected or had levels less than MLOQ, 1/2 MLOQ were adopted. The data uncertainties (Unc) were determined using the following Equation S1 and S2, where error fraction refers to the relative standard deviation of LCMs concentrations in the same sample.

$$\text{Unc} = \frac{5}{6} \times \text{MLOQ} \qquad \text{concentration} < \text{MLOQ} \qquad \text{(S1)}$$

$$\text{Unc} = \sqrt{(\text{error fraction} \times \text{concentration})^2 + (0.5 \times \text{MLOQ})^2} \quad \text{concentration} \geq \text{MLOQ} \qquad \text{(S2)}$$

PMF is a result-feedback approach. To obtain the best number of factors, two-factor, three-factor or four-factor solutions were conducted after data input. Three error estimation methods were performed to evaluate the variability and validity of the PMF solutions: displacement (DISP), bootstrap (BS), and BS-DISP [9-11], and the results are listed in Table S13. For DISP results, the maximum observed drop of Q should be less than 1%, and no swap counts for all the PMF factors for the lowest dQmax levels (dQmax = 4). All solutions passed the criteria. In BS error estimation, the BS method was run 100 times. An ideal solution could be that there should be no data sets considered as "unmapped", and over 80% of factors mapped should be interpreted, two-factor and three-factor is considered appropriate. Obviously, four-factor solution did not pass the criteria. For BS-DISP results, a large value is not necessarily alarming, but it indicates that there was at least one resample where a deeper minimum appeared. For Swaps by Factor (dQmax = 0.5), all of the swaps are zero in the best case. Based on the above error estimation results, two-factor was the best solution. Besides, calculated R^2 for LCMs were all greater than 0.8. In EPA PMF, species were categorized based on signal-to-noise (S/N) ratios as strong or weak, and weak species were down-weighted following the EPA PMF guidance. For clarity, Figure 4a displays only the strong species with the highest contributions in each factor profile, while weak species were retained in the model fitting but are not emphasized in the main figure.

For the estimation of PMF factor mass apportionment and contributions, MLR was performed using IBM SPSS Statistics 26.0 between the ΣLCMs values ($y_{\Sigma LCMs}$) and the source contribution matrix (G matrix, G_1 ~ G_2), It can be seen from the coefficient analysis table that G1 and G2 sig are <0.001 and less than 0.05, with a 95% probability of rejecting the null hypothesis, indicating that the regression coefficients are significant. The PMF-MLR equation was determined as Equation S3 (Isley et al., 2022; Sofowote et al., 2008; Zhang et al., 2022b). The factor *i* source percentage contribution was predicted using Equation S4. The predicted each factor source to the ΣLCMs values in each sample could be obtained by multiplying the MLR coefficient (ng/g) of each source by the corresponding G matrix element (g/g).

$$Y_{\Sigma LCMs} = \sum_{i=1}^{n} F_i \times G_i = 2.629\text{G}_1 + 1.896\text{G}_2 \quad \text{R}^2 = 0.828 \qquad \text{(S3)}$$

$$i \text{ source contribution} = F_i / \sum F_i \qquad \text{(S4)}$$

Text S8. Unit harmonization for cross-matrix comparison (Figure 2a)

To enable direct comparison across matrices, all literature-reported maximum (reported max) ΣLCMs values were converted to ppb. For solids/biota, 1 ppb = 1 ng/g; for aqueous matrices, 1 ppb = 1 μg/L. For air, ppb was expressed on a mass basis (ppb(w), μg/kg air) by converting from mass concentration using an air density of 1.184 kg/m^3 (25 °C, 1 atm): ppb(w) = C(μg/m^3)/ρ(kg/m^3). The values used in Figure 2a were:

Fish: max ΣLCMs = 1367 ng/g lw → 1367 ppb.[12]

Indoor dust: max ΣLCMs = 448 ng/g dw → 448 ppb. [13]

Marine cetaceans (**this study**): max ΣLCMs = 203 ng/g dw → 203 ppb.

Sediment: max ΣLCMs = 31.1 ng/g dw → 31.1 ppb.[5]

Invertebrates/fish (ww): max ΣLCMs = 29.7 ng/g ww → 29.7 ppb.[14]

Landfill leachate: max ΣLCMs = 1120 ng/L → 1.12 ppb.[15]

Wastewater: 16.8 ng/L (reported as mean; no explicit max reported) → 0.0168 ppb.[3]

Ambient air: max ΣLCMs (gas + particle) = 13,500 pg/m^3 (=0.0135 μg/m^3) → 0.0114 ppb(w).[16]

Text S9. Alteration of gene expression related to cell cycle

To further confirm the effect of LCMs on cell cycle of MHSF and MHKF cells, we quantified the mRNA expression of related genes using quantitative PCR (qPCR). Sequencing and RT-PCR are two different technologies with different principles and calculation formulas, and it is often difficult to completely match.[17] Although some differences in gene expression were observed between qPCR and RNA sequencing ($p < 0.05$), the overall gene expression patterns were consistent across both methods. This variation likely stems from the inherent differences in the principles and calculations of the two techniques.

In MHSF cells, MOPrCHB significantly suppressed the expression level of genes including *CDK1*, *CDK2*, *CDK6*, *CCND1*, *CCNA2*, *MCM3*, *MCM4*, and *PCNA*. In contrast, EDFPPB up-regulated the expression level of *CKD2*, *MCM3*, while MPHBB, PCTB, MPCB, and PPB increased the expression of *CDK4*, *MCM3*, and *MCM4*. MPHBB also elevated *CDK2*, *CDK6*, *PCNA* and *TGF-β1* (Fig. S10).

In MHKF cells, a more complex gene expression pattern was observed (Fig. S11). PeCHPrB up-regulated the expression level of *CCND1*, *MCM4*, and *TGF-β1*, while down-regulating *PCNA*. MOPrCHB, EDFPPB and EBMB up-regulated the gene expression level of CDK2, CCND1, and MCM4. Additionally, MOPrCHB increased the expression of *CDK4*, *MCM3*, and *TGF-β1*, while EBMB up-regulated *CDK1* and *MCM3*. MOPrCHB, EDFPPB, and MPHBB down-regulated *CCNA2* and *PCNA*. Both PCTB and MPCB up-regulated *CDK2*, *CCND1*, *MCM3*, *MCM4*, and *TGF-β1*, with PCTB also increasing *CDK1* expression. PPB up-regulated *CCND1* and *CCNA2*.

Table S1. Detailed information of LCM standards.

Abbr.	CASRN	Name	Structure	Half-life in water (day)	Suppliers	Purity	Log (Kow)	MW (g/mol)
PPB	279246-65-0	1-(prop-1-enyl)-4-(4-propylcyclohexyl)cyclohexane	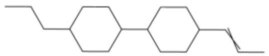	37.5	TCI	99%	6.42	248.5
EDFPrB	174350-05-1	1-ethoxy-2,3-difluoro-4-(4-propylcyclohexyl)benzene	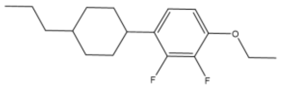	180	TCI	99%	8.45	282.4
MPB	64835-63-8	4-methyl-4'-pentylbiphenyl	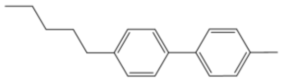	15	TCI	99%	4.34	238.4
EDFPPB	157248-24-3	1-ethoxy-2,3-difluoro-4-(4-propylphenyl)benzene	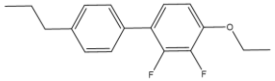	180	LCM factories	99%	7.18	276.3
ETFB	650634-92-7	4-(trans-4-Ethylcyclohexyl)-4'-(trifluoromethoxy)biphenyl	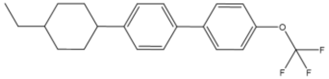	60	LCM factories	99%	6.82	348.4
EBMB	155041-85-3	1-[(trans,trans)-4'-Ethenyl[1,1'-bicyclohexyl]-4-yl]-4-methyl-benzene	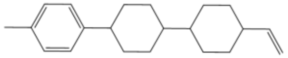	37.5	TCI	99%	6.26	282.5
DFTDFPB	303186-20-1	4-[difluoro(3,4,5-trifluorophenoxy)methyl]-3,5-difluoro-4'-propylbiphenyl	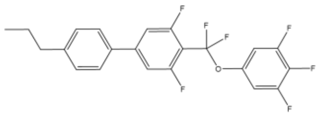	180	TCI	99%	4.37	428.4
MPCB	84656-75-7	1-methyl-4-(4-(4-propylcyclohexyl)cyclohexyl)benzene	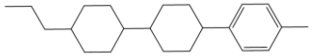	37.5	TCI	99%	8.53	298.5
DFMTDFPB	1690317-23-7	4-[difluoro(2-methyl-3,4,5-trifluorophenoxy)methyl]-3,5-difluoro-4'-propylbiphenyl	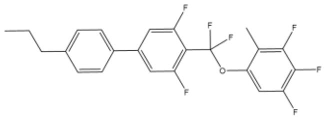	180	LCM factories	99%	7.36	442.4
DFMPCB	431947-34-1	2,3-difluoro-1-methoxy-4-(4-(4-propylcyclohexyl)cyclohexyl)benzene	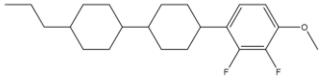	180	LCM factories	99%	8.82	350.5
EPB	84540-37-4	1-ethyl-4-(4-(4-propylcyclohexyl)phenyl)benzene	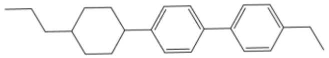	37.5	J&K	98%	9.94	306.5

DFEEPB	323178-01-4	2,3-difluoro-1-ethoxy-4-(4-(4-ethylcyclohexyl)phenyl)benzene	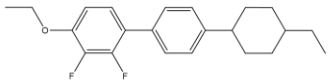	180	LCM factories	99%	9.1	344.5
EDFPBB	123560-48-5	1-ethoxy-2,3-difluoro-4-(4-(4-propylcyclohexyl)cyclohexyl)benzene	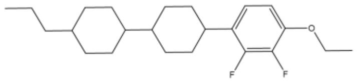	180	LCM factories	99%	5.35	364.5
EFPT	95759-44-7	4''-ethyl-2'-fluoro-4-propyl-1,1':4',1''-terphenyl	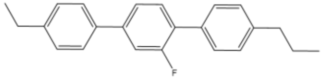	60	LCM factories	99%	9.44	318.4
EDFPB	189750-98-9	4-ethoxy-2,3-difluoro-4'-(4-propylcyclohexyl)biphenyl	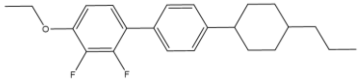	180	LCM factories	99%	5.84	358.5
DFPPCB	473257-14-6	2,3-difluoro-1-propoxy-4-(4-(4-propylcyclohexyl)cyclohexyl)benzene	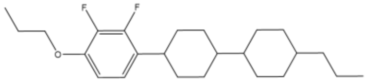	180	LCM factories	99%	9.65	378.6
BCEDFB	473257-15-7	1-(4-(4-butylcyclohexyl)cyclohexyl)-4-ethoxy-2,3-difluorobenzene	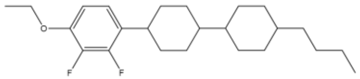	180	LCM factories	99%	9.38	378.6
BEFT	825633-75-8	4-butyl-4''-ethyl-2'-fluoro-1,1':4',1''-terphenyl	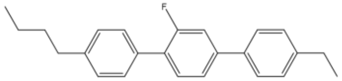	60	LCM factories	99%	9.01	332.5
DFEBB	139195-63-4	3,4-difluoro-4'-[4'-ethyl-1,1'-bi(cyclohexyl)-4-yl]biphenyl	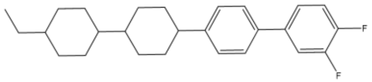	180	LCM factories	99%	6.82	382.5
DFMDFPTPB	NA	4-[difluoro(3,4,5-trifluorophenoxy)methyl]-3,5-difluoro-4'-[(5-propyl-tetrahydro-2H-pyran)-yl]-biphenyl	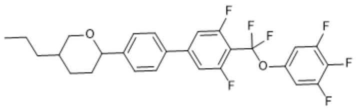	180	LCM factories	99%	8.46	512.5
TDFPCT	524709-77-1	4-trifluoromethoxy-3,5-difluoro-2'-fluoro-4''-(4-propylcyclohexyl)-1,1':4',1''-terphenyl	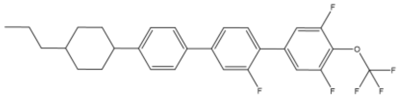	180	LCM factories	99%	9.87	492.5
PDTPMTT	303186-36-9	4''-Propyl-4-[difluoro(3,4,5-trifluorophenoxy)methyl]-2',3,5-trifluoro-1,1':4',1''-terphenyl	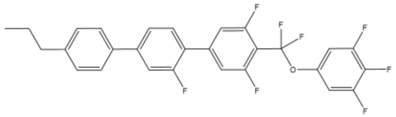	180	LCM factories	99%	8.29	522.4
DMTMDETPB	1700444-88-7	4-[difluoro(2-methyl-3,4,5-trifluorophenoxy)methyl]-3,5-difluoro-4'-[(5-ethyl-tetrahydro-2H-pyran)-yl]-biphenyl	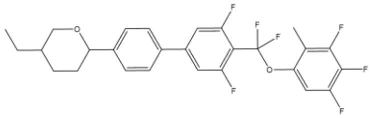	180	LCM factories	99%	8.95	512.5

DPrBB	119990-81-7	3,4-difluoro-4'-[4'-propyl-1,1'-bi(cyclohexyl)-4-yl]biphenyl	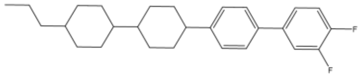	180	LCM factories	99%	10.36	396.6
DBBB	119990-82-8	3,4-difluoro-4'-[4'-butyl-1,1'-bi(cyclohexyl)-4-yl]biphenyl	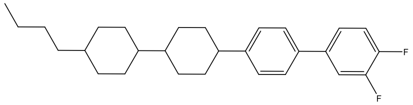	180	LCM factories	99%	10.36	410.6
DPeBB	136609-96-6	3,4-difluoro-4'-[4'-pentyl-1,1'-bi(cyclohexyl)-4-yl]biphenyl	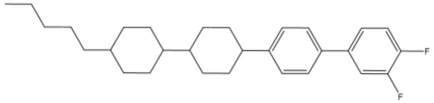	180	LCM factories	99%	8.78	424.6
DPB	118164-49-1	3,4-difluoro-4'-propyl-1,1'-Biphenyl	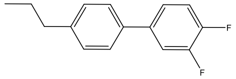	60	LCM factories	99%	10.57	232.3
2CB	58743-75-2	4'-ethylbiphenyl-4-carbonitril	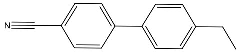	37.5	LCM factories	99%	7.58	207.3
3OCB	52709-86-1	4'-propoxy-4-biphenylcarbonitrile	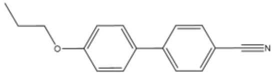	37.5	LCM factories	99%	10.04	237.3
PCTB	133937-72-1	4-propyl-4'-[4-(trifluoromethoxy)phenyl]-1,1'-bicyclohexyl	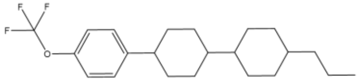	180	TCI	>98%	11.36	368.5
ETeT	326894-55-7	4"-ethyl-2',3,4,5-tetrafluoro-1,1':4',1"-terphenyl	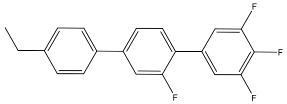	180	LCM factories	99%	11.07	330.3
5OCB	52364-71-3	4-cyano-4'-pentyloxybiphenyl	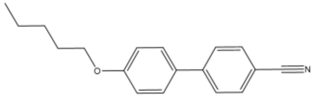	15	LCM factories	99%	10.1	265.4
6OCB	41424-11-7	4'-hexyloxy-4-biphenylcarbonitrile	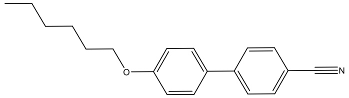	37.5	LCM factories	99%	11.06	279.4
8OCB	52364-73-5	4'-(octyloxy)-4-biphenylcarbonitrile	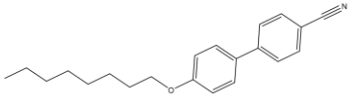	37.5	LCM factories	99%	11.55	307.4

5CT	54211-46-0	4-cyano-4''-pentyl-p-terphenyl	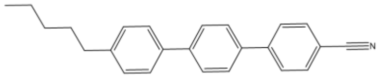	37.5	LCM factories	99%	12.04	325.5
BPCHB	85600-56-2	4,4'-bis(4-propylcyclohexyl)biphenyl	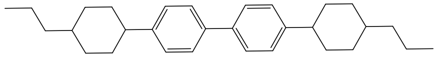	60	LCM factories	99%	12.19	402.7
PCPCB	80955-71-1	4-(4-pentylcyclohexyl)-4'-(4-propylcyclohexyl)biphenyl	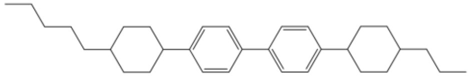	37.5	LCM factories	99%	13.17	430.7
MPhBB	129738-42-7	4-(4-methylphenyl)-4’-(3-butenyl)bicyclohexyl	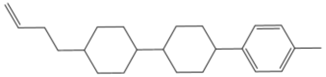	37.5	Aladdin	98%	9.8	310.53
PeCHPrB	82991-48-8	1-(trans-4-Pentylcyclohexyl)-4-propylbenzene	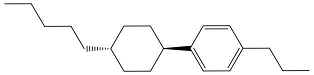	37.5	Bidepharm	98%	8.72	272.5
EOPrCHB	80944-44-1	1-Ethoxy-4-(trans-4-propylcyclohexyl)benzene	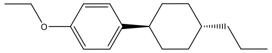	37.5	Bidepharm	98%	6.78	246.4
EPhEMOB	63221-88-5	1-(2-(4-ethylphenyl)ethynyl)-4-methoxybenzene	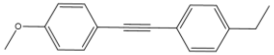	37.5	TCI	98%	5.14	236.3
BDPrB	208709-55-1	1-Butoxy-2,3-difluoro-4-(trans-4-propylcyclohexyl)benzene	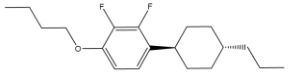	60	Bidepharm	95%	8.16	310.4
TFrPrB	132123-39-8	3,4,5-Trifluoro-4'-(trans-4-propylcyclohexyl)biphenyl	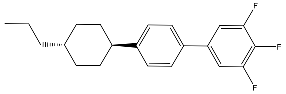	180	Bidepharm	95%	8.57	332.4
DFTMDEB	303186-19-8	4-[Difluoro(3,4,5-trifluorophenoxy)methyl]-3,5-difluoro-4'-ethyl-biphenyl	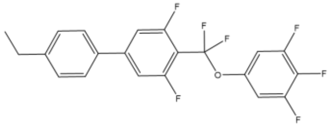	180	Aladdin	99%	8.61	414.3
PFPT	95759-51-6	4''-Propyl-2'-fluoro-4-pentyl-1,1':4',1''-terphenyl	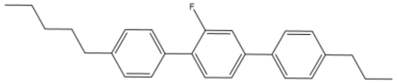	60	Aladdin	99%	9.76	360.5
TeFPT	205806-87-7	2',3,4,5-Tetrafluoro-4''-propyl-1,1':4',1''-terphenyl	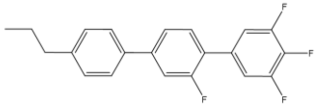	180	TCI	99%	7.85	344.4

FPB	87260-24-0	4-fluoro-4'-(trans-4-propylcyclohexyl)biphenyl	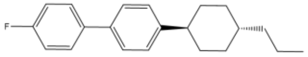	60	Aladdin	98%	8.17	296.4
DFMPMB	1373116-00-7	2,3-Difluoro-1-methoxy-4-[(trans-4-propylcyclohexyl)]benzene	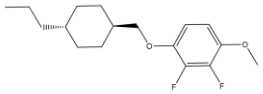	180	TCI	98%	6.72	298.4
EBDFMB	174350-08-4	1-[(trans,trans)-4'-Ethyl[1,1'-bicyclohexyl]-4-yl-2,3-difluoro-4-methyl-benzene	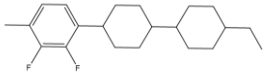	180	Bidepharm	98%	9.35	320.5
DFPrCB	82832-57-3	1,2-Difluoro-4-[trans-4-(trans-4-propylcyclohexyl)cyclohexyl]benzene	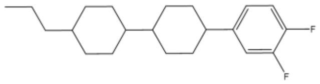	180	Bidepharm	98%	9.3	320.5
DFEB	134412-18-3	3,4-Difluoro-4'-(trans-4-ethylcyclohexyl)biphenyl	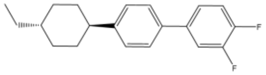	180	TCI	98%	7.88	378.5
TFPrCB	131819-23-3	1,2,3-trifluoro-5-[3-(3-propylcyclohexyl)cyclohexyl]benzene	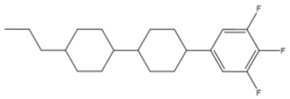	180	Bidepharm	98%	9.5	300.4
EDFPeB	124729-02-8	1-Ethoxy-2,3-difluoro-4-(trans-4-pentylcyclohexyl)benzene	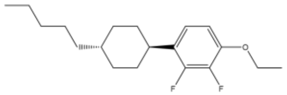	60	TCI	98%	8.16	310.4
ECTFB	135734-59-7	1-[4-(4-ethylcyclohexyl)cyclohexyl]-4-(trifluoromethoxy)benzene	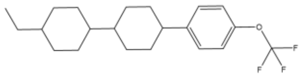	60	Leyan	98%	9.45	354.5
FPCB	82832-27-7	1-Fluoro-4-[4-(4-propylcyclohexyl)-cyclohexyl]benzene	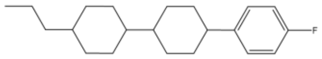	60	Macklin	98%	9.1	302.5
TFECB	139215-80-8	3,4,5-Trifluoro-1-[trans-4'-(trans-4"-ethylcyclohexyl)cyclohexyl]benzene	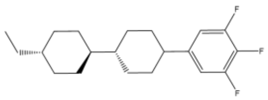	180	Leyan	98%	9.01	324.4
DFPrB	85312-59-0	3,4-Difluoro-4'-(trans-4-propylcyclohexyl)biphenyl	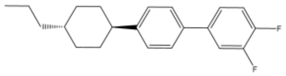	180	Aladdin	98%	8.37	314.4

TFPrBB	137529-41-0	3,4,5-trifluoro-4'-[(trans,trans)-4'-Propyl[1,1'-bicyclohexyl]-4-yl]biphenyl	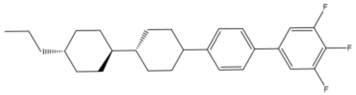	180	Aladdin	98%	11.26	414.6
TeFPrB	173837-35-9	2',3,4,5-Tetrafluor-4'-(trans-4-propylcyclohexyl)biphenyl	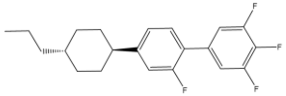	180	Aladdin	98%	8.78	350.4
TrFPeB	137019-95-5	3,4,5-Trifluoro-4'-(trans-4-pentylcyclohexyl)biphenyl	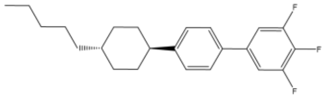	180	Bidepharm	98%	9.56	360.5
TFPeCB	137644-54-3	3,4,5-Trifluoro-1-[trans-4'-(trans-4''-pentylcyclohexyl)cyclohexyl]benzene	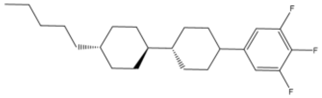	180	Bidepharm	98%	10.48	366.5
MOPrCHB	81936-32-5	1-Methoxy-4-(trans-4-propylcyclohexyl)benzene	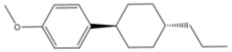	37.5	Bidepharm	98%	6.29	232.4

Table S2. Sample information for porpoises (*NP*) and dolphins (*SC*).

Sample No.	Year of collection	Sex	Length (cm)	Blubber	Blubber lipid content (%)	Brain	Muscle	Liver	Kidney
NP-T01	2018	F	150	√	25%				
NP-T02	2018	F	78	√	39%	√	√		
NP-T03	2018	M	140	√	29%				
NP-T04	2018	F	145	√	38%				
NP-T05	2019	F	150	√	70%				
NP-T06	2019	M	166	√	43%				
NP-T07	2019	F	154				√	√	
NP-T08	2019	M	137	√	45%				
NP-T09	2019	M	150				√		
NP-T10	2019	M	150	√	61%				
NP-T11	2019	M	162	√	50%		√		
NP-T12	2019	TBC	TBC					√	
NP-T13	2019	F	140	√	74%				
NP-T14	2020	M	150	√	61%		√		
NP-T15	2020	F	127	√	36%	√	√		
NP-T16	2020	F	167			√	√		
NP-T17	2020	F	167					√	
NP-T18	2020	F	167				√		
NP-T19	2021	F	128			√			
NP-T20	2021	M	180				√	√	√
NP-T21	2021	M	175			√			
NP-T22	2021	F	90			√			
NP-T23	2021	TBC	TBC				√		
NP-T24	2021	F	176	√	89%				
NP-T25	2021	M	167	√	78%		√		
NP-T26	2021	M	167	√	73%	√			√
SC-01	2018	F	177				√		
SC-02	2018	F	78	√	50%		√		
SC-03	2018	F	249	√	29%	√			

SC-04	2018	F	200				√		
SC-05	2018	M	99	√	43%		√		
SC-06	2018	F	91	√	39%		√		
SC-07	2019	F	265	√	92%		√		
SC-08	2020	F	127					√	
SC-09	2020	F	92	√	43%		√		
SC-10	2020	F	194				√		
SC-11	2020	F	243	√	59%	√	√		
SC-12	2020	M	~100	√	93%		√		
SC-13	2020	M	260				√		
SC-14	2021	TBC	TBC				√		
SC-15	2021	M	240	√	59%				√
SC-16	2021	TBC	TBC				√		

TBC represents "to be confirmed". The carcass of the stranded dolphin was too decomposed to determine its body length or sex, so both were recorded as indeterminate.

322

Table S3. Blubber samples used for temporal trend analysis.

Sample No.	Year of collection	Gender	Body length (cm)	Lipid (%)
NP-01	2007	M	77	79%
NP-02	2007	M	142.5	29%
NP-03	2007	M	154	33%
NP-04	2008	F	77.5	76%
NP-05	2008	F	88	81%
NP-06	2008	F	163	78%
NP-07	2009	M	148	91%
NP-08	2009	M	141	29%
NP-09	2009	M	158	35%
NP-10	2009	M	127	52%
NP-11	2010	M	116	90%
NP-12	2010	M	174.5	53%
NP-13	2010	F	74	81%
NP-14	2010	M	169.5	54%
NP-15	2013	M	168	55%
NP-16	2013	F	172	17%
NP-17	2013	M	122	65%
NP-18	2013	F	69	16%
NP-19	2014	F	81	10%
NP-20	2014	M	143	35%
NP-21	2014	F	164.5	39%
NP-22	2014	F	160	31%
NP-23	2014	M	150	55%
NP-24	2015	F	158	30%
NP-25	2015	M	＞110	19%
NP-26	2015	F	117	24%
NP-27	2015	M	169	39%
NP-28	2017	M	139.5	36%
NP-29	2017	M	108	22%
NP-30	2017	M	142	59%

NP-31	2017	F	174	39%
NP-32	2018	F	154	50%
NP-33	2018	F	145	59%
NP-34	2018	M	108	22%
NP-35	2018	F	150	25%
NP-36	2018	F	78	39%
NP-37	2018	M	140	29%
NP-38	2018	F	145	38%
NP-39	2019	M	137	45%
NP-40	2019	M	166	43%
NP-41	2019	M	150	75%
NP-42	2019	M	162	50%
NP-43	2019	F	140	74%
NP-44	2020	M	150	61%
NP-45	2020	F	127	36%
NP-46	2021	F	176	89%
NP-47	2021	M	167	78%
NP-48	2021	M	167	73%

Table S4. Retention times, quantification ions, specific fragment ions, and standard working curves for the LCMs. Calibration curves were constructed using 6–9 concentration points selected from 0.01, 0.05, 0.1, 0.5, 1, 5, 10, 50, and 100 µg/L.

No.	Abbr.	Retention time (min)	Quantitative Ion $[M+H]^+$	Specific Fragment Ion 1	Specific Fragment Ion 2	Standard Working Curve	
						R2	Linearity (ng/mL)
1	DPB	5.95	203.0666	232.1058	183.0604	0.9996	0.5-100
2	MOPrCHB	6.67	232.1819	147.0802	134.0726	0.9995	0.5-100
3	PPB	6.69	248.2499	123.1168	205.1949	0.9995	0.1-100
4	EOPrCHB	6.99	246.1975	133.0647	161.0959	0.9996	0.5-100
5	2CB	7.04	192.0807	165.0701	207.1043	0.999	0.05-100
6	EBDFMB	7.04	282.1791	156.0382	169.046	0.9993	0.5-100
7	EDFPrB	7.44	238.1716	181.101	165.0698	0.9993	0.05-100
8	MPB	7.44	276.132	219.0614	247.0928	0.999	0.05-100
9	DFMPMB	7.88	160.033	145.0096	298.1738	0.9993	0.5-100
10	PeCHPrB	8.08	272.2496	117.0698	146.109	0.9996	0.5-100
11	BDPrB	8.33	310.2098	254.1476	156.038	0.9994	0.5-100
12	EDFPeB	8.41	310.2098	156.0379	197.0772	0.9995	0.5-100
13	EPhEMOB	8.55	236.1193	221.0958	178.0777	0.9992	0.5-100
14	3OCB	8.60	195.0678	166.0652	237.1149	0.9992	0.01-100
15	ECTFB	8.85	354.2156	175.0362	188.044	0.9997	0.5-100
16	TFECB	8.89	324.2054	145.0259	158.0337	0.9992	0.5-100
17	ETFB	9.39	348.1693	277.0834	264.0757	0.9992	0.05-100
18	DFTMDEB	9.51	252.0554	267.0788	183.0603	0.9994	0.5-100
19	DFEB	9.53	300.1682	216.0744	229.0822	0.9992	0.5-100
20	ETeT	9.72	330.1025	315.079	275.0668	0.9993	0.05-100
21	PCTB	9.83	368.2318	188.0443	175.0365	0.9993	0.5-100
22	EBMB	9.85	282.2342	171.1169	118.0777	0.9999	0.05-100
23	TFPrCB	9.88	338.2212	203.1793	158.0338	0.9994	0.5-100
24	TeFPrB	9.91	350.1644	252.0552	239.0477	0.9995	0.5-100
25	FPCB	9.99	302.2399	109.0447	122.0525	0.9994	0.5-100
26	DFPrCB	10.11	320.2303	127.0352	140.043	0.9996	0.5-100
27	EDFPPB	10.24	320.2304	141.0509	154.0587	0.9998	0.5-100
28	TFrPrB	10.26	332.1739	234.0647	247.0728	0.9994	0.5-100
29	DFTDFPB	10.52	281.0948	252.0557	232.0495	0.9991	0.5-100
30	DFPrB	10.63	314.1836	216.0742	229.082	0.9991	0.5-100
31	FPB	10.63	296.1929	196.0683	211.0918	0.9994	0.5-100
32	5OCB	10.75	195.0678	166.0652	265.1462	0.9993	0.05-100
33	TeFPT	10.79	344.1176	315	275.0667	0.9992	0.5-100
34	MPCB	11.09	298.2654	118.0777	131.0856	0.9994	0.05-100
35	6OCB	11.79	195.0678	166.0652	279.1618	0.9991	0.5-100
36	DMTMDETPB	11.82	281.0948	252.0557	232.0495	0.9992	0.1-100
37	MPhBB	12.05	310.2651	118.0776	117.0699	0.9992	0.5-100

38	TFPeCB	12.06	366.2522	145.0259	231.2107	0.9996	0.5-100
39	TrFPeB	12.34	360.2053	234.0647	247.0729	0.9997	0.5-100
40	DFMPCB	12.63	350.2414	170.0538	127.0354	0.9993	0.05-100
41	EPB	12.77	306.2341	221.1324	193.1012	0.9994	0.1-100
42	DFEEPB	12.79	344.1945	316.1631	232.0693	0.9998	0.05-100
43	EDFPBB	13.09	364.2571	156.0381	184.0694	0.9995	0.1-100
44	EFPT	13.23	318.1777	289.1385	274.1151	0.9993	0.5-100
45	8OCB	13.46	307.1931	195.0679	166.0652	0.9992	0.5-100
46	EDFPB	13.53	358.2101	330.1789	245.0772	0.9992	0.05-100
47	DFPPCB	13.81	336.2258	378.2728	156.0382	0.9993	0.1-100
48	BCEDFB	13.86	378.2727	184.0694	156.0381	0.9991	0.5-100
49	BEFT	13.97	332.1934	289.1386	274.1152	0.9991	0.1-100
50	PFPT	15.18	360.2241	274.1148	303.1538	0.9994	0.5-100
51	DFEBB	15.40	382.2464	229.0824	216.0745	0.9992	0.1-100
52	5CT	15.65	268.1122	325.1825	/	0.9993	1-100
53	DFMDFPTPB	15.69	365.1522	267.0425	239.0479	0.9992	0.1-100
54	TDFPCT	15.71	394.0784	407.0865	492.1878	0.9992	0.1-100
55	TFPrBB	15.76	414.252	221.0569	234.0647	0.9995	0.5-100
56	PDTPMTT	15.84	375.1164	346.0772	275.0668	0.9993	0.5-100
57	DMTMDETPB	15.90	351.1366	170.0527	239.0479	0.9991	0.1-100
58	DPrBB	16.00	396.2622	203.0667	216.0745	0.9993	0.1-100
59	DBBB	16.72	410.2779	203.0667	216.0745	0.9998	0.5-100
60	DPeBB	17.54	424.2938	216.0745	229.0824	0.9993	0.5-100
61	BPCHB	18.56	402.328	304.2186	317.2264	0.9995	0.5-100
62	PCPCB	21.19	430.3593	304.2186	332.25	0.9994	0.5-100

Table S5. Spike recoveries of 62 LCMs assessed in blubber matrix (selected as the highest-lipid tissue to represent worst-case extraction efficiency). Low concentration spike = 10 μg/L; high concentration spike = 50 μg/L.

Abbr.	Recoveries (%)				ME (%)	Instrument Detection Limit (IDL, ng/ml)	*MLOQ (ng/g)
	Low Conc. Spike Recovery (*n*=3)	RSD	High Conc. Spike Recovery (*n*=3)	RSD			
DPB	72.4±1.0	1.1	84.1±3.1	3.7	84.1	0.05	0.15
MOPrCHB	79.0±2.6	3.3	82.7±4.4	5.3	84.2	0.05	0.15
PPB	79.6±9.9	12.4	101.5±17.5	17.2	91.3	0.5	1.5
EOPrCHB	83.2±2.9	2.6	84.8±4.6	5.4	84.3	0.1	0.3
2CB	83.8±4.3	5.0	84.0±7.6	9.0	98.2	0.05	0.15
EBDFMB	81.2±0.9	1.0	84.4±5.1	6.0	84.9	0.1	0.3
EDFPrB	85.0±0.8	0.4	83.0±3.6	4.3	96.0	0.05	0.15
MPB	88.4±1.1	1.3	84.6±3.4	4.0	95.6	0.05	0.15
DFMPMB	89.3±1.2	0.7	86.3±5.0	5.8	75.2	0.05	0.15
PeCHPrB	105.6±4.7	2.1	93.8±0.3	0.3	82.2	0.5	1.5
BDPrB	80.0±5.2	6.0	88.0±5.3	6.0	80.7	0.05	0.15
EDFPeB	87.7±0.5	0.5	90.4±4.4	4.9	81.4	0.05	0.15
EPhEMOB	92.0±0.9	0.8	92.7±4.7	5.0	105.5	0.05	0.15
3OCB	83.3±1.6	1.8	85.2±8.2	9.6	109.9	0.05	0.15
ECTFB	85.3±2.5	3.0	87.9±5.3	6.1	96.3	0.1	0.3
TFECB	81.9±3.9	4.8	85.7±3.4	4.0	89.6	0.1	0.3
ETFB	85.9±0.6	0.4	86.5±4.6	5.3	98.0	0.05	0.15
DFTMDEB	88.8±2.7	2.7	89.9±5.8	6.5	106.8	0.1	0.3
DFEB	85.2±1.4	0.9	87.7±4.1	4.7	98.9	0.01	0.03
ETeT	85.2±2.1	1.0	86.3±5.4	6.2	101.7	0.01	0.03
PCTB	84.6±2.5	1.4	85.2±3.0	3.5	93.1	0.1	0.3
EBMB	96.2±8.5	3.1	88.3±7.6	8.6	85.8	0.05	0.15
TFPrCB	86.4±2.0	1.6	90.4±5.6	6.2	104.5	0.1	0.3
TeFPrB	85.4±2.0	2.4	83.4±4.5	5.4	97.7	0.1	0.3
FPCB	82.9±0.7	0.6	90.1±2.7	3.0	91.2	0.1	0.3
DFPrCB	85.1±2.9	2.4	86.6±5.7	6.6	86.6	0.01	0.03
EDFPPB	80.1±1.5	1.8	87.2±4.5	5.2	88.4	0.01	0.03
TFrPrB	88.2±2.8	1.1	84.3±3.2	3.8	92.3	0.1	0.3
DFTDFPB	91.0±1.1	0.4	86.1±5.5	6.3	103.1	0.01	0.03
DFPrB	89.8±1.3	0.5	86.6±3.1	3.5	92.8	0.1	0.3
FPB	90.3±0.7	0.5	88.3±3.7	4.2	87.0	0.05	0.15
5OCB	65.3±16.7	15.5	78.2±7.2	9.2	92.1	0.1	0.3
TeFPT	65.5±6.6	3.5	71.2±6.9	10.9	88.3	0.1	0.3
MPCB	96.1±8.7	8.9	83.8±2.7	3.3	81.1	0.1	0.3
6OCB	98.7±0.9	0.9	91.1±10.2	11.2	95.2	0.05	0.15
DMTMDETPB	100.2±2.6	2.3	87.5±6.2	7.1	96.7	0.01	0.03

MPhBB	122.4±5.6	4.6	92.3±2.7	2.9	82.5	0.5	1.5
TFPeCB	90.3±1.5	1.2	85.4±6.3	7.4	80.7	0.5	1.5
TrFPeB	98.4±3.4	3.2	90.7±4.7	5.2	82.9	0.1	0.3
DFMPCB	99.9±2.6	0.9	86.8±4.3	5.0	85.5	0.05	0.15
EPB	102.7±3.2	3.0	89.7±5.3	5.9	80.5	0.01	0.03
DFEEPB	103.9±3.9	1.5	87.0±6.6	7.6	85.8	0.05	0.15
EDFPBB	106.5±4.1	2.3	90.5±5.2	5.8	82.8	0.1	0.3
EFPT	109.9±1.6	1.4	91.9±6.0	6.5	74.4	0.1	0.3
8OCB	111.5±0.6	0.3	105.0±13.2	12.6	95.1	0.1	0.3
EDFPB	117.3±3.1	2.1	93.0±5.0	5.3	79.4	0.05	0.15
DFPPCB	119.7±2.9	1.3	93.1±4.6	4.9	70.7	0.1	0.3
BCEDFB	112.2±2.5	1.5	93.5±6.4	6.8	71.4	0.1	0.3
BEFT	121.2±5.7	4.7	97.3±8.5	8.8	70.6	0.01	0.03
PFPT	112.2±1.8	1.5	111.8±10.5	9.4	81.5	0.1	0.3
DFEBB	117.2±1.3	0.6	102.9±8.9	8.6	83.1	0.1	0.3
5CT	100.9±4.0	3.9	125.0±19.5	15.6	94.0	0.1	0.3
DFMDFPTPB	117.2±2.7	2.3	120.6±12.7	10.5	92.6	0.1	0.3
TDFPCT	112.9±1.6	0.8	106.7±10.3	9.6	86.2	0.1	0.3
TFPrBB	119.6±2.9	1.2	101.7±5.8	5.7	77.9	0.1	0.3
PDTPMTT	116.5±0.9	0.8	119.2±12.3	10.3	87.6	0.1	0.3
DMTMDETPB	121.4±3.0	2.2	117.4±13.2	11.3	87.2	0.1	0.3
DPrBB	109.4±2.4	1.0	101.6±7.5	7.4	87.2	0.1	0.3
DBBB	117.2±3.8	2.6	104.4±5.6	5.4	81.5	0.1	0.3
DPeBB	113.9±1.3	0.4	102.7±6.7	6.5	82.0	0.1	0.3
BPCHB	113.6±4.6	4.1	97.7±3.3	3.3	89.2	0.1	0.3
PCPCB	115.3±5.8	3.9	108.6±0.5	0.5	90.1	0.1	0.3

1. Matrix effect (ME%) was evaluated in blubber at 10 μg/L using three parallel spiked replicates, with ME% = (signal of pre-extraction spike/signal of post-extraction spike) × 100%.

2. Recoveries were determined in blubber matrix (n = 3 for each spike level).

3. Recovery (%) was calculated as (measured concentration / spiked concentration) × 100%.

4. RSD (%) was calculated as (standard deviation / mean) × 100% based on three recovery values.

5. Instrument Detection Limit (IDL) was defined based on the lowest point of the instrument calibration curve that met the targeted S/N criteria and linearity requirement.

6. The MLOQ was then defined as three times this instrumental quantitation limit.

Table S6. Spike recoveries of 62 LCMs assessed in liver, muscle, kindey, brain matrix (spiked concentration = 10 µg/L)

Abbr.	Recoveries			
	Liver	Muscle	Kindey	Brain
DPB	72.4	80.4	72.0	86.1
MOPrCHB	86.5	85.0	78.2	73.0
PPB	79.0	77.7	76.2	85.5
EOPrCHB	83.2	79.6	82.3	87.7
2CB	83.8	75.2	79.2	88.7
EBDFMB	81.2	78.6	82.1	87.6
EDFPrB	85.0	78.9	84.7	85.3
MPB	88.4	80.7	89.6	86.9
DFMPMB	89.3	80.6	90.4	89.5
PeCHPrB	105.6	93.7	101.4	94.1
BDPrB	80.0	82.0	74.0	91.4
EDFPeB	87.7	85.3	87.3	93.3
EPhEMOB	92.0	87.4	90.9	95.8
3OCB	83.3	75.7	82.0	90.2
ECTFB	85.3	81.8	83.0	91.3
TFECB	81.9	81.7	78.1	87.8
ETFB	85.9	81.2	86.5	89.4
DFTMDEB	88.8	83.2	91.9	93.5
DFEB	85.2	82.9	85.2	90.3
ETeT	85.2	80.1	86.9	89.7
PCTB	84.6	81.8	85.1	87.1
EBMB	96.2	79.5	90.4	93.0
TFPrCB	86.4	83.9	86.8	93.8
TeFPrB	85.4	78.3	87.3	86.3
FPCB	82.9	86.9	82.8	91.8
DFPrCB	85.1	80.0	84.5	90.1
EDFPPB	80.1	82.0	78.9	90.0
TFrPrB	88.2	80.6	89.8	86.4
DFTDFPB	91.0	79.8	91.7	89.7
DFPrB	89.8	83.1	90.7	88.7
FPB	90.3	84.0	91.1	90.8
5OCB	65.3	69.9	48.2	82.8
TeFPT	65.5	39.3	61.1	75.5
MPCB	96.1	80.6	105.6	85.6
6OCB	98.7	79.3	97.7	97.7
DMTMDETPB	100.2	80.4	103.2	91.6
MPhBB	122.4	89.2	127.8	94.3
TFPeCB	90.3	78.1	92.0	89.3
TrFPeB	98.4	85.2	102.3	93.8

DFMPCB	99.9	81.9	101.7	89.7
EPB	102.7	83.6	106.4	93.3
DFEEPB	103.9	79.4	105.2	91.4
EDFPBB	106.5	84.5	110.7	94.0
EFPT	109.9	85.0	111.8	95.9
8OCB	111.5	89.7	110.9	113.4
EDFPB	117.3	87.3	120.8	96.6
DFPPCB	119.7	87.9	122.5	96.3
BCEDFB	112.2	86.2	114.8	97.6
BEFT	121.2	87.5	127.1	103.1
PFPT	112.2	99.7	114.2	118.6
DFEBB	117.2	92.6	118.5	108.6
5CT	100.9	102.5	96.7	137.4
DFMDFPTPB	117.2	106.0	114.7	128.8
TDFPCT	112.9	94.9	112.9	113.4
TFPrBB	119.6	95.0	119.9	105.5
PDTPMTT	116.5	105.1	115.6	127.2
DMTMDETPB	121.4	102.2	118.0	125.9
DPrBB	109.4	93.0	111.5	106.5
DBBB	117.2	98.0	115.6	108.2
DPeBB	113.9	95.1	114.4	107.1
BPCHB	113.6	94.0	109.3	100.1
PCPCB	115.3	108.8	108.7	109.0

Table S7. Spike recoveries of 62 LCMs assessed in cell culture matrix (spiked concentration = 10 μg/L)

Abbr.	Mean±SD (%)	Abbr.	Mean±SD (%)	Abbr.	Mean±SD (%)
DPB	119.2±4.3	EBMB	115.8±2.4	EDFPBB	132.0±2.2
MOPrCHB	120.8±5.2	TFPrCB	124.5±3.9	EFPT	125.3±6.5
PPB	117.4±2.5	TeFPrB	131.7±6.7	8OCB	129.0±3.1
EOPrCHB	113.0±1.4	FPCB	129.2±7.5	EDFPB	128.1±5.6
2CB	115.0±5.8	DFPrCB	126.8±12.3	DFPPCB	119.2±4.6
EBDFMB	107.8±1.8	EDFPPB	122.2±4.9	BCEDFB	127.4±3.6
EDFPrB	116.2±1.7	TFrPrB	125.2±7.5	BEFT	131.7±4.4
MPB	121.4±4.5	DFTDFPB	120.0±3.9	PFPT	126.9±9.4
DFMPMB	129.2±6.7	DFPrB	123.5±1.6	DFEBB	127.0±3.0
PeCHPrB	124.7±4.5	FPB	124.6±8.0	5CT	120.1±10.2
BDPrB	102.3±8.8	5OCB	127.1±8.3	DFMDFPTPB	129.6±4.4
EDFPeB	124.1±7.2	TeFPT	131.5±7.2	TDFPCT	126.5±3.1
EPhEMOB	125.7±6.5	MPCB	127.5±6.0	TFPrBB	116.0±8.8
3OCB	127.9±6.9	6OCB	132.6±9.0	PDTPMTT	126.4±7.1
ECTFB	128.1±2.1	DMTMDETPB	132.1±4.9	DMTMDETPB	140.3±5.1
TFECB	125.2±1.0	MPhBB	130.2±5.0	DPrBB	131.1±0.5
ETFB	118.5±10.2	TFPeCB	126.7±2.0	DBBB	118.2±10.2
DFTMDEB	127.4±6.2	TrFPeB	132.8±4.2	DPeBB	111.9±12.6
DFEB	126.9±1.4	DFMPCB	129.1±3.4	BPCHB	109.3±0.6
ETeT	122.7±9.1	EPB	129.8±4.6	PCPCB	97.4±3.7
PCTB	131.2±2.8	DFEEPB	130.0±7.4		

Table S8. The predictive toxicity of eight priority LCMs using OECD QSAR Toolbox v4.4.1.

LCM abbr.	Structure	Cramer [a]	PBT	Predicted toxicity endpoints with Rat liver S9 metabolism simulator				
				Acute toxicity	DNA binding	Protein Binding	Carcinogenicity [f]	Mutagenicity [f]
PeCHPrB	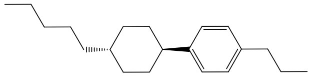	Intermediate<br>(Class II)	PB	Aldehydes<br>Benzyl Alcohols	Michael addition [b]<br>(P&M) [d]	Schiff base former [c]<br>(M) [e]	Simple aldehydes	Simple aldehydes
MOPrCHB	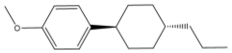	High<br>(Class III)	PvB	Aldehydes<br>Phenols<br>Benzyl Alcohols	Michael addition<br>(M);<br>Schiff base former<br>(M)	Michael addition<br>(M);<br>Schiff base former<br>(M)	alpha,beta-<br>unsaturated<br>carbonyls;<br>Quinones;<br>Simple aldehydes	alpha,beta-unsaturated<br>carbonyls;<br>Quinones;<br>Simple aldehydes;<br>H-acceptor-path3-H-<br>acceptor
EDFPPB	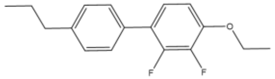	High<br>(Class III)	vPvB	Aldehydes<br>Phenols	Schiff base former<br>(M)	Schiff base former<br>(M)	Simple aldehydes	Simple aldehydes;<br>H-acceptor-path3-H-<br>acceptor
EBMB	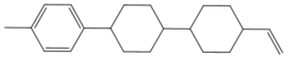	Intermediate<br>(Class II)	P	Phenols<br>Benzyle Alcohols<br>Vinyl/Allyl Alcohols	Michael addition<br>(P&M)	/	/	/
MPhBB	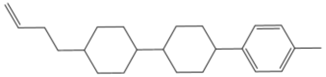	Intermediate<br>(Class II)	PB	Phenols<br>Benzyle Alcohols<br>Vinyl/Allyl Alcohols<br>Epoxides	Michael addition<br>(P&M)	Epoxides (M)	Epoxides &<br>aziridines	Epoxides & aziridines
PCTB	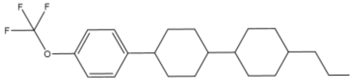	High<br>(Class III)	vPB	Benzyl Alcohols<br>Phenols	/	/	/	H-acceptor-path3-H-<br>acceptor
MPCB	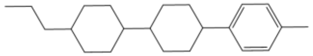	Intermediate<br>(Class II)	vPB	Benzyl Alcohols<br>Phenols	Michael addition<br>(P&M)	/	/	/
PPB	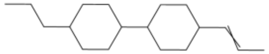	Low<br>(Class I)	vPB	Vinyl/Allyl Alcohols	/	/	/	/

[a] Toxic hazard classification by Cramar; [b] Michael addition: P450 mediated activation to Quinones and Quinone-type aromatic hydrocarbons; [c] Schiff base former: a Schiff base formation mechanism is responsible for the DNA/protein binding ability of these types of chemicals; [d] P means parent compound; [e] M means metabolites. [f] For carcinogenicity and mutagenicity, all toxic alerts are derived from M.

Table S9. The eight priority LCMs applied in the toxicity test.

LCM abbr.	Structure	FD (%) (total samples)	Maximum concentration detected in (ng/g)		Half-life in water (day)	Control group (ng/ml)		Concentration 1 (ng/ml)		Concentration 2 (ng/ml)	
			brain	muscle		Nominal	Measured	Nominal	Measured	Nominal	Measured
PeCHPrB	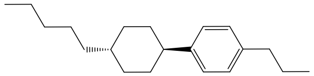	47.6	43.3	59.3	37.5	0	0	45	64	225	140
MOPrCHB	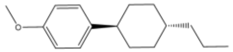	19.1	11.6	81.1	37.5	0	0	60	80	300	258
EDFPPB	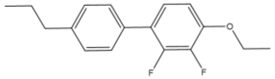	22.2	0.80	2.26	180	0	0	3	8	15	51
EBMB	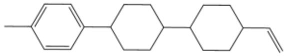	36.5	1.55	7.47	37.5	0	0	10	41	50	125
MPhBB	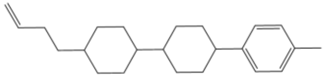	9.5	1.68	3.94	37.5	0	0	1	5	5	11
PCTB	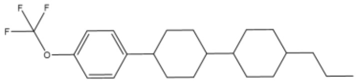	20.6	2.94	4.30	180	0	0	1	4	5	9
MPCB	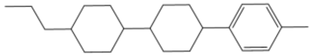	54.0	7.65	30.0	37.5	0	0	5	16	25	52
PPB	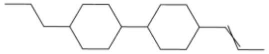	54.0	40.9	61.6	37.5	0	0	60	83	300	133

1. Exposure concentrations for individual LCM were selected based on the average concentration determined in cetacean samples.
2. Except to PeCHPrB, all the chemicals were dissolved into DMSO in stock solution. PeCHPrB was dissolved in ethanol.
3. Cells from the skin and kidney of a melon-head whale were used.
4. Skin cells and kidney cells exposed to chemicals in designed conc for 24 hours and then harvested for RNAseq and qPCR analyses .
5. Nominal: the theoretical concentration of LCM added to the culture medium; Measured: the actual concentration quantified in t he medium 24 hours after addition.

Table S10. Concentrations and relative contributions of liquid crystal monomers (LCMs) in different tissues of *NP*.

Compound	Blubber (n=15)<br>Mean±SD (DF%)<br>Median [Range]	Muscle (n=11)<br>Mean±SD (DF%)<br>Median [Range]	Brain (n=7)<br>Mean±SD (DF%)<br>Median [Range]	Liver (n=4)<br>Mean±SD (DF%)<br>Median [Range]	Kidney (n=2)<br>Mean±SD (DF%)<br>Median [Range]
ΣLCMs	116±157 (87%)	27.2±31.0 (100%)	38.7±36.8 (100%)	4.94±6.06 (75%)	0±0 (0%)
	10.8 [0-541]	22.7 [0.161-91.6]	27.9 [0.190-100]	3.19 [0-13.4]	0 [0-0]
MOPrCHB	3.96±15.3 (6.67%)	1.62±5.37 (9.09%)	1.34±3.54 (14.3%)	1.58±3.15 (25%)	0±0 (0%)
	0 [0-59.4]	0 [0-17.8]	0 [0-9.38]	0 [0-6.3]	0 [0-0]
PPB	37.5±69.3 (33.3%)	11.3±12.8 (63.6%)	18.1±16.4 (85.7%)	1.03±2.06 (25%)	0±0 (0%)
	0 [0-235]	8.28 [0-33.7]	11.9 [0-40.9]	0 [0-4.11]	0 [0-0]
EDFPPB	1.2±3.01 (20%)	0.0232±0.077 (9.09%)	0.213±0.287 (57.1%)	0±0 (0%)	0±0 (0%)
	0 [0-8.59]	0 [0-0.256]	0.163 [0-0.804]	0 [0-0]	0 [0-0]
DFMPMB	0.333±1.29 (6.67%)	0±0 (0%)	0±0 (0%)	0±0 (0%)	0±0 (0%)
	0 [0-5]	0 [0-0]	0 [0-0]	0 [0-0]	0 [0-0]
PeCHPrB	38.2±68.8 (33.3%)	11.2±13.9 (63.6%)	13±16 (85.7%)	0.458±0.917 (25%)	0±0 (0%)
	0 [0-224]	10.7 [0-46.5]	6.92 [0-43.3]	0 [0-1.83]	0 [0-0]
EPhEMOB	0.0168±0.0652 (6.67%)	0±0 (0%)	0.033±0.0873 (14.3%)	0±0 (0%)	0±0 (0%)
	0 [0-0.252]	0 [0-0]	0 [0-0.231]	0 [0-0]	0 [0-0]
ECTFB	0±0 (0%)	0±0 (0%)	0±0 (0%)	0±0 (0%)	0±0 (0%)
	0 [0-0]	0 [0-0]	0 [0-0]	0 [0-0]	0 [0-0]

TFECB	11.9±46.2 (6.67%)	0±0 (0%)	0±0 (0%)	0±0 (0%)	0±0 (0%)
	0 [0-179]	0 [0-0]	0 [0-0]	0 [0-0]	0 [0-0]
ETFB	0±0 (0%)	0.0216±0.0718 (9.09%)	0±0 (0%)	0±0 (0%)	0±0 (0%)
	0 [0-0]	0 [0-0.238]	0 [0-0]	0 [0-0]	0 [0-0]
PCTB	0.314±0.949 (13.3%)	0.626±1.33 (27.3%)	1.05±1.35 (42.9%)	0±0 (0%)	0±0 (0%)
	0 [0-3.58]	0 [0-4.3]	0 [0-2.94]	0 [0-0]	0 [0-0]
EBMB	7.85±15.7 (26.7%)	0.287±0.439 (36.4%)	0.475±0.635 (42.9%)	0.172±0.344 (25%)	0±0 (0%)
	0 [0-52.2]	0 [0-1.15]	0 [0-1.55]	0 [0-0.688]	0 [0-0]
TFPrCB	8.06±30.7 (13.3%)	0.344±0.638 (27.3%)	0.202±0.536 (14.3%)	0±0 (0%)	0±0 (0%)
	0 [0-119]	0 [0-1.75]	0 [0-1.42]	0 [0-0]	0 [0-0]
TeFPrB	0±0 (0%)	0±0 (0%)	0±0 (0%)	0.488±0.976 (25%)	0±0 (0%)
	0 [0-0]	0 [0-0]	0 [0-0]	0 [0-1.95]	0 [0-0]
FPCB	0±0 (0%)	0±0 (0%)	0.979±2.59 (14.3%)	0.818±1.64 (25%)	0±0 (0%)
	0 [0-0]	0 [0-0]	0 [0-6.85]	0 [0-3.27]	0 [0-0]
DFPrCB	0±0 (0%)	0±0 (0%)	0.0271±0.0718 (14.3%)	0±0 (0%)	0±0 (0%)
	0 [0-0]	0 [0-0]	0 [0-0.19]	0 [0-0]	0 [0-0]
EDFPPB	0.0735±0.194 (13.3%)	0±0 (0%)	0±0 (0%)	0±0 (0%)	0±0 (0%)
	0 [0-0.578]	0 [0-0]	0 [0-0]	0 [0-0]	0 [0-0]
DFTDFPB	0.0194±0.075 (6.67%)	0±0 (0%)	0±0 (0%)	0±0 (0%)	0±0 (0%)
	0 [0-0.291]	0 [0-0]	0 [0-0]	0 [0-0]	0 [0-0]

DFPrB	0±0 (0%)	0±0 (0%)	0.0858±0.227 (14.3%)	0±0 (0%)	0±0 (0%)
	0 [0-0]	0 [0-0]	0 [0-0.6]	0 [0-0]	0 [0-0]
FPB	0±0 (0%)	0±0 (0%)	0±0 (0%)	0±0 (0%)	0±0 (0%)
	0 [0-0]	0 [0-0]	0 [0-0]	0 [0-0]	0 [0-0]
5OCB	0.0554±0.215 (6.67%)	0±0 (0%)	0±0 (0%)	0±0 (0%)	0±0 (0%)
	0 [0-0.831]	0 [0-0]	0 [0-0]	0 [0-0]	0 [0-0]
TeFPT	0.165±0.64 (6.67%)	0±0 (0%)	0±0 (0%)	0±0 (0%)	0±0 (0%)
	0 [0-2.48]	0 [0-0]	0 [0-0]	0 [0-0]	0 [0-0]
MPCB	2.4±4.61 (46.7%)	1.6±1.76 (72.7%)	2.83±3.03 (85.7%)	0.114±0.227 (25%)	0±0 (0%)
	0 [0-17.6]	0.992 [0-4.5]	2.09 [0-7.65]	0 [0-0.454]	0 [0-0]
6OCB	0.0764±0.296 (6.67%)	0±0 (0%)	0±0 (0%)	0±0 (0%)	0±0 (0%)
	0 [0-1.15]	0 [0-0]	0 [0-0]	0 [0-0]	0 [0-0]
DMTMDETP	0±0 (0%)	0.0146±0.0485 (9.09%)	0±0 (0%)	0±0 (0%)	0±0 (0%)
	0 [0-0]	0 [0-0.161]	0 [0-0]	0 [0-0]	0 [0-0]
MPhBB	0.805±1.9 (20%)	0±0 (0%)	0.24±0.635 (14.3%)	0±0 (0%)	0±0 (0%)
	0 [0-6.84]	0 [0-0]	0 [0-1.68]	0 [0-0]	0 [0-0]
DFMPCB	0.116±0.449 (6.67%)	0±0 (0%)	0±0 (0%)	0±0 (0%)	0±0 (0%)
	0 [0-1.74]	0 [0-0]	0 [0-0]	0 [0-0]	0 [0-0]
EPB	0.115±0.222 (26.7%)	0.00327±0.0109 (9.09%)	0±0 (0%)	0±0 (0%)	0±0 (0%)
	0 [0-0.684]	0 [0-0.036]	0 [0-0]	0 [0-0]	0 [0-0]

8OCB	0.29±1.12 (6.67%)	0±0 (0%)	0±0 (0%)	0±0 (0%)	0±0 (0%)
	0 [0-4.35]	0 [0-0]	0 [0-0]	0 [0-0]	0 [0-0]
EDFPB	0.159±0.616 (6.67%)	0±0 (0%)	0±0 (0%)	0±0 (0%)	0±0 (0%)
	0 [0-2.38]	0 [0-0]	0 [0-0]	0 [0-0]	0 [0-0]
5CT	0.15±0.579 (6.67%)	0±0 (0%)	0±0 (0%)	0±0 (0%)	0±0 (0%)
	0 [0-2.24]	0 [0-0]	0 [0-0]	0 [0-0]	0 [0-0]
DFMDFPTPB	0.0557±0.216 (6.67%)	0±0 (0%)	0±0 (0%)	0±0 (0%)	0±0 (0%)
	0 [0-0.836]	0 [0-0]	0 [0-0]	0 [0-0]	0 [0-0]
DMTMDETPB	0.167±0.365 (20%)	0.036±0.12 (9.09%)	0±0 (0%)	0±0 (0%)	0±0 (0%)
	0 [0-1.1]	0 [0-0.396]	0 [0-0]	0 [0-0]	0 [0-0]
DBBB	0.0616±0.239 (6.67%)	0±0 (0%)	0±0 (0%)	0±0 (0%)	0±0 (0%)
	0 [0-0.924]	0 [0-0]	0 [0-0]	0 [0-0]	0 [0-0]
DPeBB	0.141±0.546 (6.67%)	0±0 (0%)	0±0 (0%)	0±0 (0%)	0±0 (0%)
	0 [0-2.12]	0 [0-0]	0 [0-0]	0 [0-0]	0 [0-0]
PCPCB	1.77±3.69 (40%)	0.166±0.37 (18.2%)	0±0 (0%)	0.29±0.58 (25%)	0±0 (0%)
	0 [0-13.8]	0 [0-0.916]	0 [0-0]	0 [0-1.16]	0 [0-0]

Table S11. Concentrations and relative contributions of liquid crystal monomers (LCMs) in different tissues of *SC*.

Compound	Blubber (n=8)<br>Mean±SD (DF%)<br>Median [Range]	Muscle (n=12)<br>Mean±SD (DF%)<br>Median [Range]	Brain (n=2)<br>Mean±SD (DF%)<br>Median [Range]	Liver (n=1)<br>Mean±SD (DF%)<br>Median [Range]	Kidney (n=1)<br>Mean±SD (DF%)<br>Median [Range]
Σ LCMs	102±118 (87.5%)	66.6±68.5 (91.7%)	7.75±11.0 (50%)	6.70±NA (100%)	1.46±NA (100%)
	74.3 [0-343]	58.9 [0-212]	7.75 [0-15.5]	6.70 [6.70-6.70]	1.46 [1.46-1.46]
MOPrCHB	22.2±51.7 (25%)	8.36±23.5 (41.7%)	5.78±8.18 (50%)	0±NA (0%)	0±NA (0%)
	0 [0-147]	0 [0-81.1]	5.78 [0-11.6]	0 [0-0]	0 [0-0]
PPB	45.2±63.6 (50%)	29.5±26.4 (75%)	0±0 (0%)	4.37±NA (100%)	0±NA (0%)
	13.9 [0-150]	31.3 [0-61.6]	0 [0-0]	4.37 [4.37-4.37]	0 [0-0]
EDFPPB	1.28±3.16 (25%)	0.303±0.505 (33.3%)	0±0 (0%)	0±NA (0%)	0±NA (0%)
	0 [0-9.02]	0 [0-1.35]	0 [0-0]	0 [0-0]	0 [0-0]
DFMPMB	0.819±2.14 (25%)	0±0 (0%)	0±0 (0%)	0±NA (0%)	0±NA (0%)
	0 [0-6.11]	0 [0-0]	0 [0-0]	0 [0-0]	0 [0-0]
PeCHPrB	14.9±17.3 (50%)	18.4±22 (58.3%)	0±0 (0%)	0±NA (0%)	0±NA (0%)
	10 [0-39.5]	7.49 [0-59.3]	0 [0-0]	0 [0-0]	0 [0-0]
ECTFB	9.49±26.8 (12.5%)	0±0 (0%)	0±0 (0%)	0±NA (0%)	0±NA (0%)
	0 [0-75.9]	0 [0-0]	0 [0-0]	0 [0-0]	0 [0-0]
PCTB	0.189±0.535 (12.5%)	0.399±0.909 (25%)	0.442±0.625 (50%)	0±NA (0%)	0±NA (0%)
	0 [0-1.51]	0 [0-3.03]	0.442 [0-0.884]	0 [0-0]	0 [0-0]
EBMB	1.47±2.72 (25%)	1.98±2.71 (58.3%)	0.338±0.478 (50%)	1.42±NA (100%)	0±NA (0%)

	0 [0-6.12]	1.05 [0-7.47]	0.338 [0-0.676]	1.42 [1.42-1.42]	0 [0-0]
TFPrCB	0±0 (0%)	0.238±0.556 (16.7%)	0±0 (0%)	0±NA (0%)	0±NA (0%)
	0 [0-0]	0 [0-1.49]	0 [0-0]	0 [0-0]	0 [0-0]
TeFPrB	1.28±3.61 (12.5%)	0±0 (0%)	0±0 (0%)	0±NA (0%)	0±NA (0%)
	0 [0-10.2]	0 [0-0]	0 [0-0]	0 [0-0]	0 [0-0]
DFPrCB	1.26±3.56 (12.5%)	0±0 (0%)	0±0 (0%)	0±NA (0%)	0±NA (0%)
	0 [0-10.1]	0 [0-0]	0 [0-0]	0 [0-0]	0 [0-0]
EBDFMB	0±0 (0%)	0.01±0.0348 (8.33%)	0±0 (0%)	0±NA (0%)	0±NA (0%)
	0 [0-0]	0 [0-0.12]	0 [0-0]	0 [0-0]	0 [0-0]
TFrPrB	1.26±3.56 (12.5%)	0±0 (0%)	0±0 (0%)	0±NA (0%)	0±NA (0%)
	0 [0-10.1]	0 [0-0]	0 [0-0]	0 [0-0]	0 [0-0]
FPB	0±0 (0%)	0.0295±0.102 (8.33%)	0±0 (0%)	0±NA (0%)	0±NA (0%)
	0 [0-0]	0 [0-0.354]	0 [0-0]	0 [0-0]	0 [0-0]
MPCB	1.54±2.79 (37.5%)	5.17±8.75 (58.3%)	1.19±1.68 (50%)	0.78±NA (100%)	0±NA (0%)
	0 [0-7.99]	1.3 [0-30]	1.19 [0-2.37]	0.78 [0.78-0.78]	0 [0-0]
MPhBB	0.311±0.881 (12.5%)	0.328±1.14 (8.33%)	0±0 (0%)	0±NA (0%)	0±NA (0%)
	0 [0-2.49]	0 [0-3.94]	0 [0-0]	0 [0-0]	0 [0-0]
EPB	0.0579±0.112 (25%)	0.128±0.307 (25%)	0±0 (0%)	0.134±NA (100%)	0±NA (0%)
	0 [0-0.289]	0 [0-1.06]	0 [0-0]	0.134 [0.134-0.134]	0 [0-0]
DFEEPB	0.11±0.312 (12.5%)	0±0 (0%)	0±0 (0%)	0±NA (0%)	0±NA (0%)
	0 [0-0.883]	0 [0-0]	0 [0-0]	0 [0-0]	0 [0-0]

8OCB	0.25±0.707 (12.5%)	1.34±4.44 (16.7%)	0±0 (0%)	0±NA (0%)	0±NA (0%)
	0 [0-2]	0 [0-15.4]	0 [0-0]	0 [0-0]	0 [0-0]
PFPT	0.323±0.913 (12.5%)	0±0 (0%)	0±0 (0%)	0±NA (0%)	0±NA (0%)
	0 [0-2.58]	0 [0-0]	0 [0-0]	0 [0-0]	0 [0-0]
DMTMDETPB	0±0 (0%)	0.0335±0.116 (8.33%)	0±0 (0%)	0±NA (0%)	0±NA (0%)
	0 [0-0]	0 [0-0.402]	0 [0-0]	0 [0-0]	0 [0-0]
DPeBB	0±0 (0%)	0.122±0.421 (8.33%)	0±0 (0%)	0±NA (0%)	1.46±NA (100%)
	0 [0-0]	0 [0-1.46]	0 [0-0]	0 [0-0]	1.46 [1.46-1.46]
PCPCB	0±0 (0%)	0.167±0.579 (8.33%)	0±0 (0%)	0±NA (0%)	0±NA (0%)
	0 [0-0]	0 [0-2.01]	0 [0-0]	0 [0-0]	0 [0-0]

Table S12. Formula and the retention time of the suspect liquid crystal monomers (LCMs) in marine cetacean samples.

No.	Formula	Retention time (Time)	Expected m/z	Measured m/z	Delta m/z
1	$C_{17}H_{30}$	5.99	234.2342	234.2344	0.68
2	$C_{16}H_{18}$	6.05	210.1403	210.1404	0.6
3	$C_{17}H_{28}$	6.25	232.2186	232.2187	0.66
4	$C_{18}H_{32}$	6.28	248.2498	248.2504	2.02
5	$C_{15}H_{19}N$	6.5	213.1512	213.1512	–0.07
6	$C_{18}H_{30}$	6.52	246.2342	246.2344	0.83
7	$C_{17}H_{24}O_{3}$	6.85	276.172	276.1717	–1.12
8	$C_{19}H_{34}$	6.92	262.2655	262.2661	2.11
9	$C_{16}H_{26}$	7.19	218.2029	218.2031	0.82
10	$C_{15}H_{28}$	7.63	208.2186	208.219	1.99
11	$C_{16}H_{28}$	9.8	220.2186	220.2186	0.22
12	$C_{17}H_{16}$	10.96	220.1246	220.1247	0.2
13	$C_{18}H_{18}$	10.96	234.1403	234.1405	0.87
14	$C_{15}H_{28}$	12.01	208.2186	208.219	2.35
15	$C_{24}H_{32}O_{3}$	12.78	368.2346	368.2346	–0.11
16	$C_{18}H_{18}$	13.17	234.1403	234.1403	0.15
17	$C_{25}H_{37}F_{3}$	13.54	394.2842	394.2825	–4.21
18	$C_{16}H_{24}$	13.98	216.1872	216.1867	–2.37
19	$C_{26}H_{32}F_{2}$	15.4	382.2464	382.2471	1.81
20	$C_{21}H_{32}F_{2}O$	15.56	338.2416	338.2407	–2.51
21	$C_{16}H_{30}O$	15.58	238.2291	238.2285	–2.79
22	$C_{18}H_{26}F_{2}O$	15.58	296.1946	296.1945	–0.44
23	$C_{18}H_{21}F$	15.65	256.1622	256.1631	3.42
24	$C_{17}H_{19}F$	15.66	242.1465	242.1474	3.47
25	$C_{27}H_{33}F_{3}$	15.76	414.252	414.2534	3.61
26	$C_{20}H_{27}F$	17.12	286.2091	286.2096	1.73
27	$C_{17}H_{15}ClF_{2}$	19.17	292.0825	292.0817	–2.82

Table S13. Error estimates with four-factor and five-factor solutions.

(a) Displacement (DISP)

	Two-factor	Three-factor	Four-factor
Error code	0	0	0
Largest decrease in Q	0	-0.007	-0.001
%dQ	0	-0.012577464	-0.003169793
Swaps by Factor (dQmax = 4)	0,0	0; 0; 0	0; 0; 0;0

(b) Bootstrap (BS)

	Factor 1	Factor 2	Unmapped
Boot Factor 1	98	0	2
Boot Factor 2	2	98	0

	Factor 1	Factor 2	Factor 3	Unmapped
Boot Factor 1	83	7	6	4
Boot Factor 2	5	84	4	7
Boot Factor 3	2	1	95	2

	Factor 1	Factor 2	Factor 3	Factor 4	Unmapped
Boot Factor 1	93	0	5	2	0
Boot Factor 2	0	93	0	4	3
Boot Factor 3	14	1	70	7	8
Boot Factor 4	10	1	0	85	4

(c) BS-DISP

	Two-factor	Three-factor	four-factor
# of cases accepted	98	70	19
% of cases accepted	98%	70%	19%
Largest decrease in Q	-14.93200016	-9.940999985	-8.19299984
%dQ	-16.21670364	-17.86179521	-25.97011468
# of decreases in Q	1	0	0
# of swaps in best fit	0	5	21
# of swaps in DISP	1	25	60
Swaps by Factor (dQmax = 0.5)	0;0	14; 54; 37; 35	29; 11; 36; 16

Table S14. The primers for the targeted genes were designed by using Primer3 with transcript data extracted from RNA sequencing results.

Gene	Forward	Reverse	Product Length
CDK1	TGAAGTGTGGCCAGAAGTGG	TTCGAGAGCAGATCCAAGCC	123
CDK2	TGCTTATCAACGCAGAGGGG	TTTCAGGTGCTCGGTACCAC	120
CDK4	TTTGACCTGATCGGACTGCC	GAAGGCAGAGATCCGCTTGT	183
CDK6	GCCGCTTTTTCGTGGAAGTT	TGGGGAGGGCAACATCTCTA	104
MCM3	GGATCCCAACTTTAGCCAGGAA	CTGCACTCACCATCTTCTCCTT	107
MCM4	TAGACGTGACTGAGCCTCTGTA	AACTTCCTGTGGGTAGCAGATG	143
CCNA2	GCACTCTACACTGTCACGGG	CGGAGCTCTGAGGTAGGTCT	120
CCND1	GTGGTCGAGGAGAGCAAACA	TCACTTTGGAGAGGAACCGC	202
TGF-β1	GGACTACTACGCCAAGGA	GAGAGCAACACAGGTTCG	150
PCNA	CACAGGACAGTGCCTTCATTTG	GTGTCACCTGCTGTATCTGGAA	161
GAPDH	ACTGCCACCCAGAAGACCG	TCCGACGCTTGCTTTACCAC	257

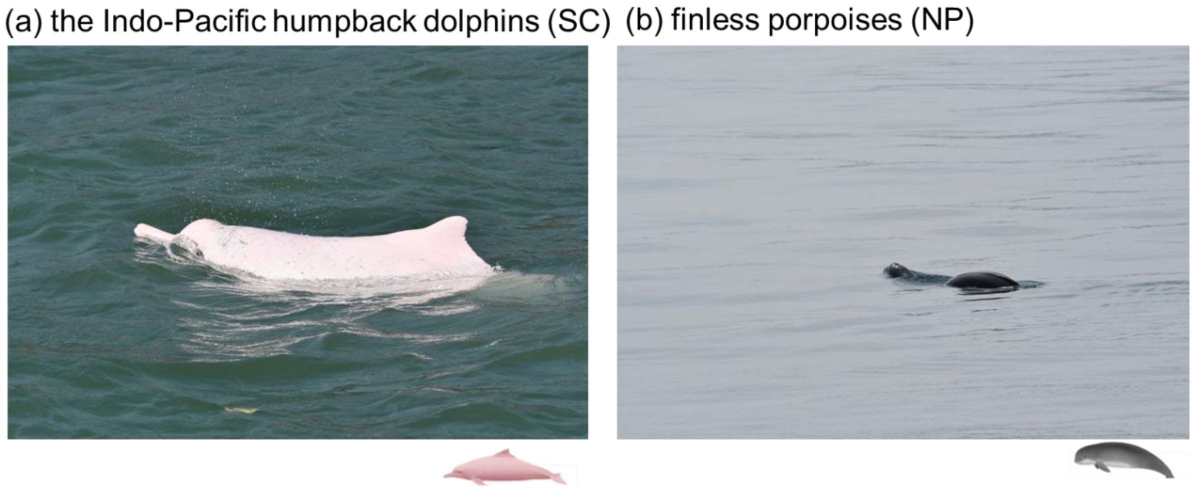

Fig. S1. The picture of two marine cetaceans: (a) Indo-Pacific humpback dolphins (*SC*) and (b) finless porpoises (*NP*).

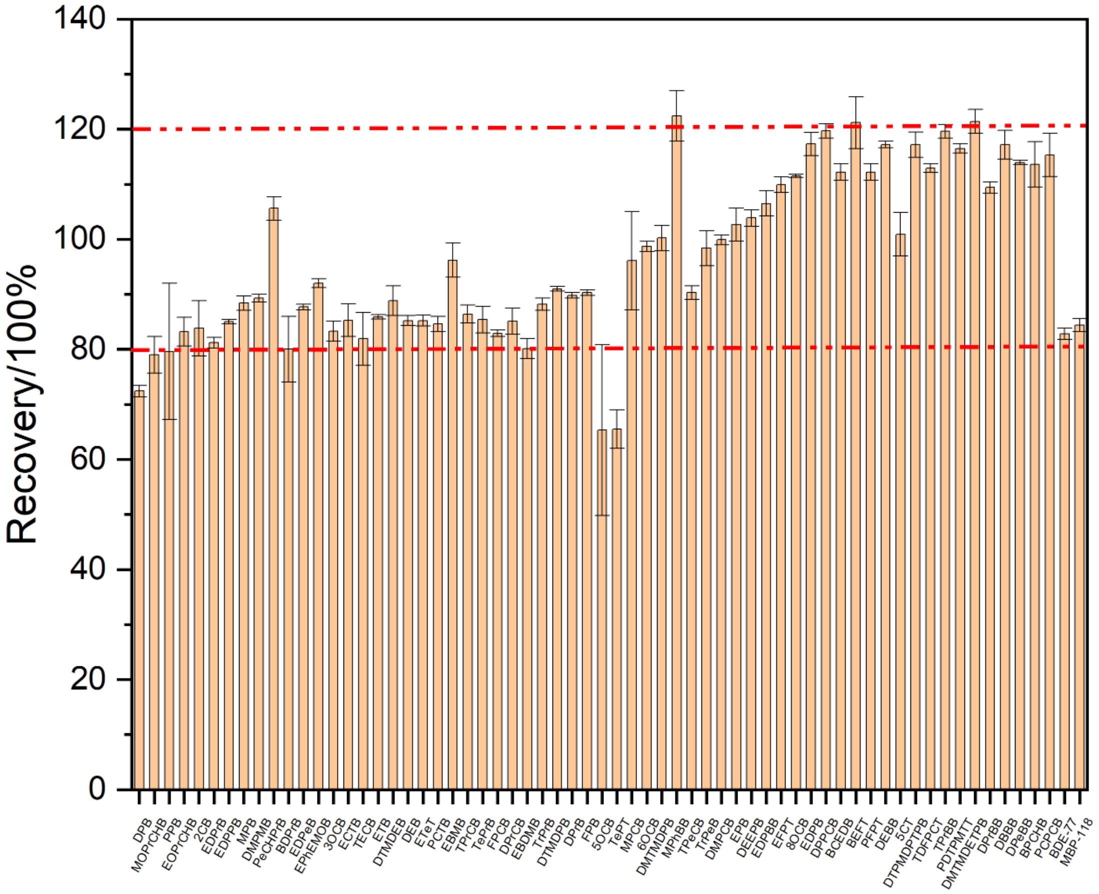

362

363 **Fig. S2. Column plot of 62 target analytes and 2 surrogate recoveries at the medium spiking level (10**
364 **ng/g) LCMs in samples.**

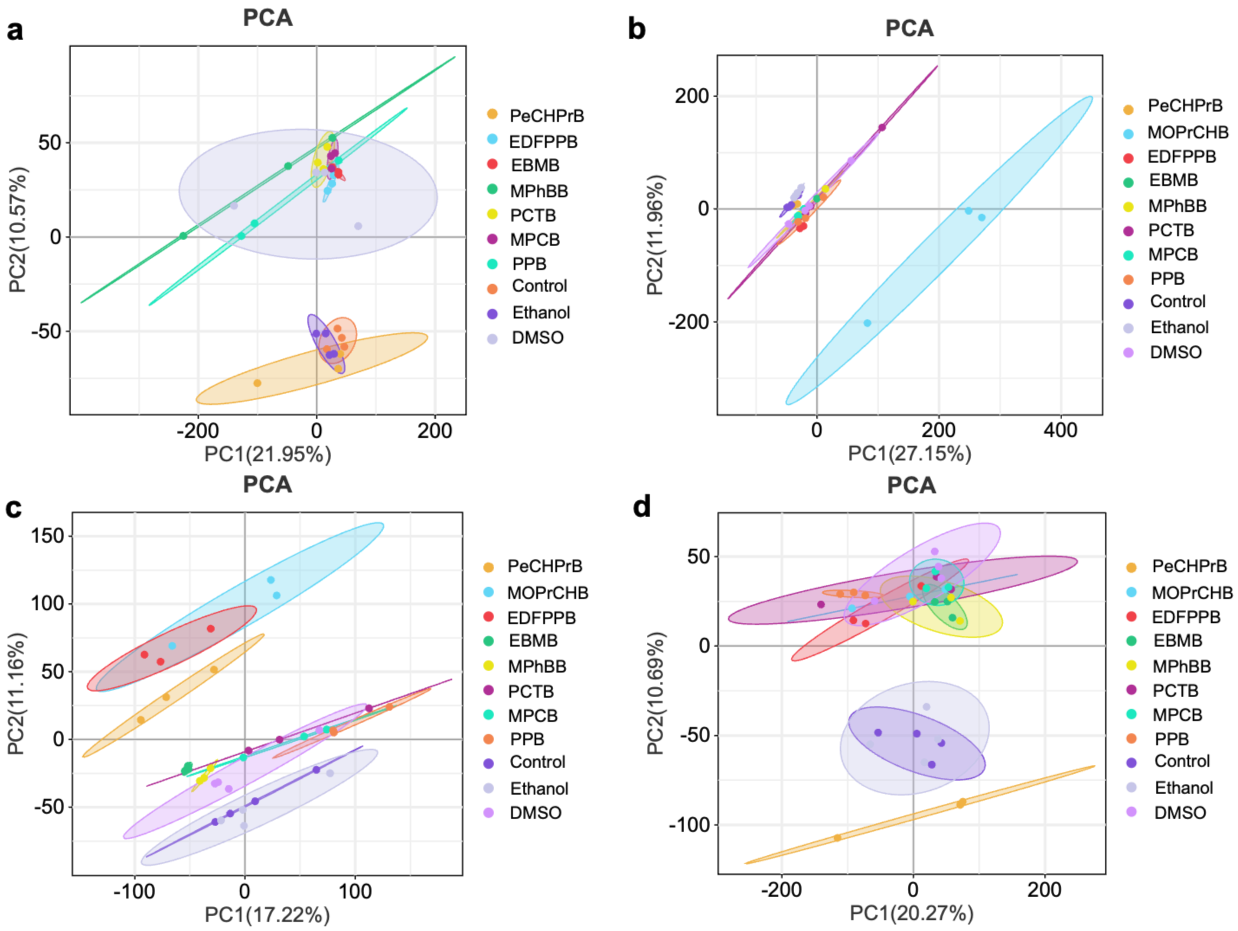

Fig. S3. Principal component analysis (PCA) of transcriptomic profiles of Melon-Head Skin Fibroblast (MHSF) and Melon-Head Kidney Fibroblast (MHKF) cells exposed to LCMs. (a) MHSF cells exposed to low concentration of LCMs. MOPrCHB was excluded because exposure caused excessive cytotoxicity and RNA integrity failed QC for RNA-seq; (b) MHSF cells exposed to high concentration of LCMs; (c) MHKF cells exposed to low concentration of LCMs; (d) MHKF cells exposed to high concentration of LCM.

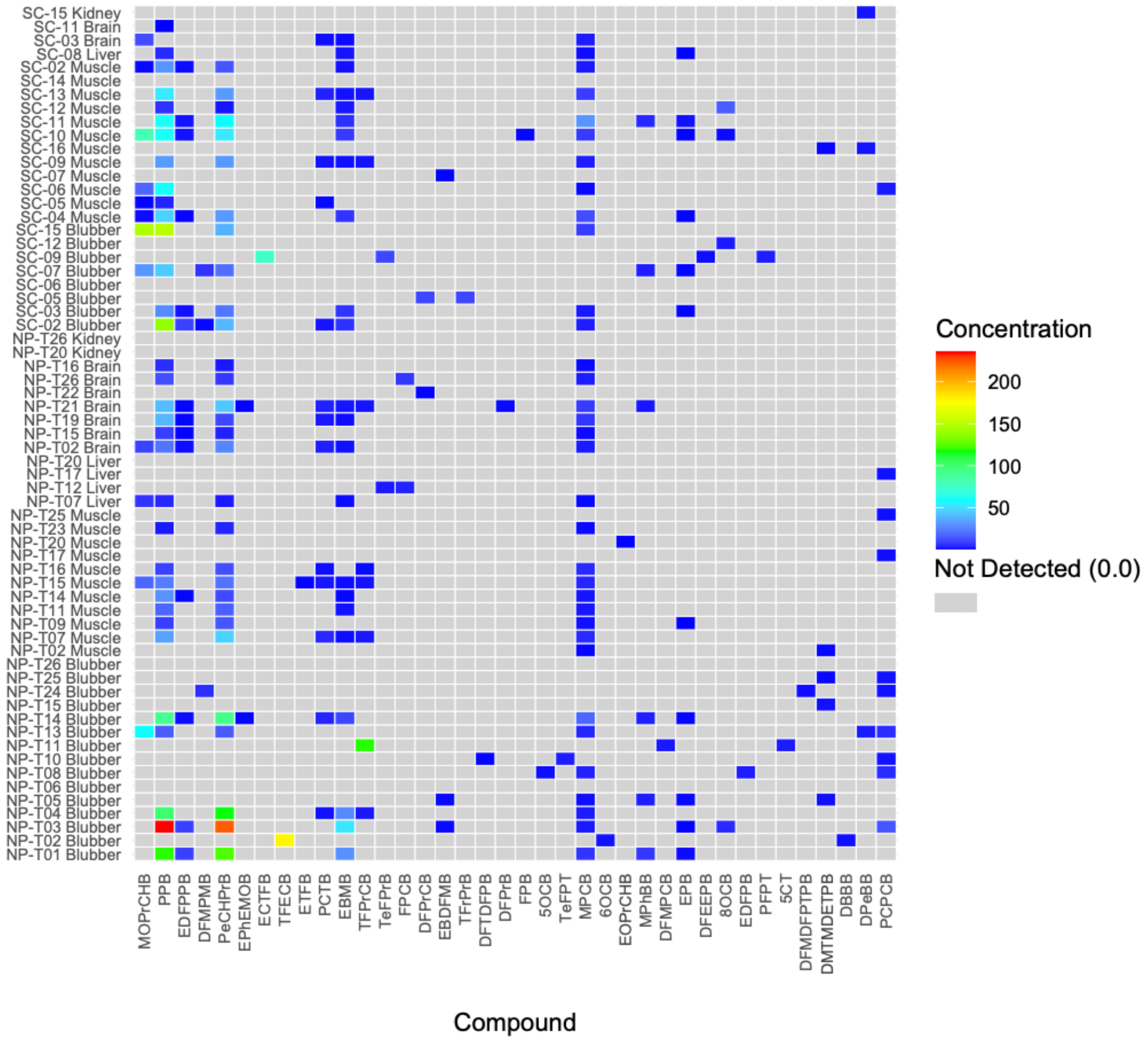

Fig. S4. Heatmap of concentrations for individual LCMs (ng/g dry weight for all tissues) in marine cetacean tissue samples collected from the studied area.

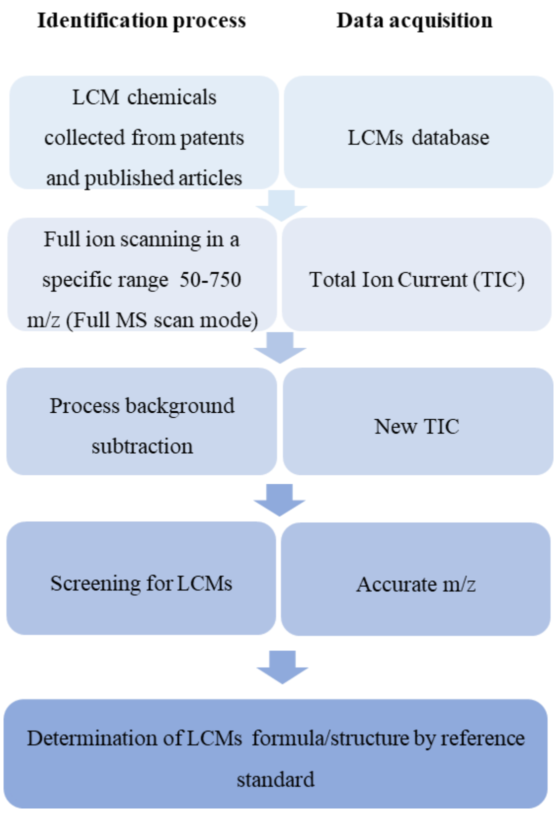

Fig. S5. The procedure of identification of new LCMs via suspect screening.

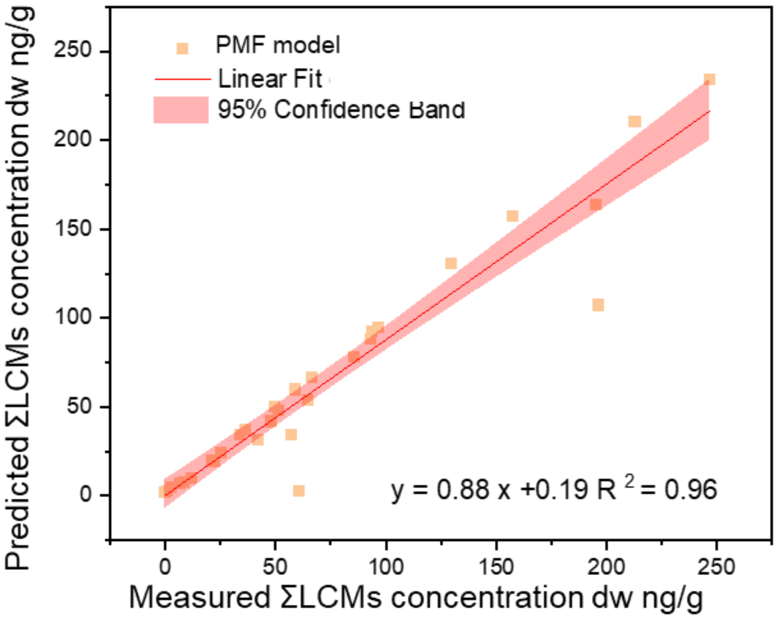

Fig. S6. Correlation of PMF modeled results with original measurements for LCMs detected from each sample.

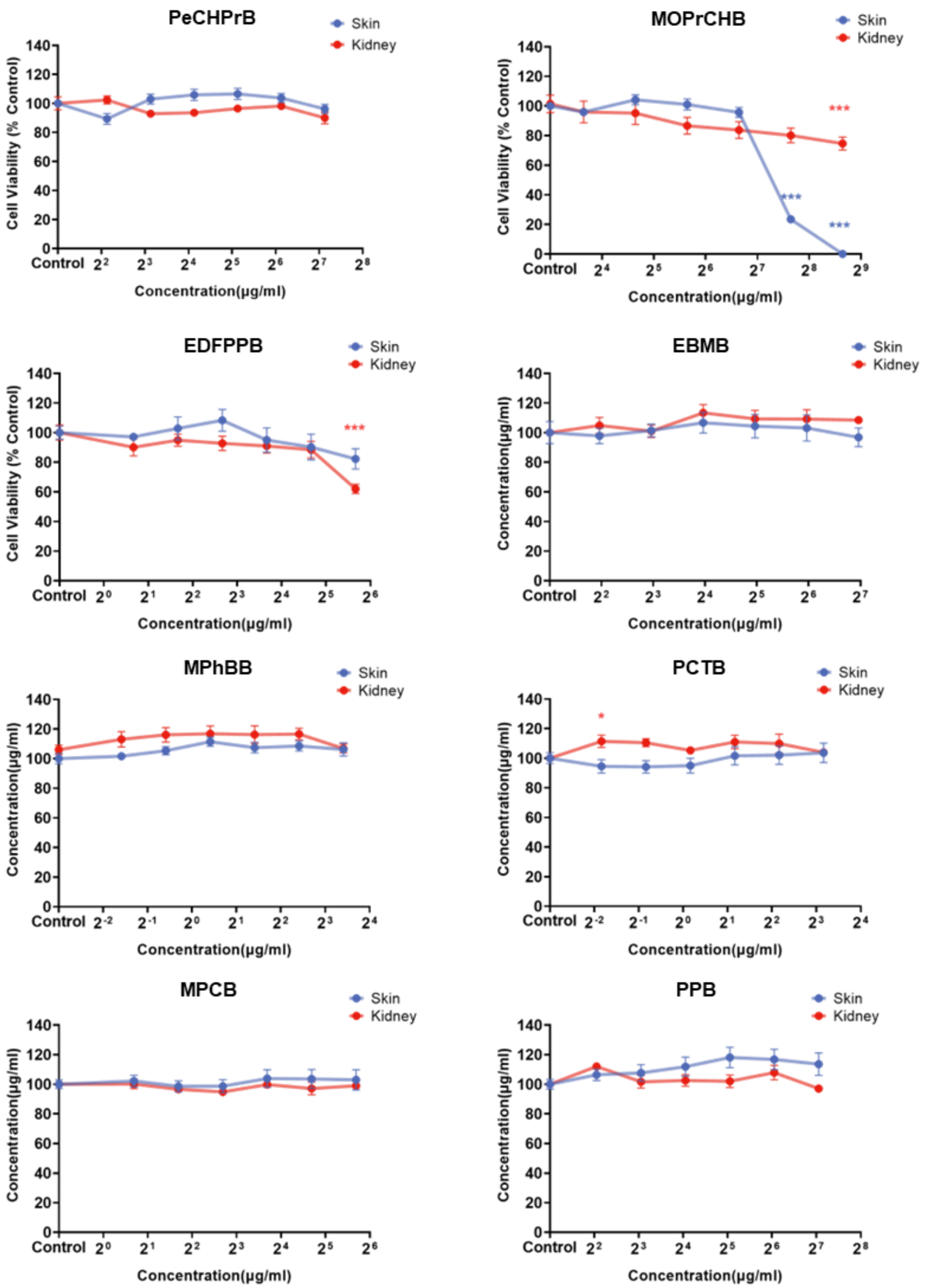

Fig. S7. The potential impact of LCMs on cell viability of Melon-Head Fibroblast (MHSF) and Melon-Head Kidney Fibroblast (MHKF) cells. Cell Counting Kit-8 (CCK-8) assay was performed with series dilution of LCMs. Cell Counting Kit-8 (CCK-8) allows sensitive colorimetric assays for the determination of cell viability in cell proliferation and cytotoxicity assays.

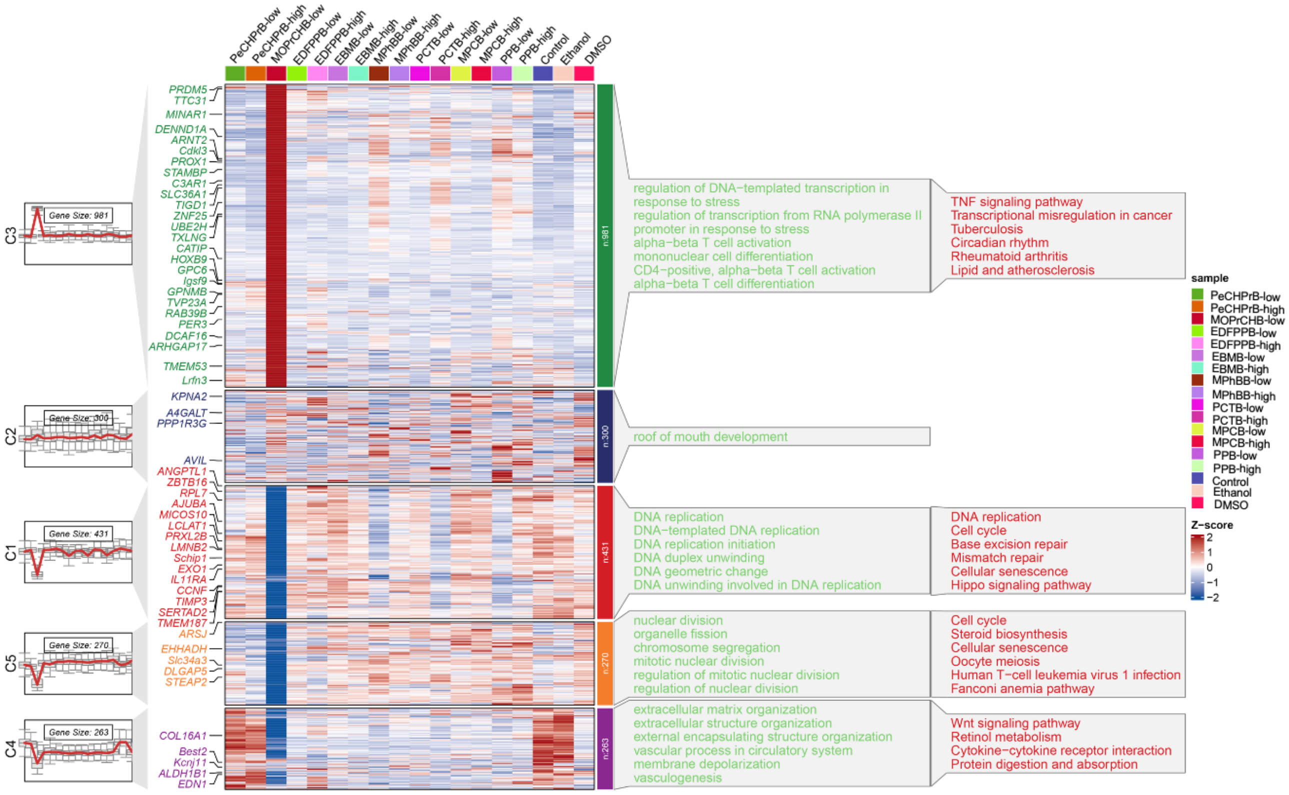

Fig. S8. Transcriptomic profiles of Melon-Head Skin Fibroblast (MHSF) cells following exposure to the eight priority LCMs. "-low" corresponds to exposure concentration 1 in Table S9, and "-high" corresponds to exposure concentration 2 in Table S9. Heatmap colors represent gene-wise z-scores across samples (red = relatively higher expression; blue = relatively lower expression). Pathway annotations summarize representative enriched functions for each gene module/cluster.

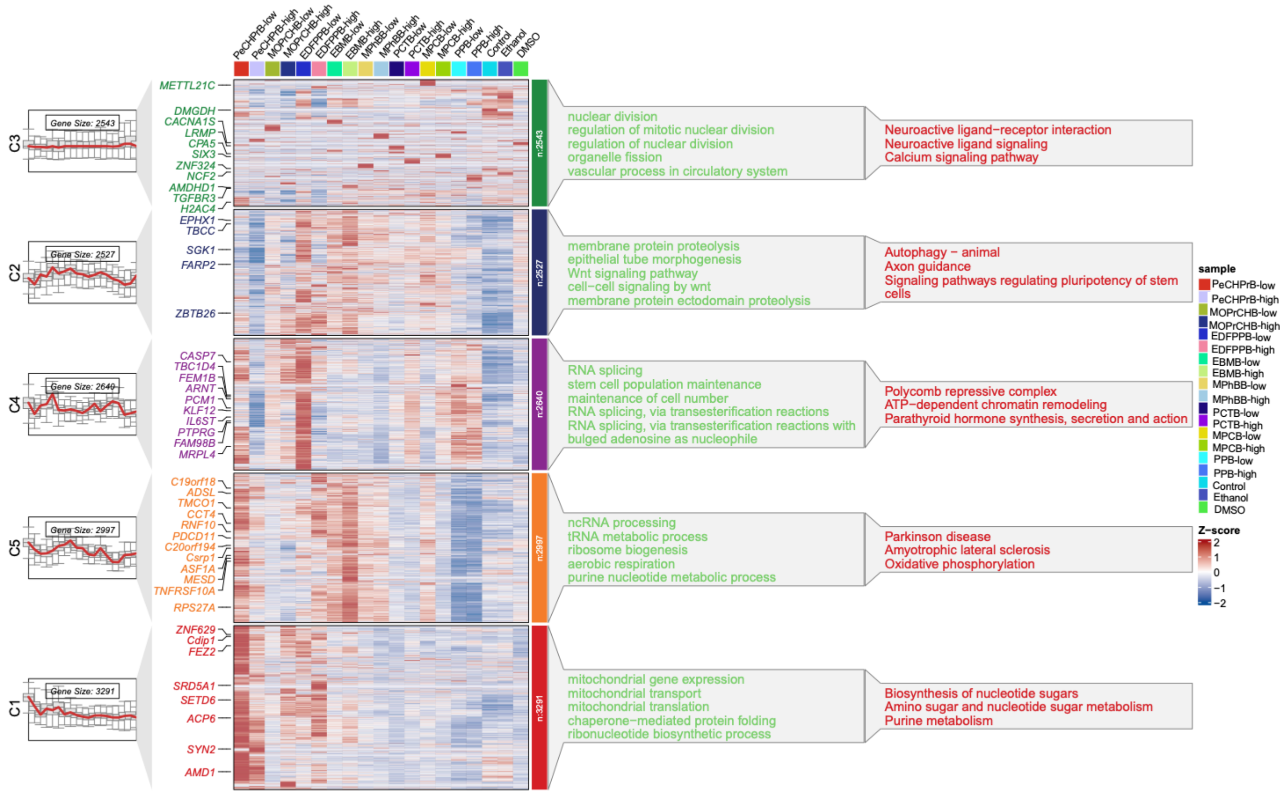

Fig. S9. Transcriptomic profiles of Melon-Head Kidney Fibroblast (MHKF) cells following exposure to the eight priority LCMs. "-low" corresponds to exposure concentration 1 in Table S9, and "-high" corresponds to exposure concentration 2 in Table S9. Heatmap colors represent gene-wise z-scores across samples (red = relatively higher expression; blue = relatively lower expression). Pathway annotations summarize representative enriched functions for each gene module/cluster.

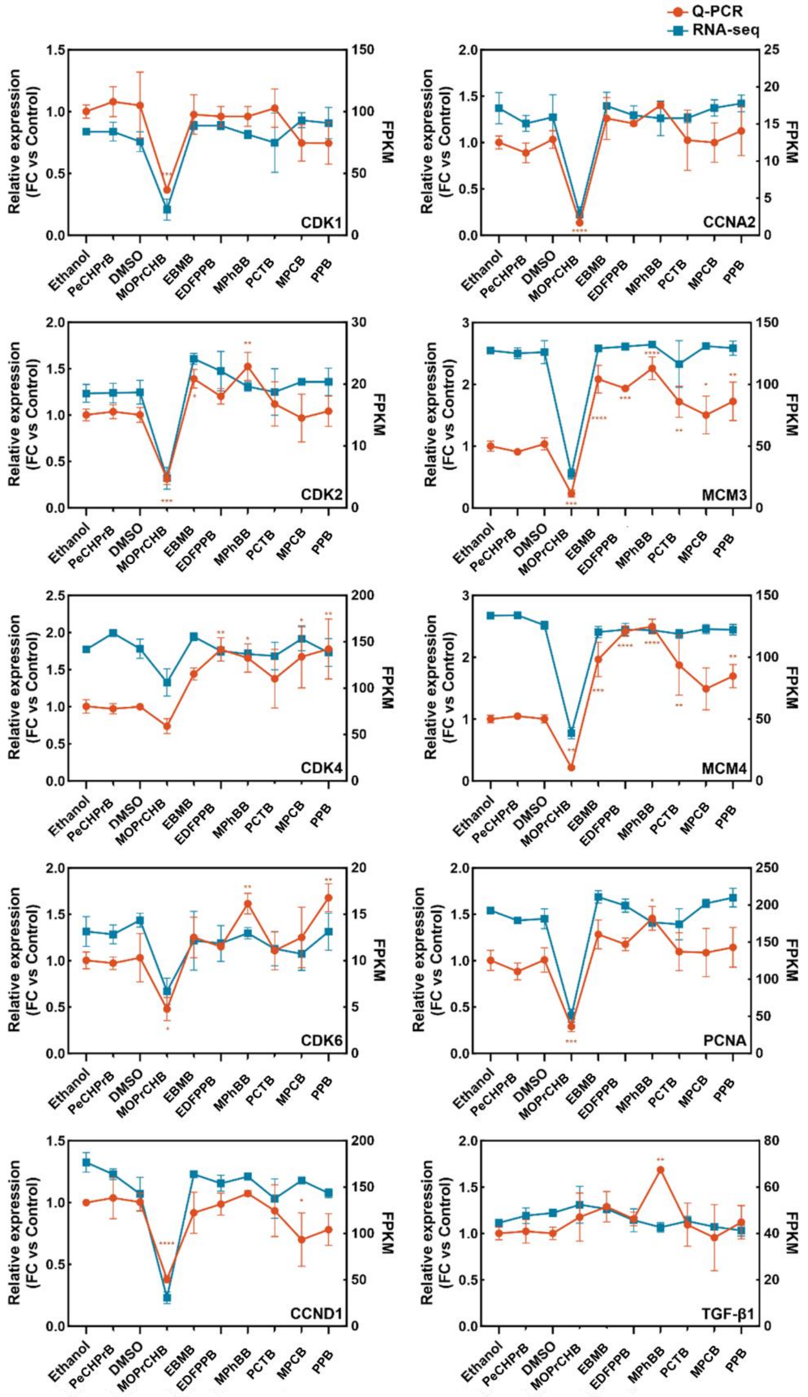

Fig. S10. Expression pattern of cell cycle related genes of Melon-Head Skin Fibroblast (MHSF) cells exposed to LCMs. CDK1: cyclin dependent kinase 1; CDK2: cyclin dependent kinase 2; CDK4: cyclin dependent kinase 4; CDK6: cyclin dependent kinase 6; CCND1: cyclin D1; CCNA2: cyclin A2; MCM3: Mini chromosome maintenance complex component 3; MCM4: Mini chromosome maintenance complex component 4; PCNA: Proliferating cell nuclear antigen; TGFB1: Transforming growth factor beta 1; Orange line and dot: relative repression level quantified by qPCR; Blue line and dot: Fragments Per Kilobase of transcript per Million mapped reads (FPKM of related genes; #: p<0.05, ##: p<0.01 when compared to solvent control group (Ethanol) by t-test with data from qPCR; *: p<0.05, **: p<0.01, ***: p<0.001, and ****: p<0.0001 when compared to solvent control group (DMSO) by one-way ANOVA followed by Dunnett multiple comparison with data from qPCR.

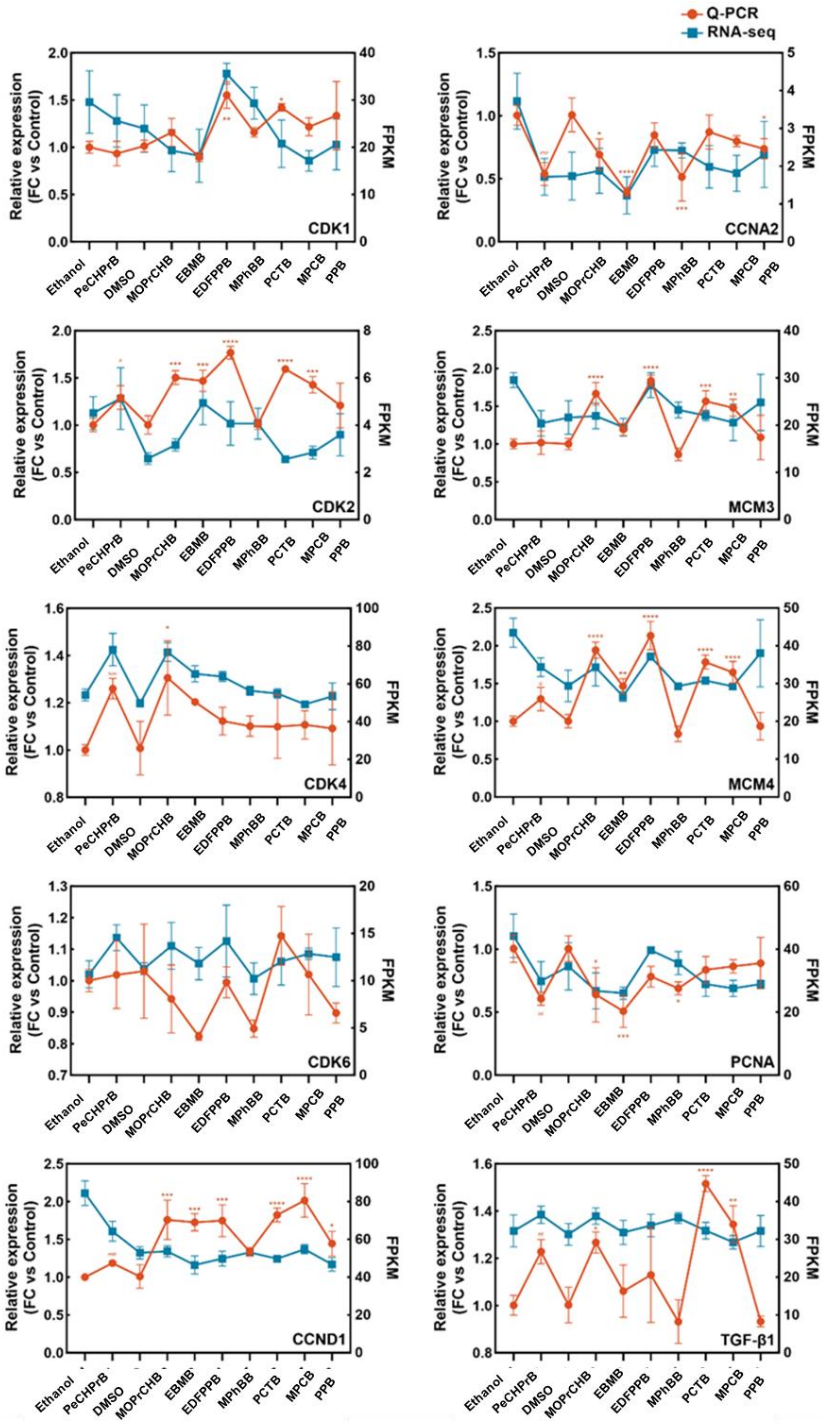

Fig. S11. Expression pattern of cell cycle related genes of Melon-Head Kidney Fibroblast (MHKF) cells exposed to LCMs. CDK1: cyclin dependent kinase 1; CDK2: cyclin dependent kinase 2; CDK4: cyclin dependent kinase 4; CDK6: cyclin dependent kinase 6; CCND1: cyclin D1; CCNA2: cyclin A2; MCM3: Mini chromosome maintenance complex component 3; MCM4: Mini chromosome maintenance complex component 4; PCNA: Proliferating cell nuclear antigen; TGFB1: Transforming growth factor beta 1; Orange line and dot: relative repression level quantified by qPCR; Blue line and dot: FPKM of related genes; #: $p<0.05$, ##: $p<0.01$ when compared to solvent control group (Ethanol) by t test with data from qPCR; *: $p<0.05$, **: $p<0.01$, ***: $p<0.001$, and ****: $p<0.0001$ when compared to solvent control group (DMSO) by one-way ANOVA followed by Dunnett multiple comparison with data from qPCR.

References

(1) Huang, S.-L.; Karczmarski, L.; Chen, J.; Zhou, R.; Lin, W.; Zhang, H.; Li, H.; Wu, Y. Demography and population trends of the largest population of Indo-Pacific humpback dolphins. *Biological Conservation* **2012**, *147* (1), 234-242.

(2) Karczmarski, L.; Huang, S.-L.; Chan, S. C. Threshold of long-term survival of a coastal delphinid in anthropogenically degraded environment: Indo-Pacific humpback dolphins in Pearl River Delta. *Scientific Reports* **2017**, *7* (1), 42900.

(3) Zhan, Y.; Jin, Q.; Lin, H.; Tao, D.; Law, L. Y.; Sun, J.; He, Y. Occurrence, behavior and fate of liquid crystal monomers in municipal wastewater. *Water Research* **2023**, *247*, 120784.

(4) Su, H.; Ren, K.; Li, R.; Li, J.; Gao, Z.; Hu, G.; Fu, P.; Su, G. Suspect screening of liquid crystal monomers (LCMs) in sediment using an established database covering 1173 LCMs. *Environmental Science & Technology* **2022**, *56* (12), 8061-8070.

(5) Tao, D.; Jin, Q.; Ruan, Y.; Zhang, K.; Jin, L.; Zhan, Y.; Su, G.; Wu, J.; Leung, K. M.; Lam, P. K. Widespread occurrence of emerging E-waste contaminants–Liquid crystal monomers in sediments of the Pearl River Estuary, China. *Journal of Hazardous Materials* **2022**, *437*, 129377.

(6) Li, Y.; Zhang, T.; Cheng, Z.; Zhang, Q.; Yang, M.; Zhao, L.; Zhang, S.; Lu, Y.; Sun, H.; Wang, L. Direct evidence on occurrence of emerging liquid crystal monomers in human serum from E-waste dismantling workers: Implication for intake assessment. *Environment International* **2022**, *169*, 107535.

(7) Zhu, M.; Su, H.; Bao, Y.; Li, J.; Su, G. Experimental determination of octanol-water partition coefficient (KOW) of 39 liquid crystal monomers (LCMs) by use of the shake-flask method. *Chemosphere* **2022**, *287*, 132407.

(8) Bjørneset, J.; Blévin, P.; Bjørnstad, P. M.; Dalmo, R. A.; Goksøyr, A.; Harju, M.; Limonta, G.; Panti, C.; Rikardsen, A. H.; Sundaram, A. Y. M.; et al. Establishment of killer whale (Orcinus orca) primary fibroblast cell cultures and their transcriptomic responses to pollutant exposure. *Environment International* **2023**, *174*, 107915.

(9) Vangipuram, M.; Ting, D.; Kim, S.; Diaz, R.; Schüle, B. Skin punch biopsy explant culture for derivation of primary human fibroblasts. *Journal of Visualized Experiments* **2013**, *7* (77), 3779. DOI: 10.3791/3779.

(10) Larsen, R. K.; Baker, J. E. Source apportionment of polycyclic aromatic hydrocarbons in the urban atmosphere: a comparison of three methods. *Environmental Science & Technology* **2003**, *37* (9), 1873-1881.

(11) Isley, C. F.; Fry, K. L.; Liu, X.; Filippelli, G. M.; Entwistle, J. A.; Martin, A. P.; Kah, M.; Meza-Figueroa, D.; Shukle, J. T.; Jabeen, K. International analysis of sources and human health risk associated with trace metal contaminants in residential indoor dust. *Environmental Science & Technology* **2021**, *56* (2), 1053-1068.

(12) Jiang, Y.-Y.; Zeng, Y.; Long, L.; Guo, J.; Lu, R.-F.; Chen, P.-P.; Pan, Z.-J.; Zhang, Y.-T.; Luo, X.-J.; Mai, B.-X. First Report on the Trophic Transfer and Priority List of Liquid Crystal Monomers in the Pearl River Estuary. *Environmental Science & Technology* **2024**, *58* (36), 16131-16141.

(13) Jin, Q.; Fan, Y.; Lu, Y.; Zhan, Y.; Sun, J.; Tao, D.; He, Y. Liquid crystal monomers in ventilation and air conditioning dust: Indoor characteristics, sources analysis and toxicity assessment. *Environment International* **2023**, 108212.

(14) Wang, J.; Nan, J.; Li, M.; Yuan, G.; Zhao, Y.; Dai, J.; Zhang, K. First evidence of contamination in aquatic organisms with organic light-emitting materials. *Environmental Science & Technology Letters* **2022**, *9* (9), 739-746.

(15) Jin, Q.; Tao, D.; Lu, Y.; Sun, J.; Lam, C. H.; Su, G.; He, Y. New insight on occurrence of liquid crystal monomers: A class of emerging e-waste pollutants in municipal landfill leachate. *Journal of Hazardous Materials* **2022**, *423*, 127146.

(16) Lin, H.; Li, X.; Qin, X.; Cao, Y.; Ruan, Y.; Leung, M. K.; Leung, K. M.; Lam, P. K.; He, Y. Particle size-dependent and route-specific exposure to liquid crystal monomers in indoor air: Implications for human health risk estimations. *Science of the Total Environment* **2024**, *908*, 168328.

(17) Jiang, Z.; Zhou, X.; Li, R.; Michal, J. J.; Zhang, S.; Dodson, M. V.; Zhang, Z.; Harland, R. M. Whole transcriptome analysis with sequencing: methods, challenges and potential solutions. *Cellular and Molecular Life Sciences* **2015**, *72*, 3425-3439.
